# Brain scans from 21,297 individuals reveal the genetic architecture of hippocampal subfield volumes

**DOI:** 10.1038/s41380-018-0262-7

**Published:** 2018-10-02

**Authors:** Dennis van der Meer, Jaroslav Rokicki, Tobias Kaufmann, Aldo Córdova-Palomera, Torgeir Moberget, Dag Alnæs, Francesco Bettella, Oleksandr Frei, Nhat Trung Doan, Ida E. Sønderby, Olav B. Smeland, Ingrid Agartz, Alessandro Bertolino, Janita Bralten, Christine L. Brandt, Jan K. Buitelaar, Srdjan Djurovic, Marjolein van Donkelaar, Erlend S. Dørum, Thomas Espeseth, Stephen V. Faraone, Guillén Fernández, Simon E. Fisher, Barbara Franke, Beathe Haatveit, Catharina A. Hartman, Pieter J. Hoekstra, Asta K. Håberg, Erik G. Jönsson, Knut K. Kolskår, Stephanie Le Hellard, Martina J. Lund, Astri J. Lundervold, Arvid Lundervold, Ingrid Melle, Jennifer Monereo Sánchez, Linn C. Norbom, Jan E. Nordvik, Lars Nyberg, Jaap Oosterlaan, Marco Papalino, Andreas Papassotiropoulos, Giulio Pergola, Dominique J. F. de Quervain, Geneviève Richard, Anne-Marthe Sanders, Pierluigi Selvaggi, Elena Shumskaya, Vidar M. Steen, Siren Tønnesen, Kristine M. Ulrichsen, Marcel P. Zwiers, Ole A. Andreassen, Lars T. Westlye, for the Alzheimer’s Disease Neuroimaging Initiative, for the Pediatric Imaging, Neurocognition and Genetics Study

**Affiliations:** 1 NORMENT, KG Jebsen Centre for Psychosis Research, Division of Mental Health and Addiction, Oslo University Hospital and Institute of Clinical Medicine, University of Oslo, Oslo, Norway; 2 Department of Psychology, University of Oslo, Oslo, Norway; 3 Department of Pediatrics, Stanford University School of Medicine, Stanford University, Stanford, USA; 4 Department of Basic Medical Sciences, Neuroscience and Sense Organs, University of Bari Aldo Moro, Bari, Italy; 5 Azienda Ospedaliero-Universitaria Consorziale Policlinico, Bari, Italy; 6 Department of Human Genetics, Radboud University Medical Center, Nijmegen, Netherlands; 7 Donders Institute for Brain, Cognition and Behaviour, Radboud University, Nijmegen, Netherlands; 8 Department of Medical Genetics, Oslo University Hospital, Oslo, Norway; 9 NORMENT, KG Jebsen Centre for Psychosis Research, Department of Clinical Science, University of Bergen, Bergen, Norway; 10 Sunnaas Rehabilitation Hospital HT, Nesodden, Norway; 11 Departments of Psychiatry and of Neuroscience and Physiology, SUNY Upstate Medical University, Syracuse, NY USA; 12 Language and Genetics Department, Max Planck Institute for Psycholinguistics, Nijmegen, Netherlands; 13 University of Groningen, University Medical Center Groningen, Interdisciplinary Center Psychopathology and Emotion Regulation, Groningen, The Netherlands; 14 University of Groningen, University Medical Center Groningen, Department of Child and Adolescent Psychiatry, Groningen, Netherlands; 15 Department of Neuromedicine and Movement Science, NTNU – Norwegian University of Science and Technology, Trondheim, Norway; 16 Department of Radiology and Nuclear Medicine, St. Olavs Hospital, Trondheim, Norway; 17 Centre for Psychiatric Research, Department of Clinical Neuroscience, Karolinska Institutet, Stockholm, Sweden; 18 Dr. Einar Martens Research Group for Biological Psychiatry, Department of Medical Genetics and Molecular Medicine, Haukeland University Hospital, Bergen, Norway; 19 Department of Biological and Medical Psychology, University of Bergen, Bergen, Norway; 20 Department of Biomedicine, University of Bergen, Bergen, Norway; 21 Departments of Radiation Sciences and Integrative Medical Biology, Umeå Center for Functional Brain Imaging (UFB), Umeå University, Umeå, Sweden; 22 Amsterdam UMC, University of Amsterdam & Vrije Universiteit Amsterdam, Emma Neuroscience Group at Emma Children’s Hospital, department of Pediatrics, Amsterdam Reproduction & Development, Amsterdam, The Netherlands; 23 Division of Molecular Neuroscience, Department of Psychology, University of Basel, Basel, Switzerland; 24 Transfaculty Research Platform Molecular and Cognitive Neurosciences, University of Basel, Basel, Switzerland; 25 Life Sciences Training Facility, Department Biozentrum, University of Basel, Basel, Switzerland; 26 Division of Cognitive Neuroscience, Department of Psychology, University of Basel, Basel, Switzerland; 27 Department of Neuroimaging, Institute of Psychiatry, Psychology and Neuroscience, King’s College London, London, UK

**Keywords:** Psychiatric disorders, Genetics, Neuroscience

## Abstract

The hippocampus is a heterogeneous structure, comprising histologically distinguishable subfields. These subfields are differentially involved in memory consolidation, spatial navigation and pattern separation, complex functions often impaired in individuals with brain disorders characterized by reduced hippocampal volume, including Alzheimer’s disease (AD) and schizophrenia. Given the structural and functional heterogeneity of the hippocampal formation, we sought to characterize the subfields’ genetic architecture. T1-weighted brain scans (*n* = 21,297, 16 cohorts) were processed with the hippocampal subfields algorithm in FreeSurfer v6.0. We ran a genome-wide association analysis on each subfield, co-varying for whole hippocampal volume. We further calculated the single-nucleotide polymorphism (SNP)-based heritability of 12 subfields, as well as their genetic correlation with each other, with other structural brain features and with AD and schizophrenia. All outcome measures were corrected for age, sex and intracranial volume. We found 15 unique genome-wide significant loci across six subfields, of which eight had not been previously linked to the hippocampus. Top SNPs were mapped to genes associated with neuronal differentiation, locomotor behaviour, schizophrenia and AD. The volumes of all the subfields were estimated to be heritable (h2 from 0.14 to 0.27, all *p* < 1 × 10^–16^) and clustered together based on their genetic correlations compared with other structural brain features. There was also evidence of genetic overlap of subicular subfield volumes with schizophrenia. We conclude that hippocampal subfields have partly distinct genetic determinants associated with specific biological processes and traits. Taking into account this specificity may increase our understanding of hippocampal neurobiology and associated pathologies.

## Introduction

The hippocampus has a key role in learning, memory and spatial navigation [1]. It is known to be particularly vulnerable to pathological conditions and implicated in several major brain disorders, most notably schizophrenia [2, 3] and Alzheimer’s disease (AD) [4].

The breadth of findings regarding the role of the hippocampus in behaviour and its nonspecific association with a range of brain disorders may result from the fact that it is a heterogeneous structure, consisting of cytoarchitecturally distinct subfields which subserve distinct functions [5, 6]. Lesion studies and intrinsic connectivity patterns support a dichotomy between an anterior section, attributed a role in anxiety-related behaviours, and more posterior regions, important for spatial processing and cognition [7]. There is also a gradient of extrinsic connectivity to both cortical and subcortical regions across the longitudinal axis superimposed on the hippocampal intrinsic connectivity organization, illustrating the complexity of hippocampal biology [8]. First-episode schizophrenia has been most strongly associated with the cornu ammonis (CA)1 region and the subiculum in the anterior hippocampus [9, 10], although with longer illness duration more posterior regions also appear affected [11]. AD is also thought to be primarily associated with volume reductions in CA1 and subiculum, with the dentate gyrus (DG) and CA3 relatively spared [12, 13], although opposing findings have been reported [14].

Imaging genetics studies have firmly established that hippocampal volume is a highly polygenic trait. Given the differences in cytoarchitecture, connectivity patterns and functions of the hippocampal subregions, it is likely to be that the volumes of the different subfields also have different genetic determinants. This is supported by gene expression studies documenting strict boundaries between subregions with respect to their transcriptional profiles [15, 16]. Genome-wide association studies (GWAS) have identified and subsequently replicated several single-nucleotide polymorphisms (SNPs) that are significantly associated with whole hippocampal volume [17–19]. These GWAS also showed that top SNPs have localized effects on specific subcortical brain regions [18] and specific hippocampal subfields [19] rather than global effects. A follow-up study failed to find evidence of genetic overlap between schizophrenia risk and whole hippocampal volume [20]. This may be partly explained by a lack of anatomical specificity in the volumetric estimates, suggesting that a more granular approach may be required.

Recently, Iglesias et al. [5] constructed a new atlas of the hippocampus, based on ultra-high-resolution magnetic resonance imaging (MRI) data using ex vivo samples [5]. This atlas has been combined with an automated segmentation algorithm and released as part of the popular neuroimaging software suite FreeSurfer v6. An initial analysis of this new software in several large-scale neuroimaging datasets established that all subfields are highly heritable, and that 11 of the 12 subfields show strong test–retest and transplatform reliability [21].

In this study, we explored the genetic architecture of each hippocampal subfield volume, as segmented by the algorithm released with FreeSurfer v6. We hypothesized that the greater specificity of these measures, compared with whole hippocampal volume, should reduce noise and allow for more sensitive detection of SNPs in genome-wide association analyses. By co-varying for whole hippocampal volume, we expected to identify associations that are specific to one or some of the subfields, allowing for a more nuanced understanding of the genetic underpinnings of this heterogeneous structure. As such, we hoped to uncover results that inform us about the individual, differing, biological functions of the subfields more than what would have been achieved by correcting solely for intracranial volume (ICV). In addition, utilizing summary statistics from previous large-scale GWAS, we sought to characterize the genetic overlap amongst the volumes of the subfields, with other subcortical and cortical regions, and with a diagnosis of schizophrenia or AD.

## Materials and methods

### Participants

We included data from 16 cohorts that had structural MRI and genome-wide genotypes available, listed in Supplementary Table S1, amounting to a total sample size of 21,297 individuals. The age range of the sample covered a large part of the lifespan (mean age 47.8 years, SD 17.3, range 3.2–91.4) and 48.3% was male. Information on individual cohorts, including brain disorder diagnoses (*n* = 1464, 6.9% of total), is given in the Supplementary Information (SI), together with figures illustrating the distributions of demographics and their relation with hippocampal volume. Each sample was collected with the participants’ written informed consent and with approval by local Institutional Review Boards.

### MRI data processing

Extended information on MRI data handling, including processing and scan quality control (QC), is given in the SI. Briefly, T1-weighted MRI volumes were processed using the standard FreeSurfer recon-all stream (v.5.3, http://surfer.nmr.mgh.harvard.edu). Hippocampal subfield volume estimates were subsequently obtained by running the novel subfield segmentation algorithm that was released as part of FreeSurfer v6.0. This algorithm employs Bayesian inference in combination with a hippocampal atlas created through manual delineation of ultra-high resolution (0.13 mm) images of ex vivo hippocampal tissue [5]. As a robustness analysis, assessing the influence of FreeSurfer version used in the initial reconstruction, we reran the main segmentation (recon -all -all) using FreeSurfer v6.0 instead of v5.3 for 50 participants. We then calculated the correlation between hippocampal subfield volume estimates obtained through the combination of FreeSurfer v5.3 and the v6.0 hippocampal segmentation algorithm with those obtained when FreeSurfer v6.0 was also used for the main segmentation. These correlations ranged from 0.87 for the parasubiculum to 0.96 for the hippocampal tail, as more thoroughly described in the SI.

### Genotyping and quality control

Genetic data were obtained at each site using commercially available genotyping platforms. We carried out phasing and imputation according to protocols in line with those applied by the ENIGMA consortium (http://enigma.ini.usc.edu), applying standard QC settings, further described in the SI. Following conventional GWAS practices, the genetic analyses were restricted to participants of European ancestry, as determined through multidimensional scaling (MDS). This was done in order to reduce heterogeneity and prevent false positives/negatives due to imputation inaccuracies and allele frequency deviations within the relatively small non-European and mixed-ancestry subsample [22, 23].

### Statistical analyses

All code used for carrying out the described analyses is available upon request from the corresponding author. We included all 12 subfields as outcome measures in the analyses, approximately from anterior to posterior: the parasubiculum, presubiculum, subiculum, CA fields 1, 2/3 and 4 (henceforth referred to as CA1, CA3 and CA4), granule cell layer of the DG, hippocampus–amygdala–transition area, fimbria (a white matter structure), the molecular layer of the DG, hippocampal fissure and the hippocampal tail. We defined whole hippocampal volume as the sum of all structures minus the hippocampal fissure. As the volumetric and genetic correlations between both hemispheres were extremely high for all structures (nearly all > 0.90), we summed the estimates of both hemispheres together to reduce the number of analyses.

Before all analyses, we regressed out the effects of scanning sites, sex, brain disorder diagnosis, age and ICV from each outcome measure. This was done through generalized additive model (GAM)-fitting in R (v2.4.0) on the total sample, estimating each outcome measure from these variables, and extracting the residuals. We further removed all individuals ± 4 SD from the mean on any of the hippocampal measures or ICV (*n* = 143, i.e., 0.67% of the total sample).

To correct for the multiple comparisons, we calculated the degree of independence between the volume estimates of the subfields plus whole hippocampus, by generating a 13 × 13 correlation matrix based on the Pearson’s correlation between all pair-wise combinations. Based on the ratio of observed eigenvalue variance to its theoretical maximum, the estimated equivalent number of independent traits in our analyses was 7.70. We therefore divided the community standard [24] nominal genome-wide significance threshold of 5 × 10^−8^ by this number, setting a threshold of 6.5 × 10^−9^.

### Genome-wide complex trait analyses

We used genome-wide complex trait analysis (GCTA) [25] to calculate SNP-based heritability of each of the GAM-residualized subfield volume estimates, as well as those of other subcortical regions and cerebral lobes produced by FreeSurfer’s subcortical [26] and cortical segmentation [27] streams. We additionally included the first four population components, calculated through MDS on the entire sample, as covariates to guard against ethnicity effects. GCTA employs a restricted maximum likelihood (REML) approach, fitting the effects of all common SNPs as random effects by a mixed linear model, to obtain an estimate of the proportion of phenotypic variance explained by genome-wide SNPs. We further applied bivariate REML to estimate the genetic correlation between all regions [28]. Before the analysis, we removed regions with high linkage disequilibrium (LD) from the genetic data and pruned it, using a sliding window approach with a window size of 50, a step size of 5 and an *R*^2^ of 0.2, leaving 133,147 SNPs. The Brain Imaging Genetics cohort was not included in these analyses, as we did not have the genetic data in-house; the sample size for these analyses was therefore *n* = 18,979.

### Genome-wide association analyses

We performed GWAS using PLINK. We chose a meta-analysis over a mega-analysis design to minimize batch effects from the cohorts, which differed in terms of mean age and other aspects of their recruitment, with virtually no loss in statistical efficiency [29]. We first carried out a GWAS within each sample for the GAM-residualized estimates of the volume of the whole hippocampus and each of the 12 subfields. We included the first four population components, calculated through MDS within each sample, as covariates. For the subfields, we also included whole hippocampal volume as a covariate. This was done to allow for the identification of associations that may be more specific to one or some of the subfields. For transparency and comparison with previous studies, we also performed a second set of GWAS for the subfields without whole hippocampal volume as a covariate, the results of which are reported in the SI. For each GWAS, we subsequently combined the within-sample results using a fixed-effect, inverse variance-weighted, meta-analysis in PLINK.

In order to assess to what degree the reported associations between SNPs and hippocampal volume were driven by the inclusion of clinical samples, we re-analysed the data excluding individuals with brain disorders (*n* = 1464, 6.9% of the total sample size used in the main genome-wide association analyses). The regression coefficients for SNPs with *P* < 1 × 10^−5^ (13,867 SNPs) from the main genome-wide analysis on whole hippocampal volume, including patients, were highly correlated with the regression coefficients from the analysis excluding patients (Pearson’s *r* = 0.87).

### Functional annotation

We used the Functional Mapping and Annotation of Genome-Wide Association Studies (FUMA) platform for functional annotation of the GWAS results [30]. Through the SNP2GENE function, significant SNPs were mapped to genes based on positional, expression quantitative trait loci, and chromatin interaction information from 18 biological data repositories and tools integrated into FUMA. The resulting set of prioritized genes was checked for overrepresentation in gene sets of biological processes and GWAS catalogues with the GENE2FUNC function, using a hypergeometric test.

### Genetic overlap with AD and schizophrenia

We applied cross-trait LD score regression (LDSR) [31] and conditional false discovery rate (FDR) analysis [32, 33] to investigate the genetic overlap of each of the subfields with schizophrenia and AD. For this, we used the summary statistics from the 2014 PGC2 schizophrenia GWAS [34] and the 2013 IGAP AD GWAS [35]. Each set of summary statistics underwent additional filtering, including the removal of all SNPs in the extended major histocompatibility complex region (chr6:25–35 Mb) and the use of only Caucasian samples. We further minimized sample overlap by rerunning the hippocampal subfield GWAS without the ADNI (Alzheimer’s Disease Neuroimaging Initiative) cohorts for comparison with the AD GWAS, and by removing the Thematically Organized Psychosis and HUman Brain INformatics cohorts from the schizophrenia GWAS. For further explanation of these two techniques, see the SI.

## Results

### SNP-based heritability

**Fig. 1:**
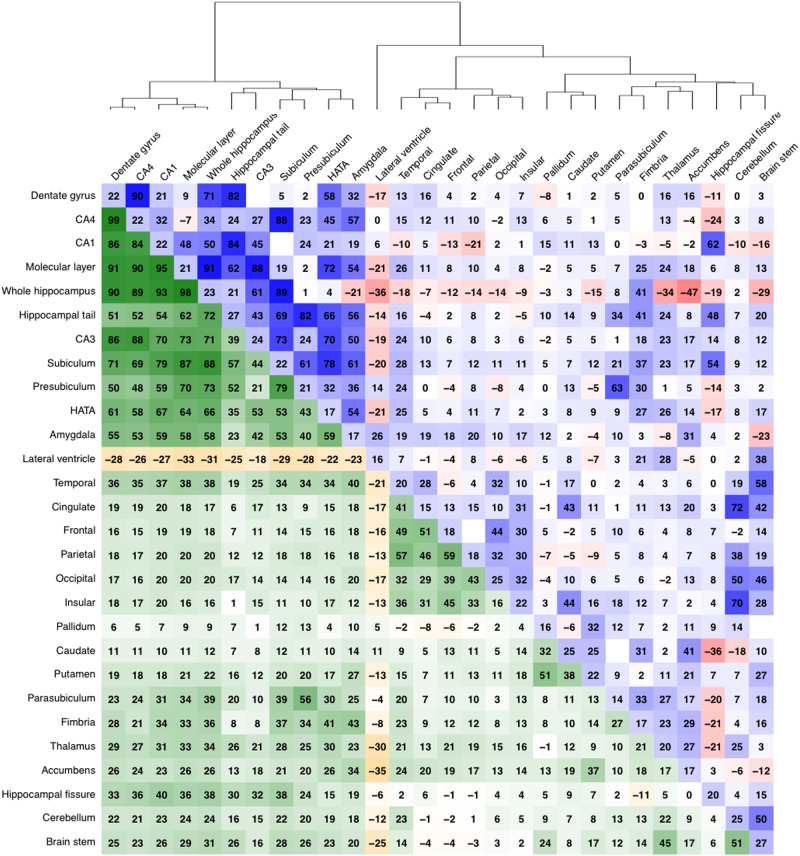
Correlation matrix of the volume estimates for the subfields as well as several other cortical and subcortical regions of interest and cerebral lobes. All correlations are multiplied by a factor 100. The volumetric correlations are shown in the lower triangle of the matrix (green–orange), the heritability estimates on the diagonal, and the genetic correlations in the upper triangle (blue–red). The order, indicated by the dendrogram on top, is determined by hierarchical clustering using Ward’s D2 method

The SNP-based heritability of each subfield’s volume estimate as well as additional regions of interest and the genetic correlations between them are shown in Fig. 1. The heritability estimates for all subfields, displayed on the plot’s diagonal, were highly significant (all *p*-values < 1 × 10^−16^), ranging from *h*^2^ = 0.14 of the parasubiculum to *h*^2^ = 0.27 for the hippocampal tail. Full test statistics of the heritability estimates for all regions are listed in Table S2. Based on their genetic correlations, most of the hippocampal subfields formed a cluster, which further included the amygdala. The cortical grey matter volumes of the cerebral lobes clustered together, as did the pallidum, caudate and putamen, i.e., basal ganglia structures.

### Genome-wide association analyses

Our GWAS of whole hippocampal volume identified eight whole-genome significant loci. Of these, three loci have not been associated with the hippocampus before, namely those with lead SNP rs7630893 at chromosome 3 within the *TFDP2* gene, lead SNP rs2303611 within the *FAM175B* gene at chromosome 10 and rs1419859 at chromosome 12 upstream of *PARP11*.

**Fig. 2:**
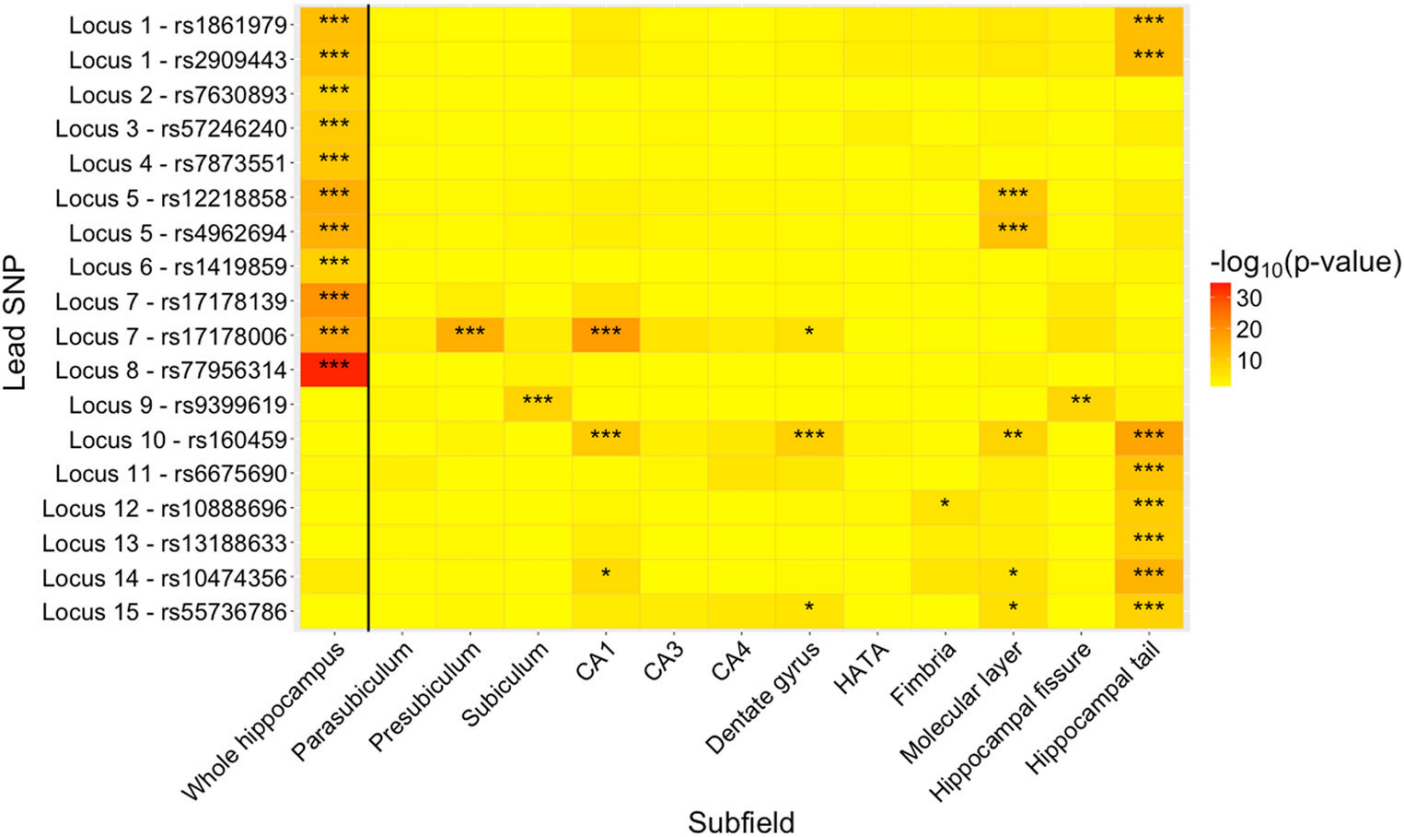
Heatmap based on the results from the genome-wide association analyses, showing the *p*-value for each of the lead SNPs reported in Table 2 (on the *y* axis) per subfield (on the *x* axis) volume. High − log_10_ *p*-values are shown in red, low values in yellow. Three stars in a field indicate the SNP reached whole-genome significance for that SNP (6.5 × 10^−9^), two stars nominal significance (5 × 10^−8^) and one star suggestive significance (1 × 10^−6^)

The GWAS per subfield, corrected for whole hippocampal volume, identified a total of ten unique loci over six subfields. Of these ten, seven were not found for the GWAS on whole hippocampal volume. See Table 1 for information on each of the lead SNPs, per structure. Figure 2 provides an overview of the distribution of the p-values per top hit over the subfields, showing that although some have global effects, others are driven by specific subfields, most prominently the hippocampal tail. QQ plots and Manhattan plots for all subfields are shown in Figure S3. Forest plots indicated that all of the lead SNPs showed comparable effect sizes across the majority of cohorts, shown in Figure S4.

**Table 1:** Whole-genome significant loci for whole hippocampal volume as well as for the subfields while co-varying for whole hippocampal volume

Structure	Unique locus	Lead SNP	A1	Chr	Position (BP)	Beta†	P-value	Mapped gene(s)	GWAS catalogue
Whole hippocampus	1	rs1861979	T	2	162845565	39.54	4.64e − 13	*SLC4A10, DPP4*	[19, 34, 37–39]
	2	rs7630893	C	3	141759380	36.18	2.55e − 09	*ATP1B3, TFDP2*	[60–62]
	3	rs57246240	A	5	66112715	36.63	9.00e − 11	*MAST4*	[19]
	4	rs7873551	C	9	119245127	-42.42	3.51e − 11	*ASTN2*	[19, 47–50, 73]
	5	rs12218858	C	10	126474200	43.75	1.06e − 15	*FAM175B, FAM53B, METTL10*	[53, 54]
	6	rs1419859	T	12	4007898	− 35.60	1.01e − 09	*PARP11*	-
	7	rs17178139	A	12	65765944	− 58.08	1.58e − 20	*WIF1, LEMD3, MSRB3*	[18, 19, 73–75]
	8	rs77956314	C	12	117323367	123.31	2.19e − 35	*RNFT2, HRK, FBXW8, TESC*	[17–19, 73]
Presubiculum	7	rs17178006	G	12	65718299	5.61	1.83e − 15	*WIF1, LEMD3, MSRB3*	[18, 19, 73–75]
Subiculum	9	rs9399619	G	6	148056480	2.31	5.87e − 09	*SAMD5*	-
CA1	7	rs17178006	G	12	65718299	− 6.48	7.76e − 19	*WIF1, LEMD3, MSRB3*	[18, 19, 73–75]
	10	rs160459	C	14	59074136	2.98	1.98e − 10	*DACT1*	-
Dentate gyrus	10	rs160459	C	14	59074136	1.53	2.04e − 09	*DACT1*	-
Molecular layer	5	rs4962694	G	10	126436717	− 1.36	3.75e − 12	*FAM175B, FAM53B, METTL10*	[53, 54]
Hippocampal tail	11	rs6675690	G	1	47945370	7.31	7.66e − 12		-
	12	rs10888696	A	1	51016603	5.22	4.04e − 10	*DMRTA2, FAF1, CDKN2C*	-
	1	rs2909443	G	2	162846439	6.11	3.08e − 13	*SLC4A10, DPP4*	[34, 37, 39, 73]
	13	rs13188633	T	5	81929360	− 5.74	7.65e − 10		-
	14	rs10474356	G	5	90816402	− 7.11	9.67e − 15		-
	15	rs55736786	T	5	93094118	− 8.59	3.23e − 09	*FAM172A, POU5F2*	-
	10	rs160459	C	14	59074136	− 7.45	1.53e − 17	*DACT1*	-

† mm^3^ volume, additive effects for each copy of allele 1 (A1). *BP* base pair, *Chr* chromosome

The set of GWAS on the subfields without co-varying for whole hippocampal volume identified a total of 35 loci over ten subfields. See Table S4 for an overview of these loci.

### Functional annotation

The location of the genome-wide significant loci, in combination with the LD structure and known biological consequences of variation in these regions, led to the prioritization of 24 genes, listed in Table 2 next to the loci that mapped onto them. Hypergeometric tests indicated that the lists of genes identified through the GWAS for both the volume of the whole hippocampus and the hippocampal tail were significantly enriched for genes associated with locomotive and exploratory behaviour. Further comparison with GWAS catalogues showed significant enrichment of AD-related genes for whole hippocampal volume, the hippocampal tail showed enrichment for schizophrenia-related genes and the molecular layer was enriched for inflammatory bowel disease.

**Table 2:** Results from the conjunctional false discovery rate (FDR) analysis of the presubiculum and subiculum GWAS summary stats with those from the schizophrenia GWAS, identifying shared loci at a conjunctional FDR < 0.05

Subfield	Locus	SNP	A1	Chr	Position (BP)	Gene	Z-score subfield	Z-score schizophrenia
Presubiculum	1	rs3790598	G	1	113196896	*CAPZA1*	− 4.37	3.63
	2	rs6427128	A	1	155026942	*ADAM15*	− 5.23	3.70
	3	rs7766356	T	6	28400538	*ZSCAN23*	− 4.20	8.16
	4	rs2554862	C	12	51202046	*ATF1*	− 3.97	− 3.52
	5	rs9966779	C	18	53620456	*AK057336*	3.72	4.85
Subiculum	1	rs11584070	A	1	150294925	*PRPF3*	4.57	4.54
	2	rs13107325	C	4	103188709	*SLC39A8*	− 4.17	− 6.27
	3	rs10087493	C	8	8373557	*PRAGMIN*	− 4.11	− 3.87
	4	rs3114896	T	16	89393562	*ANKRD11*	− 4.18	− 4.09

### Genetic overlap with AD and schizophrenia

**Fig. 3:**
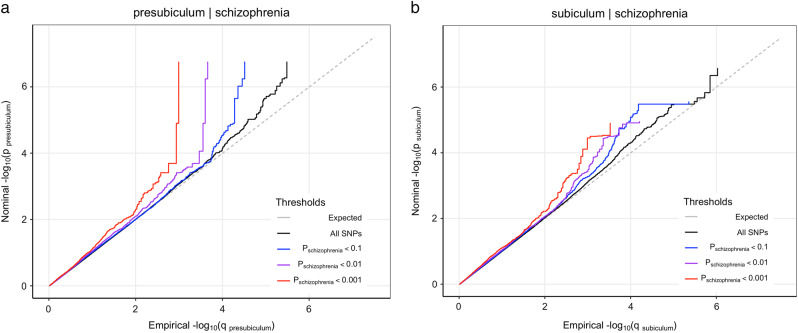
QQ plots of the *p*-values from the presubiculum and subiculum genome-wide association studies (GWAS), conditioned on those from a schizophrenia GWAS. For both subfields, there is a clear upward deflection from the expected p-value distribution (in grey) that strengthens with increasing thresholds; the black line reflects the distribution of p-values from the subfields with no schizophrenia *p*-value threshold, blue shows the distribution of *p*-values remaining at a threshold of *p* < 0.1, purple those at a threshold of *p* < 0.01 and red those at *p* < 0.001

Through LDSR, we found no significant evidence for genetic overlap of any of the hippocampal subfields with either disorder, as listed in Table S4. The conditional QQ plots did show enrichment as a function of association with schizophrenia for the presubiculum and subiculum, illustrated in Fig. 3. This is not seen for other subfields, nor when conditioning on AD (see Figure S5). The subsequent conjunctional FDR analysis for these two subfields identified respectively five and four loci overlapping with schizophrenia, described in Table 2. It is noteworthy that three out of nine hits have opposite direction of effects between subfield volume and schizophrenia, whereas the other six show the same direction of effects.

Following the lack of findings on genetic overlap between AD and the hippocampal measures, the characteristic age-related susceptibility and late onset of AD led us to hypothesize that AD-related genes may show differential associations with the hippocampal structure across the lifespan, and in particular influence hippocampal volume later in life. To test this, we investigated the association between hippocampal volume and 12 whole-genome significant loci from the discovery phase of the IGAP 2013 AD GWAS in a young and older subsample based on a median split (below and above 53.9 years of age, *n* = 9055 in each group after excluding those individuals that were part of the AD GWAS). We found that none of these SNPs were significantly associated with hippocampal volume in the younger age group, whereas three of them were significant in the older age group. See the SI for more information on these analyses and Table S6 for the full results.

## Discussion

The hippocampus complex comprises structurally and functionally distinct subfields with critical yet differential involvement in a range of behaviours and disorders. Using brain scans from 21,297 individuals, we showed that differences in the cytoarchitecture of the subfields, providing the basis for their segmentation [5], are partly driven by differences in their genetic architecture. Further, greater specificity in the phenotypes under investigation allowed for the discovery of specific genetic variants. The elucidation of their genetic architecture and identification of specific genetic variants should be helpful in better understanding the biological functions of the individual subfields and their role in the development of common brain disorders.

The SNP-based heritability estimates we obtained, ranging from 0.1 to 0.3, were comparable to those reported in previous large-scale studies of the narrow-sense heritability of subcortical structures, when corrected for ICV [20]. They also agree with findings from twin studies, showing that the larger subfields are the most heritable [21]. We further found that the genetic correlations broadly mirror the volumetric correlations, and that the subfields cluster together with the amygdala. The strength of the correlations indicates that these structures share much of their genetic determinants, yet also confirm that they do indeed have specific, individual influences. Our estimates of genetic correlations with other structures corroborate findings from a twin study that identified the same genetic clusters, with the hippocampus and amygdala clustering separately from respectively the cerebral lobes and basal ganglia structures [36].

The genome-wide association analyses per subfield supported our reasoning that greater phenotypic specificity may aid genetic discoverability; we identified several genetic variants related to the volumes of the subfields above and beyond whole hippocampal volume. We found five out of six loci reported by a recent ENIGMA hippocampal GWAS and the pattern of effects across the subfields also largely agree with their supplementary analyses of these top hits [19]. This included a locus at chromosome 2, which maps onto the *SLC4A10* and *DPP4* genes, with our subfield analyses indicating this is driven by its effect on hippocampal tail volume. This locus has also been found in GWAS of educational attainment [37], cognitive ability [38] and schizophrenia [34, 39]. Further, inhibitors of DPP4 have been shown to improve recognition memory, lower oxidative stress and increase hippocampal neurogenesis in rodents [40, 41]. The well-known locus at chromosome 12 in the *MSRB3* gene [17, 18, 36], on the other hand, appears to be mostly driven by its effect on more anterior regions, being associated with the presubiculum and *CA1*. *MSRB3*, a gene involved in anti-oxidant reactions, has recently been shown to be particularly important for pyramidal neurons specifically in *CA1* and to have lowered expression in the hippocampi of individuals with AD [42]. The other locus on chromosome 12, linked to the *HRK* gene, appears to have a global effect, not being linked to any of the subfields after correction for whole hippocampal volume. *HRK* is a pro-apoptotic gene associated with several forms of cancer [43] and reported in one GWAS of AD age of onset [44]. The two remaining replications at chromosome 5 and 9 within the *MAST4* and *ASTN2* genes also only appear for whole hippocampal volume. *MAST4* codes for a microtubule protein part of the serine/threonine kinase family, with differential expression in frontotemporal dementia [45]. *ASTN2* is thought to have a role in neuronal migration [46]. It has been repeatedly associated with migraine [47–50], as well as schizophrenia [51] and other neurodevelopmental disorders [52].

The novel loci we identified may contribute to understanding the relation between certain peripheral diseases and cognitive dysfunction. The locus at chromosome 10, within the *FAM175B* gene, has been previously associated with cocaine dependence [53] and bronchodilator responsiveness [54], as well as being reported in a recent GWAS of inflammatory bowel disease [55]. Beyond whole hippocampal volume, it was found for the molecular layer of the DG and the hippocampal tail, i.e., more posterior regions of the hippocampus. In rodents, lesions to the dorsal (corresponding to posterior in humans), but not ventral, hippocampus disrupt cocaine craving [56, 57] and cocaine administration lowers neurogenesis in the DG [58]. Chronic intestinal inflammation has been associated with altered hippocampal neurogenesis, which has been theorized to explain the link between this disease and cognitive dysfunction [59]. Another novel locus, at chromosome 3, lies within the *TFDP2* gene. This gene, with a function in cell proliferation, is well-known for its relation with kidney dysfunction [60–62]. Chronic kidney dysfunction in turn is associated with cognitive impairment and hippocampal atrophy [63].

Several genes were implicated through the GWAS on the subfields that were not identified for whole hippocampal volume, illustrating the value of studying more specific phenotypes. Through the GWAS on the hippocampal tail, we found a locus at chromosome 1 with lead SNP rs4926555, within the *FAF1* gene. The protein product of this gene regulates neuronal cell survival and apoptosis [64], as well as glucocorticoid receptor-mediated transcription in hippocampal cells [65]. The GWAS on the granule cell layer of the DG and hippocampal tail further led to the identification of a novel locus at chromosome 14 with lead SNP rs160459, mapped to the *DACT1* gene. Knockout of *DACT1* has been shown to lead to decreased dendrite complexity in cultured hippocampal pyramidal neurons [66] and its expression has been linked to tumorigenesis suppression [67].

Greater specificity in hippocampal segmentation also proved to be valuable for the investigation of genetic overlap with brain disorders. Through conditional FDR, we found signs of pleiotropy between schizophrenia and the subiculum and presubiculum, but not for other subfields. This is in line with studies showing that these anterior subfields are disproportionately affected in patients with first-episode schizophrenia [9]. Such a distinction may indicate that the relation between the subicular regions and schizophrenia is more genetically driven, whereas the global reduction of hippocampal volume seen in later disease stages is relatively stronger influenced by environmental factors and the disease process. The subsequent conjunctional FDR analyses pinpointed some specific loci that overlapped, including *SLC39A8*, a gene well-known for its high pleiotropy [68], being linked to a range of traits besides schizophrenia, including cognitive functioning [69]. These analyses also indicated that while some lead SNPs had opposing direction of effects on subfield volume versus schizophrenia, others had the same direction. These mixed directions of effects are indicative of a complex aetiology underlying the well-documented relationship between this disorder and hippocampal volume reductions. This may contribute to the scarcity of findings on most global tests of genetic overlap [20], including our own LDSR analyses, as mixed directions of effects may cancel each other out. We further found no evidence of pleiotropy between AD and any subfield in these analyses, despite the strong involvement of the hippocampus in this disorder. Follow-up analyses on age-stratified subsamples revealed that several top hits from an AD GWAS were significantly associated with hippocampal volume only in the older group, agreeing with our hypothesis that AD-related genes may influence hippocampal volume predominantly later in life. This strongly advocates for the use of age as a moderating factor in genetics studies. Our pattern of findings once again illustrates the complexity of the genetic relationships between neuroimaging measures and disorders.

Although our results are encouraging, future genetics studies may benefit from optimization of the subfield segmentation approaches. The segmentation algorithm employed here is based on an atlas created using histological and morphometric features [5]. Gene expression studies of the hippocampus have indicated that there are numerous genetic domains with clearly demarcated borders that only partly overlap with this subfield division [16]. We also found that the six subfields with significant loci were also the six largest subfields, i.e., subfield size appears positively correlated with discoverability of genetic variants. This pattern of findings likely partly reflects that the larger subfields are segmented with greater accuracy [21]. Our large age-span should also be noted in this regard, as it is currently unclear how well FreeSurfer processes scans from very young children [70]. Future studies may benefit from use of higher resolution data and/or the combination with T2-weighted images to improve segmentation accuracy [5]. Lastly, comparison of results with the literature is hindered by the differences in subfield definitions being used, harmonization is needed [71] to further improve discoverability [72].

In conclusion, in addition to providing information on the localization of the effects on the hippocampus for previously identified genetic variants, we identified novel variants that influenced specific subfields. These variants were not previously associated with hippocampal volume, yet have known roles in neuronal differentiation and neurodevelopmental disorders. Together with the estimated genetic correlations, we have shown that hippocampal subfields have partly distinct genetic determinants, associated with specific biological processes and traits, thereby providing evidence that there is value in greater specificity of the brain phenotypes under investigation. Taking into account, this specificity may aid in furthering our understanding of hippocampal neurobiology and associated functions and disorders.

## Electronic supplementary material

Revised supplemental material
