# Supplementary material for: Brain scans from 21,297 individuals reveal the genetic architecture of hippocampal subfield volumes
Source: Mol Psychiatry. 2018 Oct 2;25(11):3053–65. doi: 10.1038/s41380-018-0262-7 (PMC6445783; doi:10.1038/s41380-018-0262-7)
Supplement: Supplementary file 1 — Revised supplemental material [file 41380_2018_262_MOESM1_ESM.docx]

**Supplemental Information**

*Cohort information*

In this study, we made use of in-house samples, as well as data gathered through collaborations and data sharing platforms. In total, 16 cohorts contributed data, which are listed in Table S1 together with demographic information. Table S2 lists the number of subjects with brain disorder diagnoses per cohort. Table S3 lists additional information on each of the cohorts: their source, comments (including funding acknowledgements), and references that can be consulted for detailed descriptions of data gathering characteristics.

Table S1. Demographic information and total hippocampal volume per cohort included in the analyses

| Cohort | Sample size | Sex, % male | Age in years (SD) | Hippocampal volume in cubic millimeter (SD) |
| --- | --- | --- | --- | --- |
| PING | 413 | 51.8 | 12.1 (4.9) | 7054 (724) |
| PNC | 514 | 50.6 | 15.4 (3.6) | 7223 (667) |
| NIMAGE | 223 | 58.7 | 17.7 (3.6) | 6875 (607) |
| UBA | 1024 | 34.9 | 22.5 (3.3) | 7094 (652) |
| BIG | 2318 | 47.8 | 25.5 (10.1) | 7398 (738) |
| UNIBA | 361 | 56.5 | 28.4 (8.3) | 6879 (643) |
| TOP | 1130 | 53.1 | 32.4 (10.2) | 7289 (733) |
| HUBIN | 174 | 69.0 | 42.1 (8.1) | 7321 (712) |
| DEMGEN | 40 | 40.0 | 44.9 (22.1) | 7222 (735) |
| NCNG | 361 | 32.4 | 52.2 (17.1) | 6822 (766) |
| UKBB | 12634 | 48.1 | 55.9 (7.5) | 6941 (728) |
| STROKEMRI | 117 | 41.0 | 57.9 (15.5) | 6760 (745) |
| HUNT | 783 | 46.5 | 58.9 (4.2) | 6674 (677) |
| BETULA | 328 | 47.9 | 62.6 (13.3) | 6675 (752) |
| ADNI2 | 253 | 56.9 | 72.9 (7.1) | 6169 (916) |
| ADNI1 | 624 | 57.9 | 75.5 (6.7) | 5583 (1004) |
| All | 21297 | 48.3 | 47.8 (17.5) | 6961 (798) |

SD=standard deviation

*Table S2. Brain disorder diagnoses per cohort*

| Cohort | ADHD | | BD | Dementia | HC | MCI | MDD | Prodr. | Subthr. ADHD | SZ | SZ-SIB | SZ BD mix |
| --- | --- | --- | --- | --- | --- | --- | --- | --- | --- | --- | --- | --- |
| ADNI1 | | 0 | 0 | 137 | 179 | 308 | 0 | 0 | 0 | 0 | 0 | 0 |
| ADNI2 | | 0 | 0 | 20 | 106 | 127 | 0 | 0 | 0 | 0 | 0 | 0 |
| BETULA | | 0 | 0 | 0 | 328 | 0 | 0 | 0 | 0 | 0 | 0 | 0 |
| BIG | | 0 | 0 | 0 | 2318 | 0 | 0 | 0 | 0 | 0 | 0 | 0 |
| DEMGEN | | 0 | 0 | 0 | 40 | 0 | 0 | 0 | 0 | 0 | 0 | 0 |
| HUBIN | | 0 | 0 | 0 | 94 | 0 | 0 | 0 | 0 | 80 | 0 | 0 |
| HUNT | | 0 | 0 | 0 | 783 | 0 | 0 | 0 | 0 | 0 | 0 | 0 |
| NCNG | | 0 | 0 | 0 | 361 | 0 | 0 | 0 | 0 | 0 | 0 | 0 |
| NIMAGE | | 100 | 0 | 0 | 89 | 0 | 0 | 0 | 34 | 0 | 0 | 0 |
| PING | | 0 | 0 | 0 | 413 | 0 | 0 | 0 | 0 | 0 | 0 | 0 |
| PNC | | 0 | 0 | 0 | 514 | 0 | 0 | 0 | 0 | 0 | 0 | 0 |
| STROKEMRI | | 0 | 0 | 0 | 117 | 0 | 0 | 0 | 0 | 0 | 0 | 0 |
| TOP | | 0 | 211 | 0 | 563 | 0 | 0 | 18 | 0 | 233 | 0 | 105 |
| UBA | | 0 | 0 | 0 | 1024 | 0 | 0 | 0 | 0 | 0 | 0 | 0 |
| UKBB | | 0 | 0 | 0 | 12634 | 0 | 0 | 0 | 0 | 0 | 0 | 0 |
| UNIBA | | 0 | 0 | 0 | 270 | 0 | 2 | 0 | 0 | 72 | 17 | 0 |

ADHD=attention-deficit/hyperactivity disorder, BD=bipolar disorder, HC=healthy controls, MCI=mild cognitive impairment, MDD=major depressive disorder, Prodr.=prodromal psychosis, subtrh.ADHD= subthreshold ADHD, SZ=schizophrenia, SZ-SIB=SZ siblings

*Table S3. Cohorts included in the current study*

| **Cohort** | **Source** | **Comment** | **Reference** |
| --- | --- | --- | --- |
| ADNI1 | http://adni.loni.usc.edu/ | Data collection and sharing for this project was funded by the Alzheimer's Disease Neuroimaging Initiative (ADNI) (National Institutes of Health Grant U01 AG024904) and DOD ADNI (Department of Defense award number W81XWH-12-2-0012). ADNI is funded by the National Institute on Aging, the National Institute of Biomedical Imaging and Bioengineering, and through generous contributions from the following: AbbVie, Alzheimer’s Association; Alzheimer’s Drug Discovery Foundation; Araclon Biotech; BioClinica, Inc.; Biogen; Bristol-Myers Squibb Company; CereSpir, Inc.; Cogstate; Eisai Inc.; Elan Pharmaceuticals, Inc.; Eli Lilly and Company; EuroImmun; F. Hoffmann-La Roche Ltd and its affiliated company Genentech, Inc.; Fujirebio; GE Healthcare; IXICO Ltd.; Janssen Alzheimer Immunotherapy Research & Development, LLC.; Johnson & Johnson Pharmaceutical Research & Development LLC.; Lumosity; Lundbeck; Merck & Co., Inc.; Meso Scale Diagnostics, LLC.; NeuroRx Research; Neurotrack Technologies; Novartis Pharmaceuticals Corporation; Pfizer Inc.; Piramal Imaging; Servier; Takeda Pharmaceutical Company; and Transition Therapeutics. The Canadian Institutes of Health Research is providing funds to support ADNI clinical sites in Canada. Private sector contributions are facilitated by the Foundation for the National Institutes of Health (www.fnih.org). The grantee organization is the Northern California Institute for Research and Education, and the study is coordinated by the Alzheimer’s Therapeutic Research Institute at the University of Southern California. ADNI data are disseminated by the Laboratory for Neuro Imaging at the University of Southern California. | (1,2) |
| ADNI2 | http://adni.loni.usc.edu/ |  |  |
| BETULA | Authors | Betula was supported by a Wallenberg Scholar Grant (KAW) | (3) |
| BIG | Authors | This study used the BIG database, which was established in Nijmegen in 2007. This resource is now part of Cognomics, a joint initiative by researchers of the Donders Centre for Cognitive Neuroimaging, the Human Genetics and Cognitive Neuroscience departments of the Radboud University Medical Centre, and the Max Planck Institute for Psycholinguistics. The Cognomics Initiative is supported by the participating departments and centres and by external grants, that is, the Biobanking and Biomolecular Resources Research Infra-structure (Netherlands) (BBMRI-NL), the Hersenstichting Nederland, and the Netherlands Organisation for Scientific Research (NWO). The research leading to these results also received funding from the European CommunityÕs Seventh Framework Programme (FP7/2007Ð2 013) under grant agreements nû 602805 (Aggresso-type), nû 278948 (TACTICS), and nû 602450 (IMAGEMEND), and from the European CommunityÕs Horizon 2020 Programme (H2020/2014Ð2020) under grant agreement nû 643051 (MiND).In addition, the work was supported by a grant for the ENIGMA Consortium (grant number U54 EB020403) from the BD2K Initiative of a cross-NIH partnership. Barbara Franke is supported by a Vici grant from NWO (grant 016-130-669). The Cognomics Initiative Resource, the Brain Imaging Genetics (BIG) sample (http://www.cognomics.nl ), stems from an ongoing study, which started in 2007. The BIG sample is a collection of healthy volunteers aged 18–40 years, who participated in studies at the Donders Centre for Cognitive Neuroimaging (DCCN) of the Radboud University in Nijmegen. Subjects were of Caucasian descent with no self-reported neurological or psychiatric history, and mainly high level of education (80% with bachelor student level or higher). All participants gave written informed consent and the study was approved by the local ethics committee (CMO Region Arnhem-Nijmegen, the Netherlands). The self-reported healthy individuals underwent anatomical MRI scans, usually as part of their involvement in diverse smaller-scale studies at the DCCN. Structural T1-weighted images were acquired using MPRAGE sequence (1.0x1.0 x1.0 mm3 voxel size) with a 1.5T scanner (Sonata and Avanto, Siemens, Erlangen, Germany) or a 3T scanner (Trio and TrioTim, Siemens, Erlangen, Germany).  Genetic analyses for the BIG study were carried out at the Department of Human Genetics of the Radboud university medical center. Saliva samples were collected using Oragene kits (DNA Genotek, Kanata, Canada), and genomic DNA was extracted as specified by the manufacturer. Genome-wide genotyping was performed on three different genotyping platforms; Affymetrix Genome-Wide Human SNP Array 6.0 (Affymetrix Inc., Santa Clara, CA, USA), Infinium PsychArray-24 v1.1 BeadChip2, and Infinium OmniExpress-24 array2. Quality control steps and imputation were performed using the Ricopili Rapid Imputation Consortium Pipeline3. Pre-imputation quality control included pre-filtering of SNPs with call rate <0.95, filtering of individuals with a genotyping rate <0.98 or inbreeding coefficient >0.02, filtering of SNPs with a call rate <0.98 or Hardy-Weinbergp-value < 1e-06, removal of invariant SNPs, and removal of population ancestry outliers. SHAPEIT4 and IMPUTE2 (Howie et al., 2009) software were used for haplotype phasing and imputation with 1000 Genomes Phase 3.v5a reference data. For the current study, best-guess genotypes were inferred with a minimum probability threshold of 0.8. Post-imputation quality control included a strict SNP imputation quality threshold >=0.8, removal of duplicated and related individuals (pi hat > 0.25), removal of individuals with a call rate below 95%, and removal of SNPs with a call rate below 95%, a minor allele frequency of less than 1%, or failing the Hardy-Weinberg equilibrium test at a threshold of p<= 10–6. | (4) |
| DEMGEN | Authors |  | (5) |
| HUBIN | Authors | This study was supported by the Swedish Research Council (2006-2992, 2006-986, K2007-62X-15077-04-1, 2008-2167, K2008-62P-20597-01-3. K2010-62X-15078-07-2, K2012-61X-15078-09-3, 2017-00949), the regional agreement on medical training and clinical research between Stockholm County Council and the Karolinska Institutet, the Knut and Alice Wallenberg Foundation, and the HUBIN project | (6) |
| HUNT | https://www.ntnu.edu/hunt | The HUNT Study is a collaboration between HUNT Research Centre, Faculty of Medicine and Health Sciences, Norwegian University of Science and Technology (NTNU), Nord-Trøndelag County Council, Central Norway Regional Health Authority, and the Norwegian Institute of Public Health. HUNT-MRI and the genetic analysis were funded by grants from the Liaison Committee between the Central Norway Regional Health Authority and NTNU to principal investigator Asta Håberg, and the Norwegian National Advisory Unit for functional MRI. We thank the HUNT MRI participants, MRI technicians and the Department of Diagnostic Imaging at Levanger Hospital, Professor Lars Jacob Stovner (NTNU) and the administrative staff at HUNT. | (7,8) |
| NCNG | Authors | The sample collection was supported by grants from the Bergen Research Foundation and the University of Bergen, the Dr Einar Martens Fund, the K.G. Jebsen Foundation, the Research Council of Norway, to SLH, VMS, AJL, and TE. The authors thank Dr. Eike Wehling for recruiting participants in Bergen, and Professor Jonn-Terje Geitung and Haraldplass Deaconess Hospital for access to the MRI facility. Additional support by RCN grants 177458/V50 and 231286/F20 | (9) |
| NIMAGE | Authors | This project was supported by grants from National Institutes of Health (grant R01MH62873 to SV Faraone) for initial sample recruitment, and from NWO Large Investment (grant 1750102007010 to JK Buitelaar), NWO Brain & Cognition (grant 433-09-242 to JK Buitelaar), ZonMW Grant 60-60600-97-193, and grants from Radboud University Medical Center, University Medical Center Groningen, Accare, and VU University Amsterdam for subsequent assessment waves. NeuroIMAGE also receives funding from the European Community’s Seventh Framework Programme (FP7/2007 – 2013) under grant agreements n° 602805 (Aggressotype), n° 278948 (TACTICS), and n° 602450 (IMAGEMEND), and from the European Community’s Horizon 2020 Programme (H2020/2014 – 2020) under grant agreements n° 643051 (MiND) and n° 667302 (CoCA). | (10) |
| PING | http://pingstudy.ucsd.edu/ | Data used in the preparation of this article were obtained from the Pediatric Imaging, Neurocognition and Genetics (PING) Study database (http://ping.chd.ucsd.edu/). PING was launched in 2009 by the National Institute on Drug Abuse (NIDA) and the Eunice Kennedy Shriver National Institute Of Child Health & Human Development (NICHD) as a 2-year project of the American Recovery and Reinvestment Act. The primary goal of PING has been to create a data resource of highly standardized and carefully curated magnetic resonance imaging (MRI) data, comprehensive genotyping data, and developmental and neuropsychological assessments for a large cohort of developing children aged 3 to 20 years. The scientific aim of the project is, by openly sharing these data, to amplify the power and productivity of investigations of healthy and disordered development in children, and to increase understanding of the origins of variation in neurobehavioral phenotypes. For up-to-date information, see http://ping.chd.ucsd.edu/."  Data collection and sharing for this project was funded by the Pediatric Imaging, Neurocognition and Genetics Study (PING) (National Institutes of Health Grant RC2DA029475). PING is funded by the National Institute on Drug Abuse and the Eunice Kennedy Shriver National Institute of Child Health & Human Development. PING data are disseminated by the PING Coordinating Center at the Center for Human Development, University of California, San Diego. | (11) |
| PNC | https://www.med.upenn.edu | Support for the collection of the data sets was provided by grant RC2MH089983 awarded to R. Gur and RC2MH089924 awarded to H. Hakonarson. Subjects were recruited through the Center for Applied Genomics at The Children’s Hospital in Philadelphia. | (12,13) |
| STROKEMRI/ MOT | Authors | Supported by the Research Council of Norway (249795, 248238), the South-Eastern Norway Regional Health Authority (2014097, 2015044, 2015073, 2016083), and the Norwegian ExtraFoundation for Health and Rehabilitation (2015/FO5146).  For STROKEMRI genotyping, we made use of the Illumina Global Screening Array-v1 chip. | (14) |
| TOP | Authors | The work was funded by the Research Council of Norway (213837, 223273, 204966/F20, 213694, 229129, 249795/F20, 248778), the South-Eastern Norway Regional Health Authority (2013-123, 2014-097, 2015-073, #2017-112) and Stiftelsen Kristian Gerhard Jebsen. | (15–18) |
| UBA | Authors | European Community‘s Seventh Framework Programme (FP7/2007–2013) grant agreement #602450 (IMAGEMEND); Swiss National Science Foundation (grants 163434, 147570 and 159740) | (19) |
| UKBB | https://www.ukbiobank.ac.uk/ | All subjects with a primary or secondary ICD-10 diagnosis with a mental or neurological disorder were excluded prior to analysis and the remaining subjects included as healthy controls. The used UK Biobank project ID number is #27412. | (20) |
| UNIBA | Authors | This work was supported by a “Capitale Umano ad Alta Qualificazione” grant by Fondazione Con Il Sud awarded to Alessandro Bertolino and by a Hoffmann-La Roche Collaboration Grant awarded to Giulio Pergola. This project has received funding from the European Union Seventh Framework Programme for research, technological development and demonstration under grant agreement no. 602450 (IMAGEMEND). This paper reflects only the author's views and the European Union is not liable for any use that may be made of the information contained therein. | (21) |

*Figure S1. Demographics distributions per sample. Figure S1A shows the distribution of age in years (on the y-axis) per cohort (indicated on the x-axis), sorted by increasing mean age. Figure S1B visualizes the mean hippocampal volume in cubic millimeter (on the y-axis) per cohort (on the x-axis).*


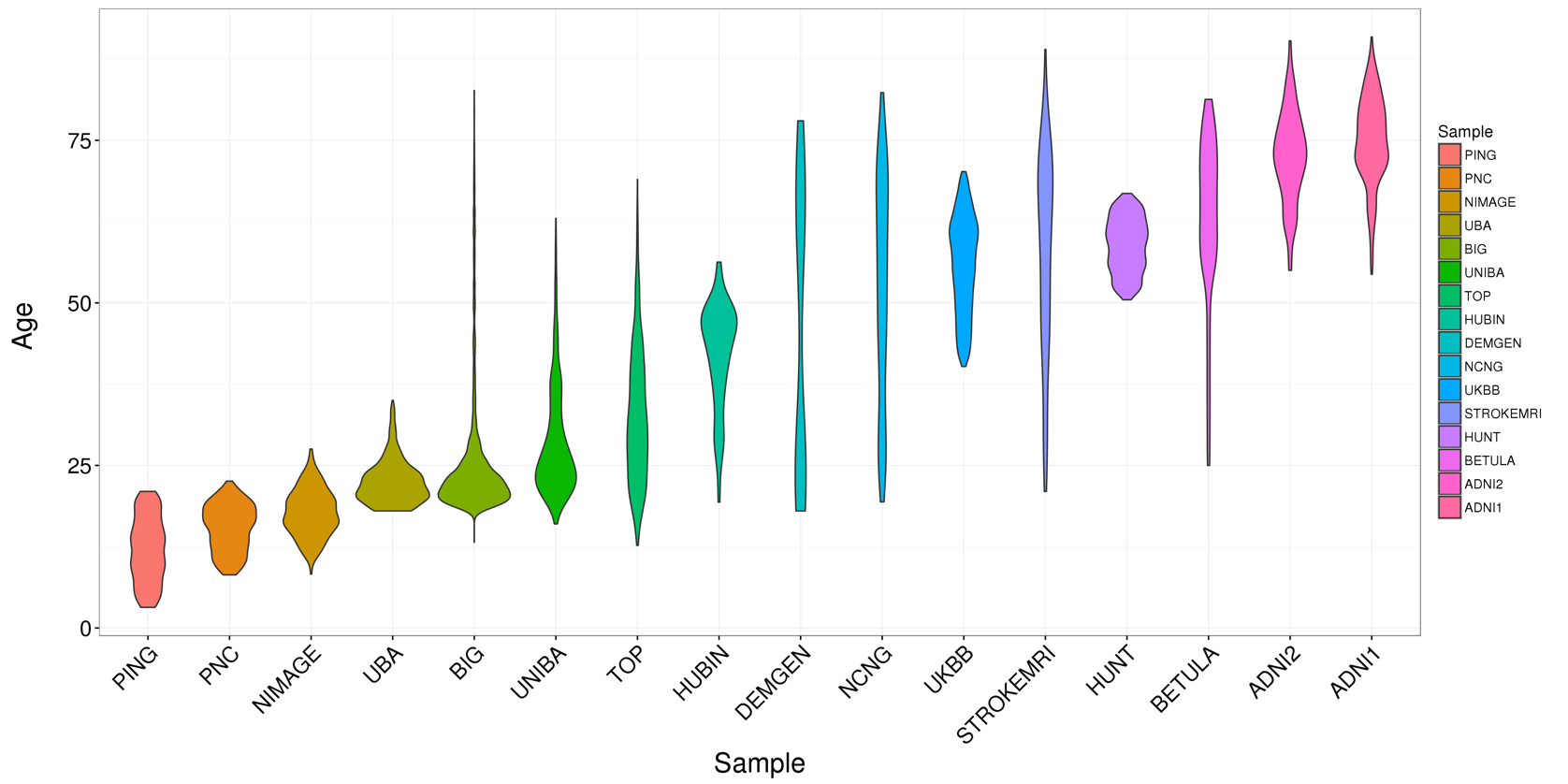
A


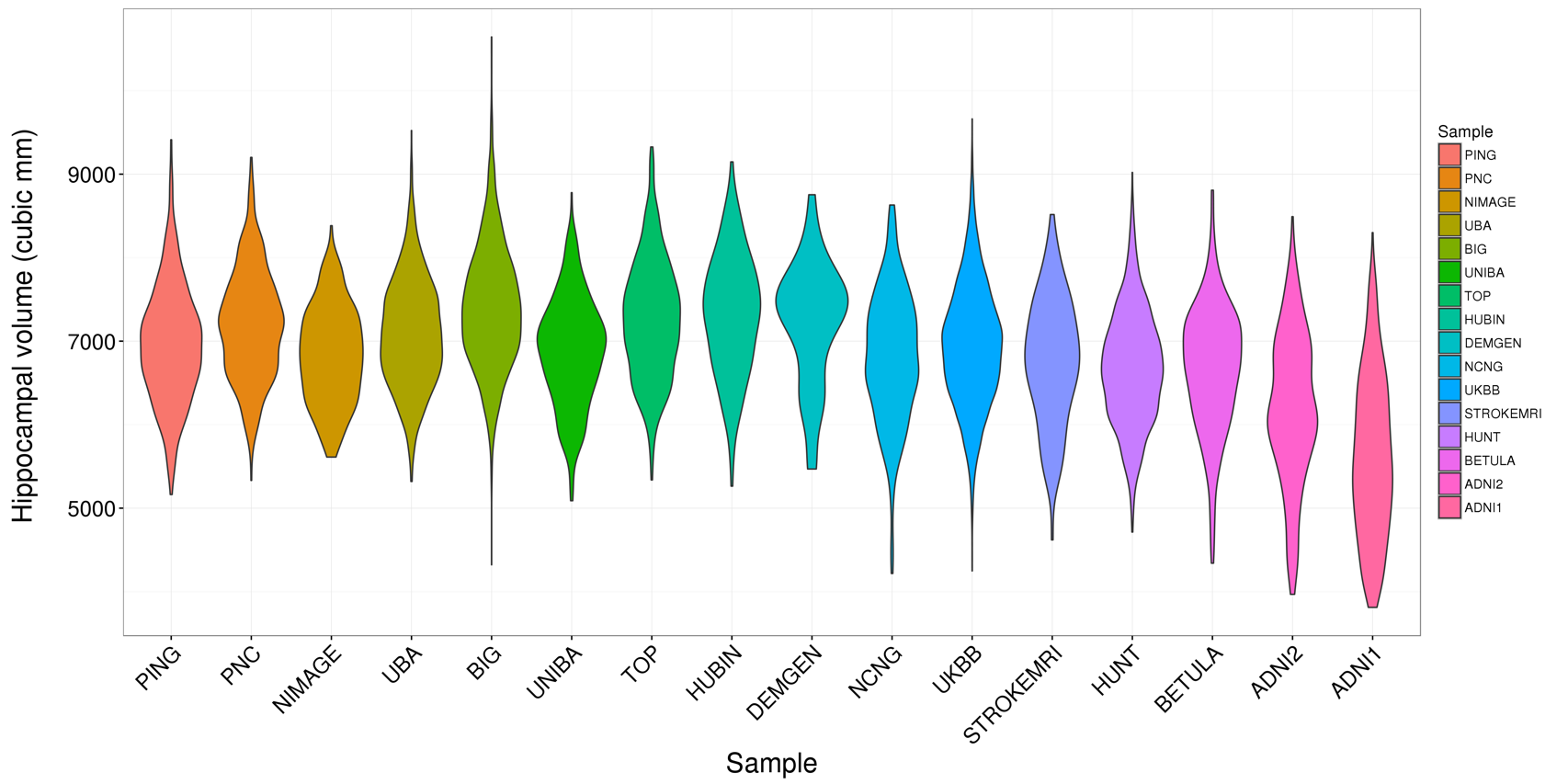
B

*Figure S2. Volume per hippocampal subfield. The y-axis shows the volume in cubic millimeter per subfield, indicated on the x-axis. The lower, middle and upper lines of the boxes indicate the 25^th^, 50^th^ (i.e. median) and 75^th^ percentile, respectively. The whiskers indicate +/- 1.5 times interquartile range above/below the quartiles.*

*
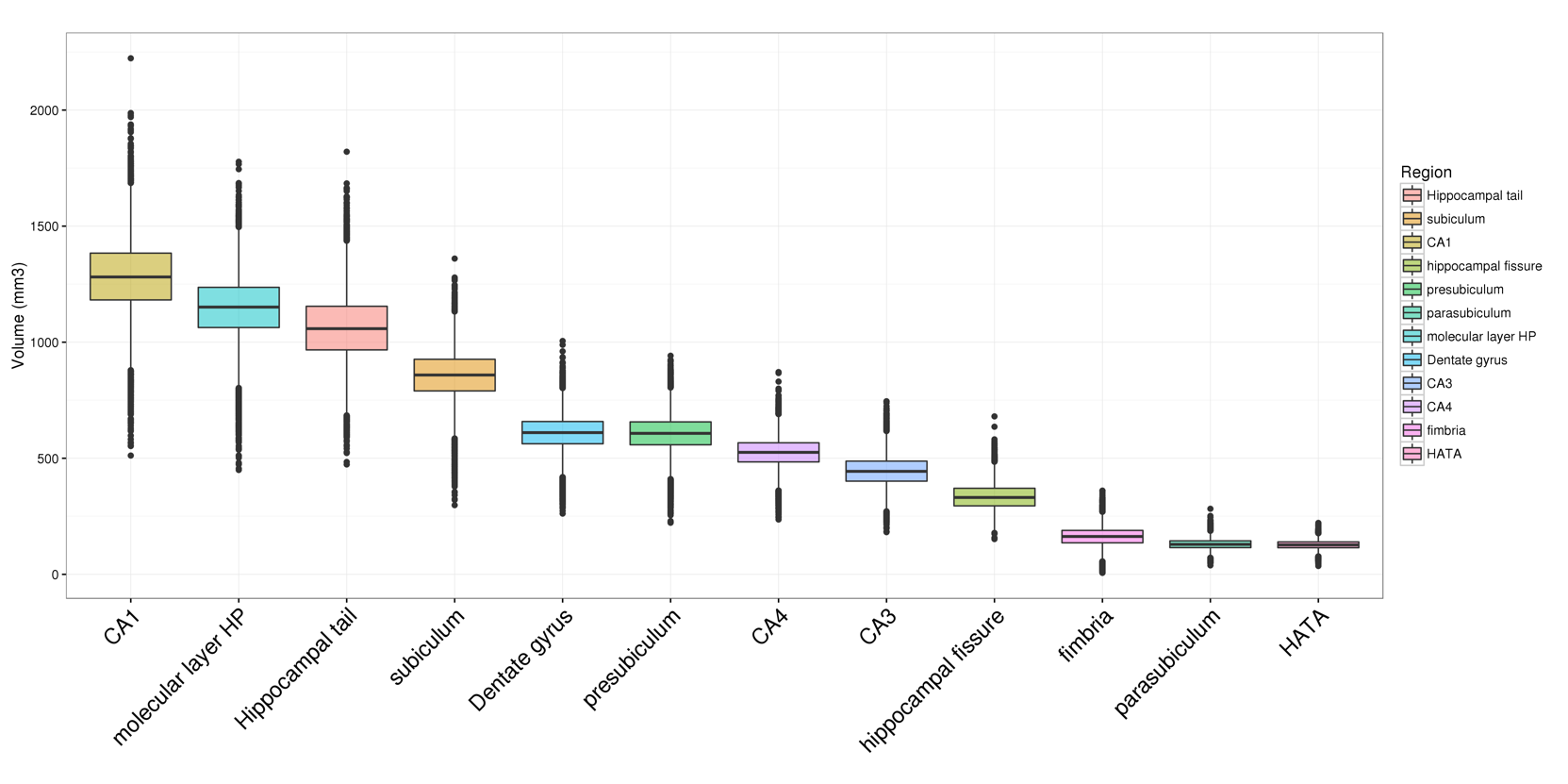
*

*Image preprocessing and quality control*

Raw data for all individuals was stored and analysed locally at the University of Oslo, where we deployed a harmonized analysis protocol applied to each subject’s raw data. We performed automated surface-based morphometry and subcortical segmentation using Freesurfer v5.3. Several of the samples were carefully screened by trained research personnel to identify segmentation errors, assess the quality of each subject’s brain images manually, edit segmentation where possible and to exclude bad data. However, due to the vast number of subjects it was not feasible to manually check the segmentation of all images. We therefore deployed an automated quality control protocol that excluded potential outliers based on global data quality measures (in addition to the described manual exclusions). In brief, we regressed age, age², sex and scanning site from mean cortical thickness, cortex volume, subcortical gray matter volume and from estimated total intracranial volume. Next, we z-standardized the resulting absolute of the residuals and excluded those subjects that exceeded pre-defined standard deviation (SD) thresholds. Before the analyses, we excluded individuals identified by manual QC as well as those exceeding a threshold of 4 SD on any of the regions of interest or ICV.

*Freesurfer v5.3 vs v6.0*

The vast majority of structural MRI scans available for this study had already been preprocessed with FreeSurfer v5.3. Given the large computational cost of re-processing these scans with FreeSurfer v6.0, we decided to stick with this version and run the v6.0 hippocampal subfield segmentation on top. We did perform a sanity check: First, we ran the recon-all –all stream from v6.0, followed by the v6.0 hippocampal subfield segmentation for fifty participants. We then correlated the resulting hippocampal subfield estimates with those obtained through the combination of v5.3 for the main segmentation with v6.0 for the subfield segmentation for these same fifty subjects. The resulting correlation per subfield is shown below.

| Subfield | Correlation |
| --- | --- |
| Hippocampal tail | 0.96 |
| Subiculum | 0.95 |
| CA1 | 0.93 |
| Hippocampal fissure | 0.94 |
| Presubiculum | 0.94 |
| Parasubiculum | 0.87 |
| Molecular layer | 0.93 |
| Dentate gyrus | 0.91 |
| CA3 | 0.92 |
| CA4 | 0.89 |
| Fimbria | 0.89 |
| HATA | 0.93 |
| Whole hippocampus | 0.95 |

*Genotyping and quality control*

Genetic data were obtained at each site using commercially available genotyping platforms. For all cohorts except BIG and UK Biobank (UKBB), we carried out phasing and imputation in-house according to protocols in line with those applied by the ENIGMA consortium (http://enigma.ini.usc.edu). This consisted of standard pre-imputation quality controls, excluding markers exhibiting high rates of genotyping missingness (above 5%), minor allele frequency (MAF) below 1% or deviating from Hardy Weinberg equilibrium (p<1*10^-6^). Individuals exhibiting high rates of genotyping missingness (above 5%), cryptic relatedness (pi-hat above 18.5%) or genome-wide heterozygosity (outside mean ±4 SD of the sample) were removed from the analyses. We restricted our analyses to those with European ancestry as determined through multidimensional scaling (MDS). MACH (http://www.sph.umich.edu/csg/abecasis/MACH) was used to impute the genotypes onto the reference haplotypes from the 1000 Genomes Project (build 37, assembly hg19). After imputation, genetic data were further quality checked to remove poorly imputed SNPs (estimated R^2^<0.3) and those with low MAF (<5%) or failing HWE at 1x10^-6^. For UKBB and BIG, we used the provided imputed data, which were processed with established protocols.^24,25^ We further carried out the same post-imputation QC steps as described for the other samples.

*Table S3. Genome-wide complex trait analysis heritability estimates, with full test statistics.*

| *ROI* | *H2* | *SE* | *Pval* |
| --- | --- | --- | --- |
| Hippocampal tail | 0.27 | 0.02 | 1.00e-16 |
| Subiculum | 0.22 | 0.02 | 1.00e-16 |
| CA1 | 0.22 | 0.02 | 1.00e-16 |
| Hippocampal fissure | 0.20 | 0.02 | 1.00e-16 |
| Presubiculum | 0.21 | 0.02 | 1.00e-16 |
| Parasubiculum | 0.14 | 0.02 | 1.00e-16 |
| Molecular layer HP | 0.21 | 0.02 | 1.00e-16 |
| Dentate gyrus | 0.22 | 0.02 | 1.00e-16 |
| CA3 | 0.24 | 0.02 | 1.00e-16 |
| CA4 | 0.22 | 0.02 | 1.00e-16 |
| Fimbria | 0.17 | 0.02 | 1.00e-16 |
| HATA | 0.17 | 0.02 | 1.00e-16 |
| Whole hippocampus | 0.23 | 0.02 | 1.00e-16 |
| Lateral ventricle | 0.16 | 0.02 | 1.00e-16 |
| Cerebellum | 0.25 | 0.02 | 1.00e-16 |
| Thalamus | 0.20 | 0.02 | 1.00e-16 |
| Caudate | 0.25 | 0.02 | 1.00e-16 |
| Putamen | 0.22 | 0.02 | 1.00e-16 |
| Pallidum | 0.16 | 0.02 | 1.00e-16 |
| Brain stem | 0.27 | 0.02 | 1.00e-16 |
| Amygdala | 0.17 | 0.02 | 1.00e-16 |
| Accumbens | 0.17 | 0.02 | 1.00e-16 |
| Frontal | 0.18 | 0.02 | 1.00e-16 |
| Parietal | 0.18 | 0.02 | 1.00e-16 |
| Temporal | 0.20 | 0.02 | 1.00e-16 |
| Occipital | 0.25 | 0.02 | 1.00e-16 |
| Cingulate | 0.15 | 0.02 | 1.00e-16 |
| Insular | 0.22 | 0.02 | 1.00e-16 |

*ROI=Region of interest, H2=Heritability, SE=Standard error, Pval=P-value*

Table S4. Whole genome significant loci for the subfields without covarying for total hippocampal volume

| **Structure** | **Locus** | **Lead SNP** | **A1** | **Chr** | **Position (BP)** | **Beta**† | **P-value** | **Mapped gene(s)** |
| --- | --- | --- | --- | --- | --- | --- | --- | --- |
| Presubiculum | 1 | rs7137149 | C | 12 | 117321662 | 6.78 | 1.435E-11 | *HRK* |
| Subiculum | 1 | rs2909455 | T | 2 | 162843078 | 5.4 | 5.928E-11 | *SLC4A10, DPP4* |
|  | 2 | rs4836726 | G | 9 | 119246425 | 5.65 | 2.975E-11 | *ASTN2* |
|  | 3 | rs11611334 | G | 12 | 65766282 | -6.97 | 1.687E-13 | *WIF1, LEMD3, MSRB3* |
|  | 4 | rs77956314 | C | 12 | 117323367 | 12.96 | 9.723E-18 | *RNFT2, HRK, FBXW8* |
| CA1 | 1 | rs28411166 | T | 10 | 126552236 | 8.44 | 1.028E-09 | *FAM53B, METTL10, FAM175B* |
|  | 2 | rs17178006 | G | 12 | 65718299 | -21.43 | 5.881E-28 | *WIF1, LEMD3, MSRB3* |
|  | 3 | rs77956314 | C | 12 | 117323367 | 24.08 | 7.404E-28 | *RNFT2, HRK, FBXW8, TESC* |
| CA3 | 1 | rs2629542 | G | 10 | 126552395 | 4.06 | 2.516E-12 | *FAM53B, METTL10, FAM175B* |
|  | 2 | rs17178006 | G | 12 | 65718299 | -7.76 | 8.928E-19 | *WIF1, LEMD3, MSRB3* |
|  | 3 | rs77956314 | C | 12 | 117323367 | 7.79 | 2.182E-15 | *RNFT2, HRK, FBXW8* |
| CA4 | 1 | rs6478241 | A | 9 | 119252629 | -3.18 | 4.238E-10 | *ASTN2* |
|  | 2 | rs12218858 | C | 10 | 126474200 | 3.87 | 6.469E-15 | *FAM53B, METTL10, FAM175B* |
|  | 3 | rs1419859 | T | 12 | 4007898 | -3.27 | 7.213E-10 | NA |
|  | 4 | rs17178006 | G | 12 | 65718299 | -7.33 | 2.595E-19 | *WIF1, LEMD3, MSRB3* |
|  | 5 | rs77956314 | C | 12 | 117323367 | 9.26 | 2.182E-24 | *RNFT2, HRK, FBXW8, TESC* |
| Granule cell layer DG | 1 | rs6478241 | A | 9 | 119252629 | -3.8 | 9.97E-11 | *ASTN2* |
|  | 2 | rs12218858 | C | 10 | 126474200 | 4.45 | 8.754E-15 | *FAM53B, METTL10, FAM175B* |
|  | 3 | rs1419859 | T | 12 | 4007898 | -3.86 | 2.814E-10 | NA |
|  | 4 | rs17178006 | G | 12 | 65718299 | -8.84 | 5.254E-21 | *WIF1, LEMD3, MSRB3* |
|  | 5 | rs77956314 | C | 12 | 117323367 | 11.01 | 8.803E-26 | *RNFT2, HRK, FBXW8, TESC* |
| HATA | 1 | rs77956314 | C | 12 | 117323367 | 2.04 | 4.254E-13 | *HRK, FBXW8* |
| Molecular layer | 1 | rs1861979 | T | 2 | 162845565 | 6.42 | 2.04E-10 | *SLC4A10, DPP4* |
|  | 2 | rs28411166 | T | 10 | 126552236 | 7.32 | 2.454E-10 | *FAM53B, METTL10, FAM175B* |
|  | 3 | rs1419859 | T | 12 | 4007898 | -6.47 | 1.894E-09 | NA |
|  | 4 | rs11611334 | G | 12 | 65766282 | -9.66 | 6.201E-17 | *WIF1, LEMD3, MSRB3* |
|  | 5 | rs77956314 | C | 12 | 117323367 | 19.27 | 1.586E-25 | *RNFT2, HRK, FBXW8, TESC* |
| Fissure | 1 | rs17178006 | G | 12 | 65718299 | -5.65 | 2.474E-13 | *WIF1, LEMD3, MSRB3* |
| Hippocampal tail | 1 | rs1861979 | T | 2 | 162845565 | 11.92 | 8.576E-22 | *SLC4A10, DPP4* |
|  | 2 | rs7666515 | C | 4 | 184973306 | -7.3 | 5.995E-09 | NA |
|  | 3 | rs57246240 | A | 5 | 66112715 | 8.35 | 9.918E-11 | *MAST4* |
|  | 4 | rs10474356 | G | 5 | 90816402 | -10.58 | 7.258E-15 | NA |
|  | 5 | rs58499557 | T | 7 | 31434693 | -12.53 | 2.981E-12 | NA |
|  | 6 | rs2363340 | C | 10 | 126461368 | 9.45 | 2.949E-14 | *LHPP, FAM53B, METTL10, FAM175B* |
|  | 7 | rs77956314 | C | 12 | 117323367 | 20.52 | 1.756E-19 | *RNFT2, HRK, FBXW8, TESC* |

† mm^3^ volume, additive effects for each copy of allele 1 (A1). Chr=Chromosome, BP=Base pair

*Table S5. Genetic correlation, calculated through linkage disequilibrum score regression (LDSC), of Alzheimer’s disease and schizophrenia diagnosis with each of the subfields, corrected for total hippocampal volume.*

| Structure | Alzheimer’s disease | | | Schizophrenia | | |
| --- | --- | --- | --- | --- | --- | --- |
|  | Rg | SE | P-value | Rg | SE | P-value |
| Whole hippocampus | -0.056 | 0.103 | 0.585 | -0.032 | 0.04 | 0.429 |
| Parasubiculum | 0.177 | 0.102 | 0.083 | -0.007 | 0.046 | 0.878 |
| Presubiculum | 0.143 | 0.098 | 0.145 | -0.001 | 0.042 | 0.977 |
| Subiculum | 0.071 | 0.111 | 0.519 | 0.063 | 0.048 | 0.187 |
| CA1 | -0.088 | 0.106 | 0.403 | 0.02 | 0.05 | 0.693 |
| CA3 | -0.081 | 0.084 | 0.334 | -0.054 | 0.038 | 0.153 |
| CA4 | -0.12 | 0.097 | 0.216 | -0.06 | 0.045 | 0.184 |
| Granule cell layer DG | -0.117 | 0.096 | 0.219 | -0.064 | 0.045 | 0.153 |
| HATA | -0.188 | 0.107 | 0.079 | -0.067 | 0.047 | 0.152 |
| Fimbria | -0.246 | 0.13 | 0.058 | -0.055 | 0.052 | 0.295 |
| Molecular layer DG | -0.086 | 0.107 | 0.421 | 0.011 | 0.052 | 0.829 |
| Hippocampal fissure | -0.149 | 0.105 | 0.153 | 0.081 | 0.052 | 0.118 |
| Hippocampal tail | 0.108 | 0.092 | 0.241 | 0.037 | 0.045 | 0.405 |

*The effects of AD-related genes in younger and older subjects*

We hypothesized that AD-related genes may influence hippocampal volume predominantly later in life, through accumulation of effects over time. We therefore analyzed the association between SNPs linked to AD and hippocampal volume in younger and older subjects.

We first selected all lead SNPs from the whole-genome significant loci reported in the stage 1 IGAP 2013 AD GWAS (Lambert *et al.* 2013, see table 2 in that manuscript). Of these, twelve were available in our data. We then performed a median split on all individuals for which we had whole-genome genetic data that were not part of the 2013 GWAS (i.e. those from the ADNI samples, leaving n=18110) leading to two groups of n=9055 in size, one younger (below 53.9 years of age) and one older (above 53.9 years). We subsequently regressed whole hippocampal volume (residualized for nuisance covariates as described in the main methods section) on each of these SNPs separately for both groups. The results are shown in Table S6.

*Table S6. Effects of AD-associated SNPs on whole hippocampal volume, split by younger and older participants.*

| SNP | Younger participants | | Older participants | |
| --- | --- | --- | --- | --- |
|  | Beta | P-value | Beta | P-value |
| rs6656401 | -6.09 | 0.56 | -8.37 | 0.46 |
| rs6733839 | -14.83 | 0.27 | -9.54 | 0.68 |
| rs9271192 | 4.93 | 0.77 | 14.51 | 0.63 |
| rs10948363 | 10.41 | 0.24 | -8.42 | 0.38 |
| rs11771145 | 6.42 | 0.44 | 5.30 | 0.55 |
| **rs28834970** | **-8.26** | **0.48** | **-85.55** | **0.005** |
| **rs9331896** | **-12.39** | **0.29** | **67.37** | **0.03** |
| rs983392 | -10.77 | 0.32 | -3.14 | 0.87 |
| rs10792832 | 12.08 | 0.14 | 7.94 | 0.38 |
| rs10498633 | 1.82 | 0.85 | -3.88 | 0.70 |
| **rs4147929** | **-14.56** | **0.16** | **-37.69** | **0.0008** |
| rs3865444 | -4.69 | 0.58 | -1.96 | 0.83 |

*Figure S3. Manhattan and QQ plots for whole hippocampus and per subfield. For the manhattan plots, the red line indicates the adjusted whole-genome significance threshold (6.5x10-9 p-value), the blue line the suggestive threshold (1x10-5).*

*Whole hippocampus*

*
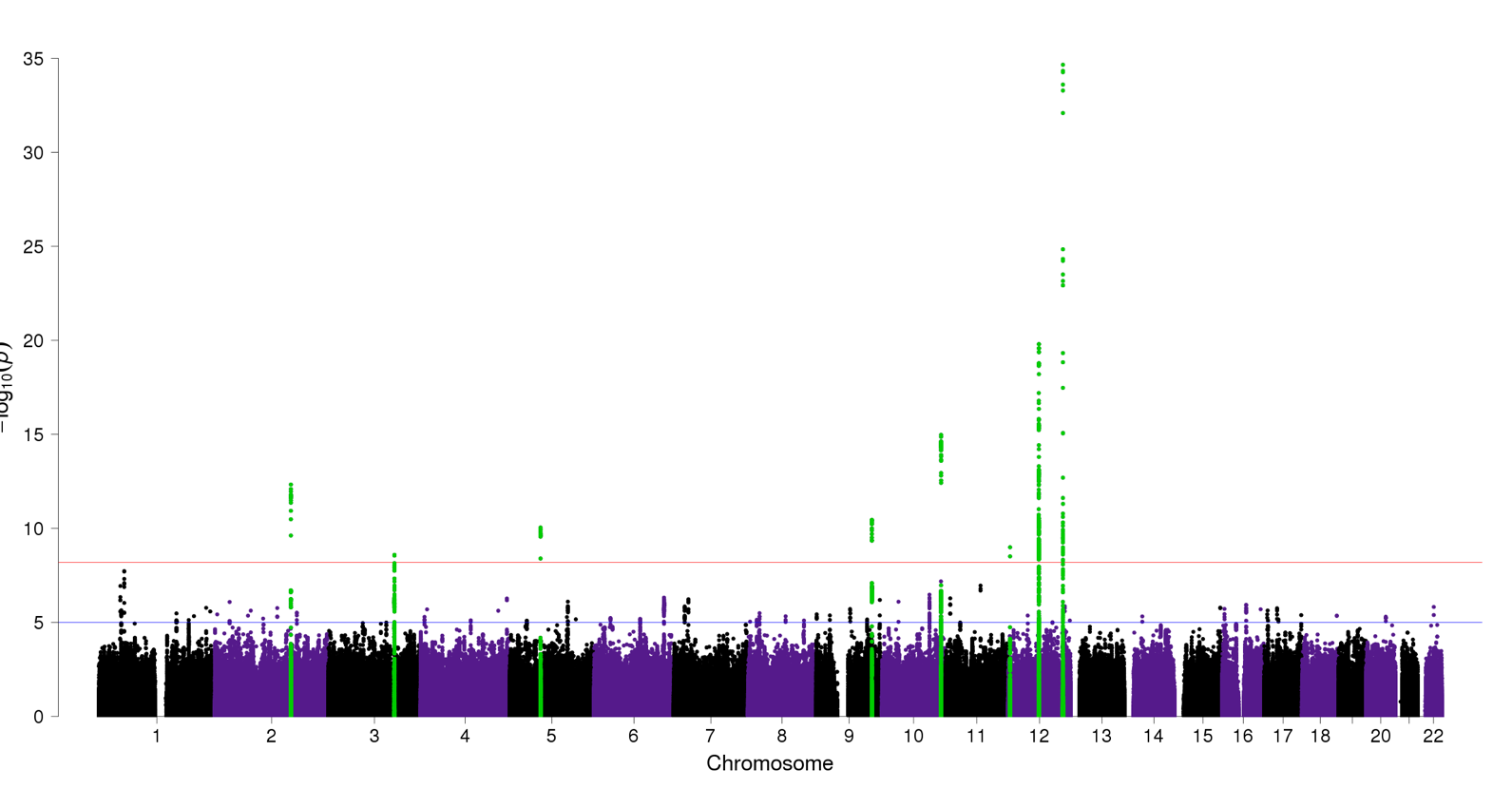
*

*
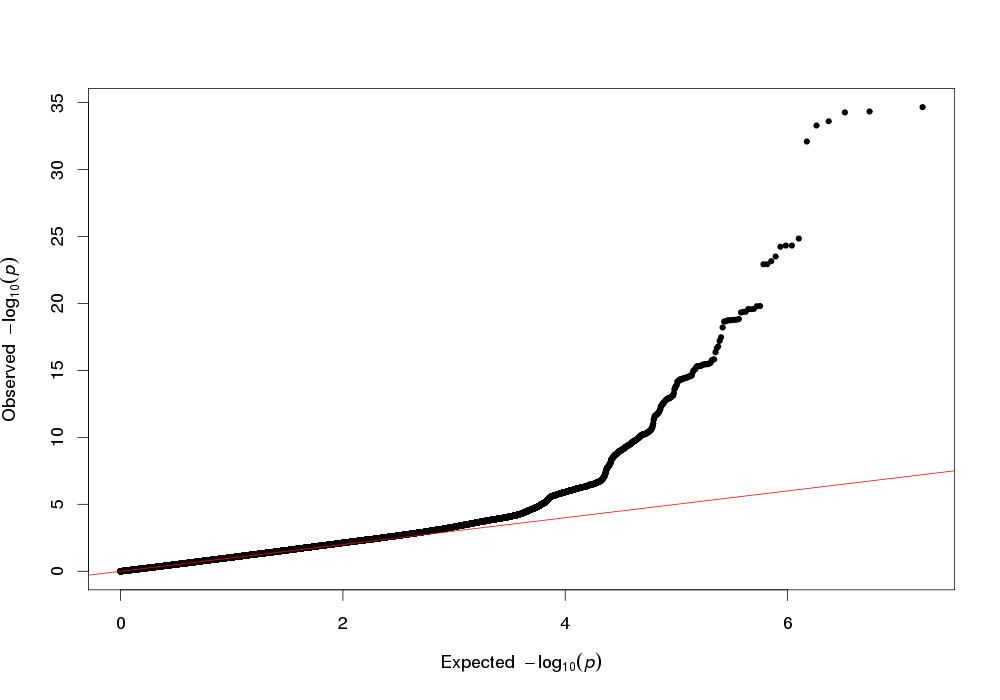
*

*CA1 corrected for total hippocampal volume*

*
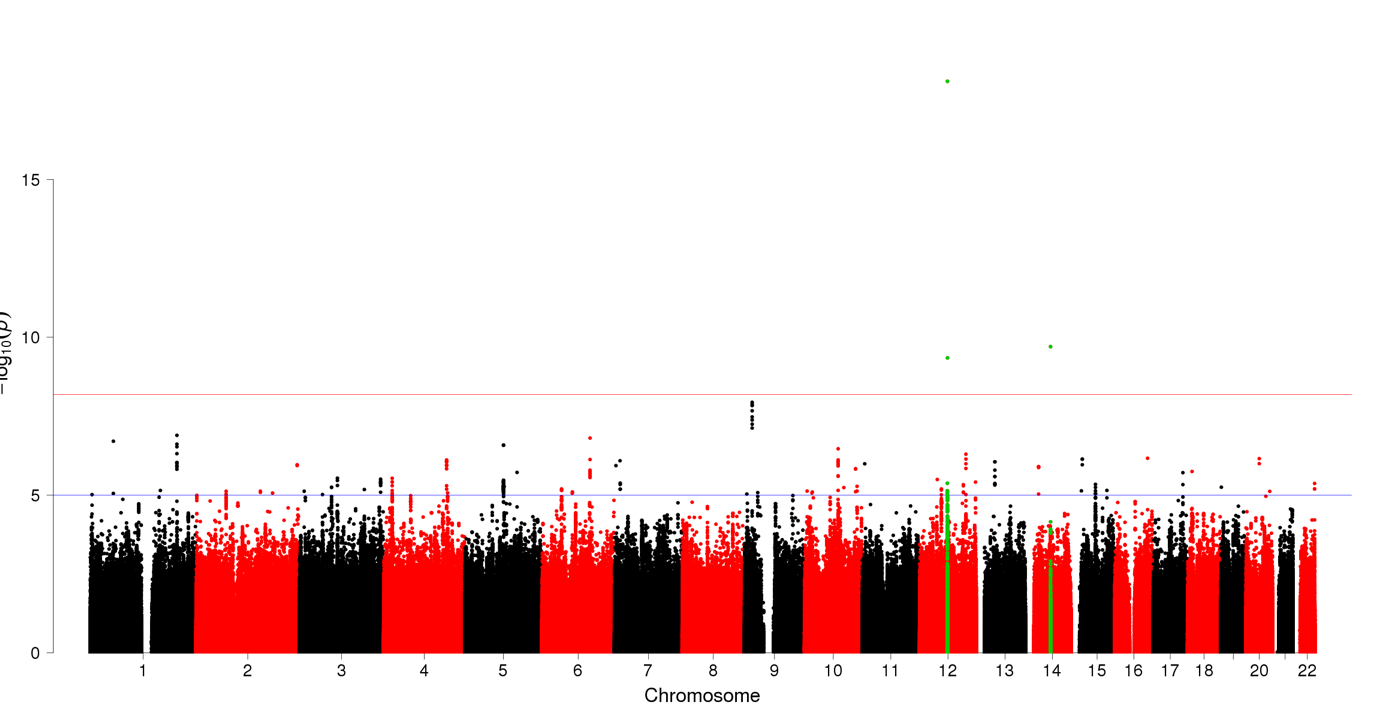

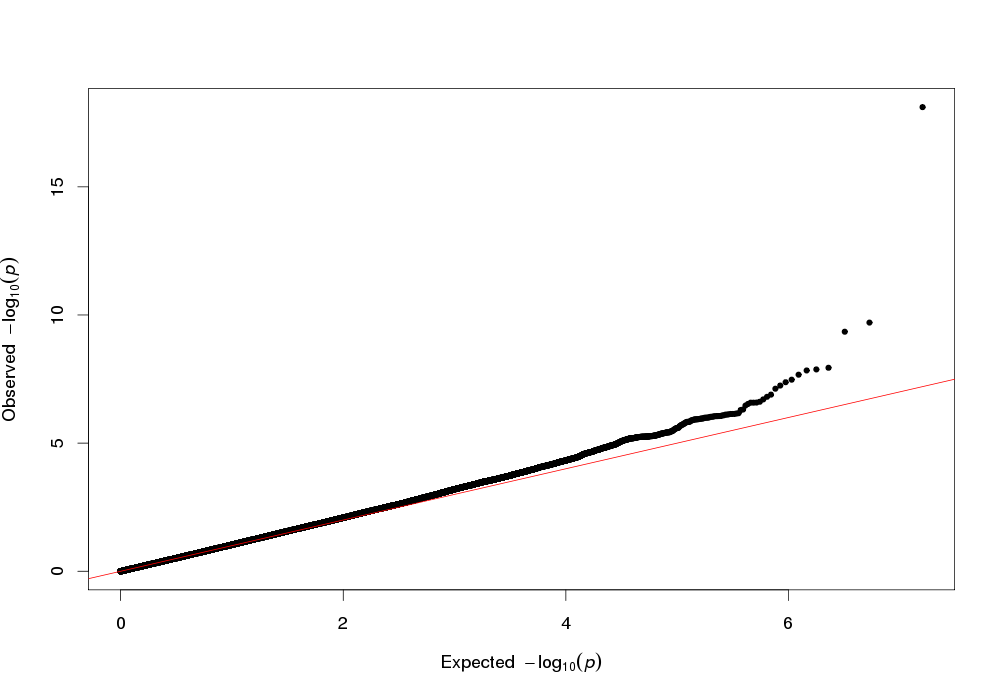
*

*CA1 uncorrected for total hippocampal volume*

*
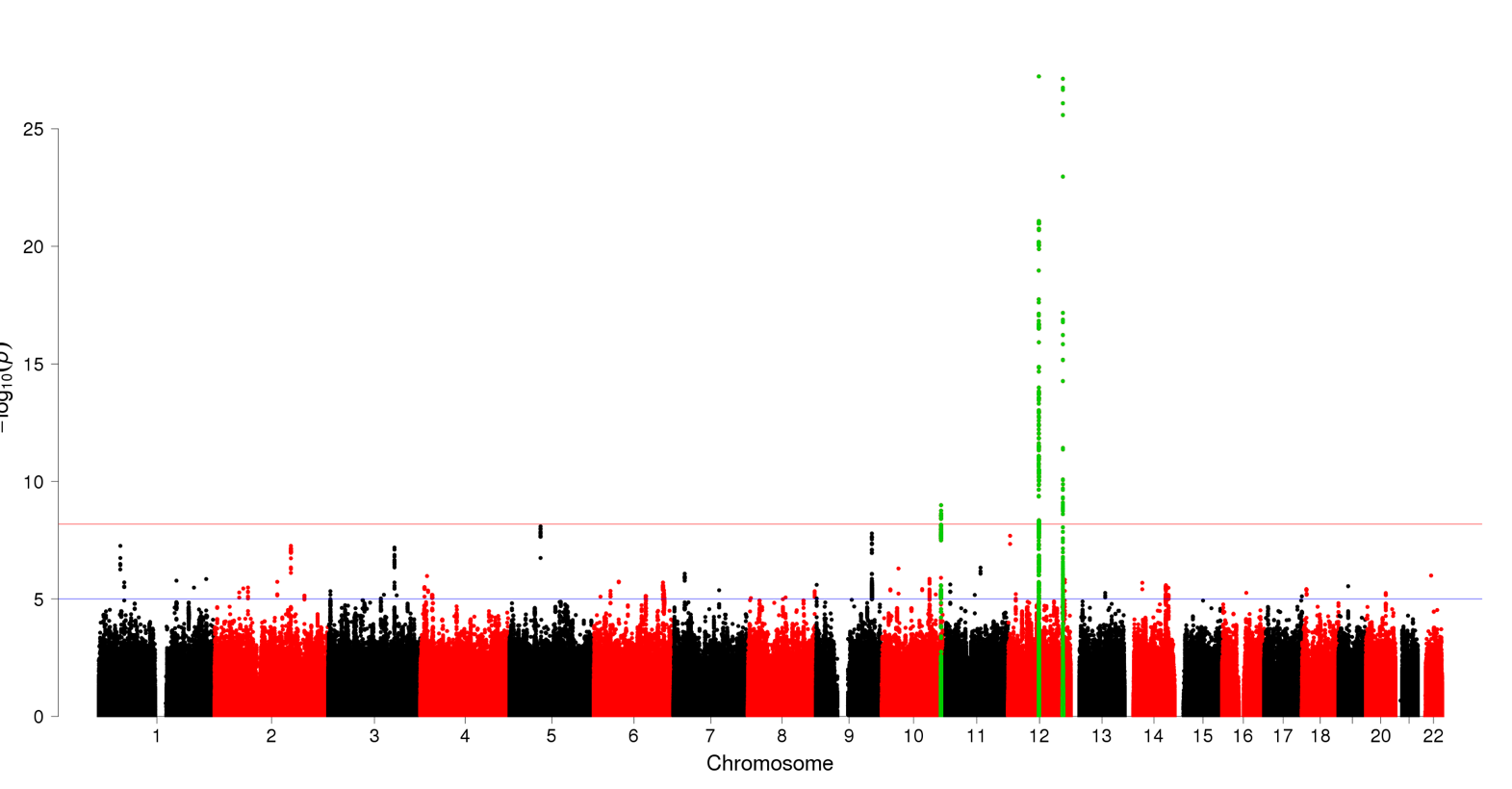
*

*
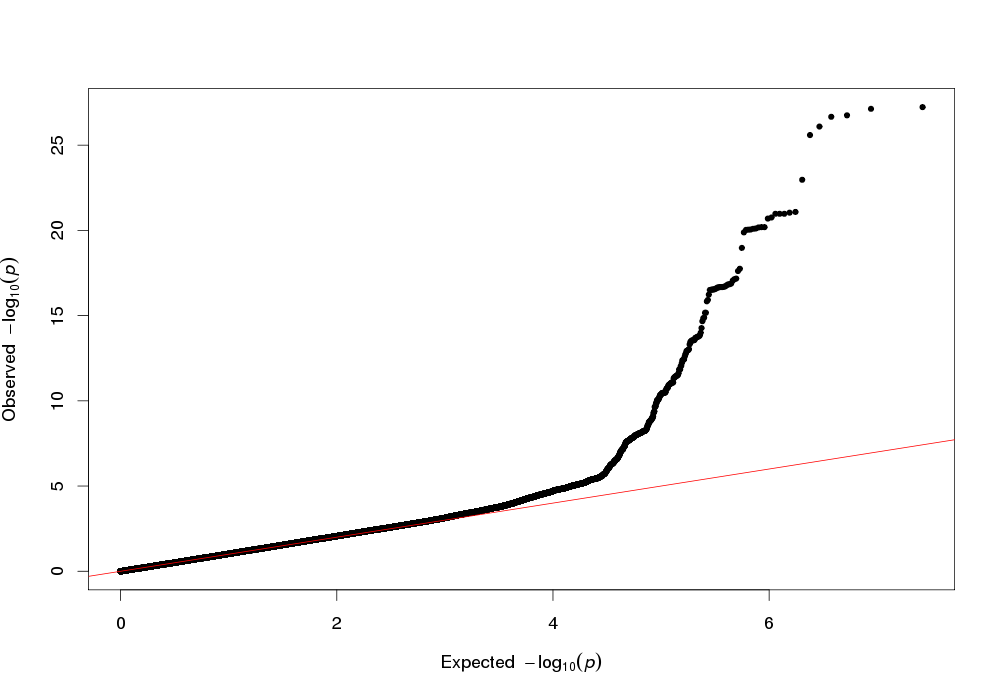
*

*CA3 corrected for total hippocampal volume*

*
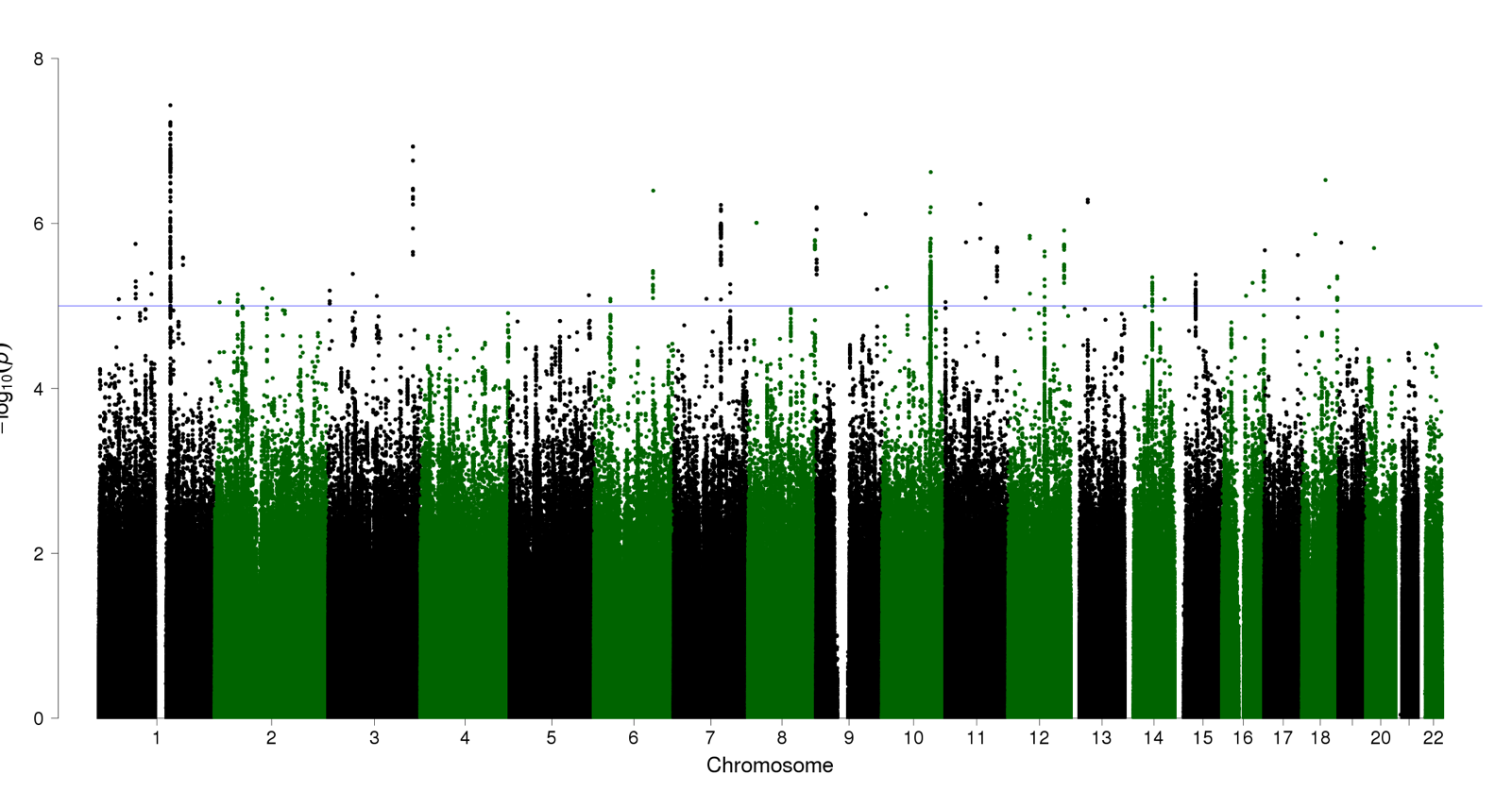

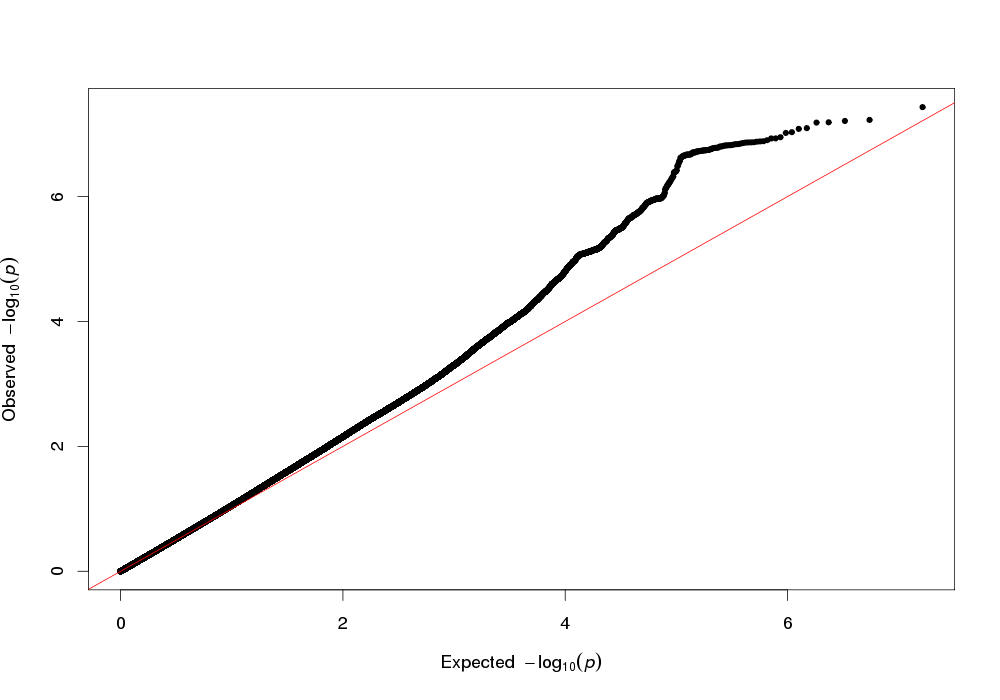
*

*CA3 uncorrected for total hippocampal volume*

*
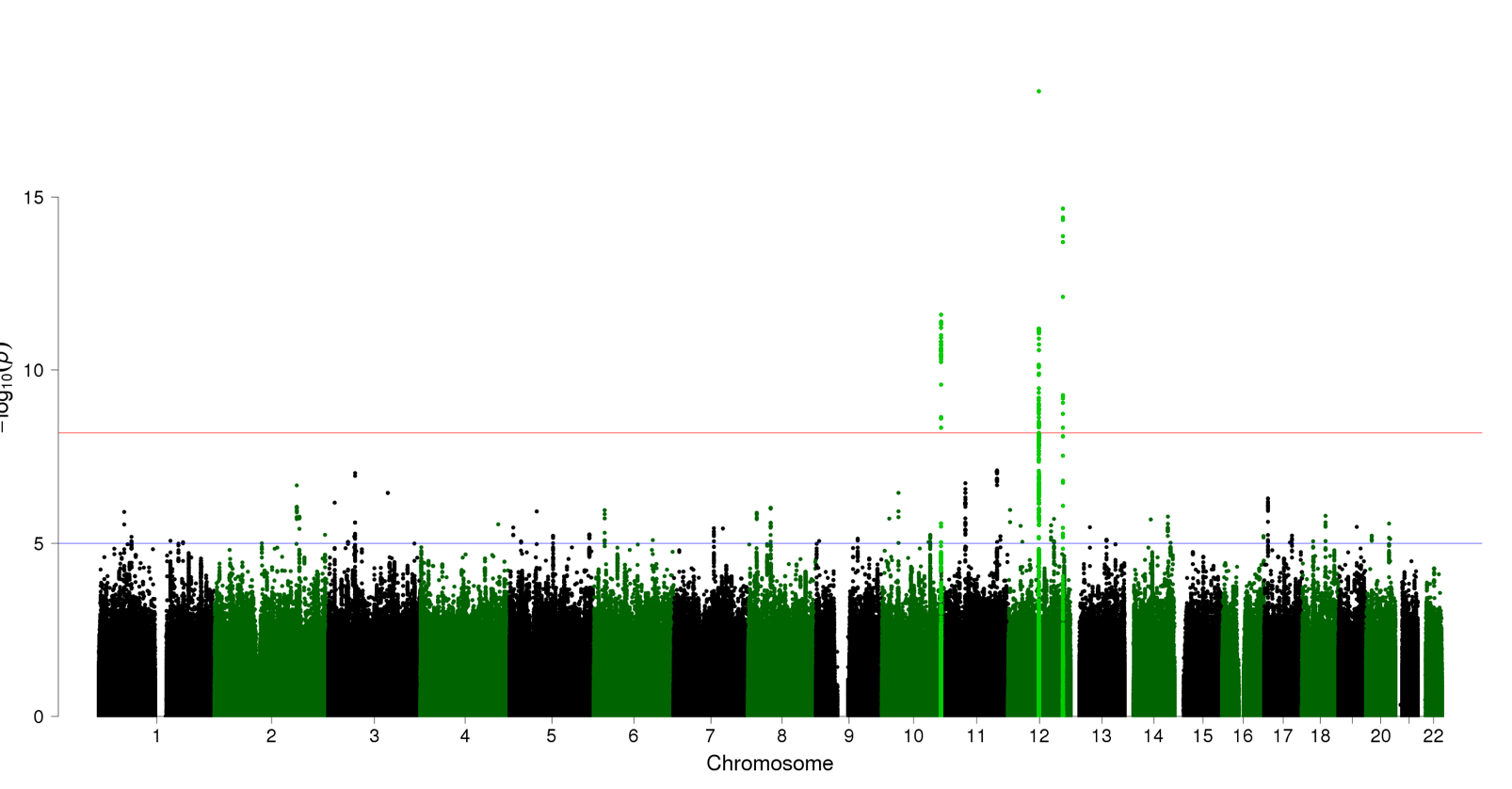

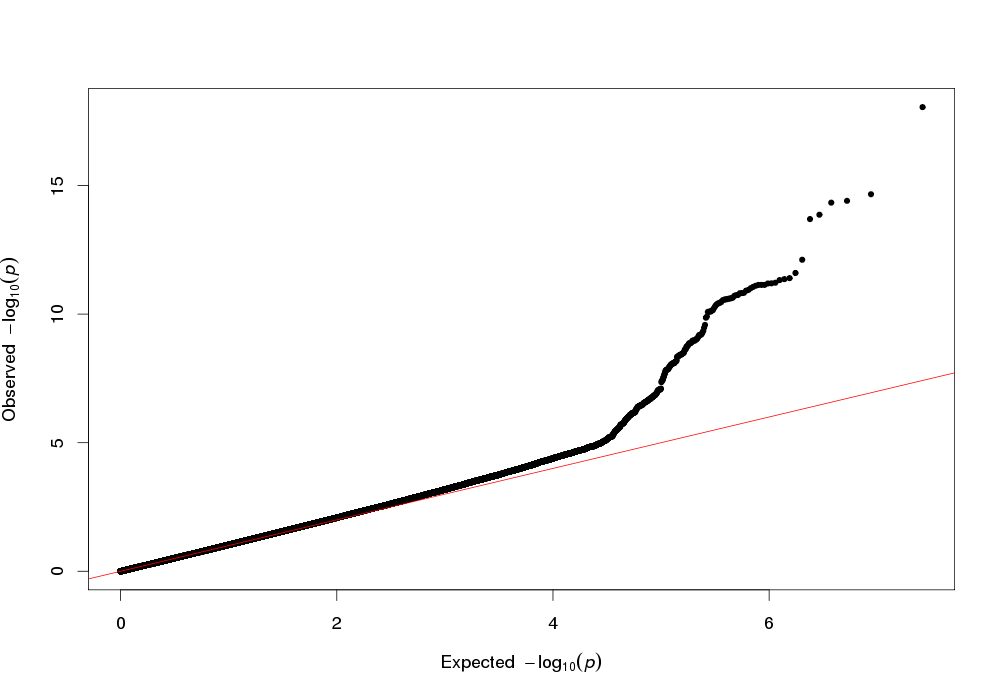
*

*CA4 corrected for total hippocampal volume*

*
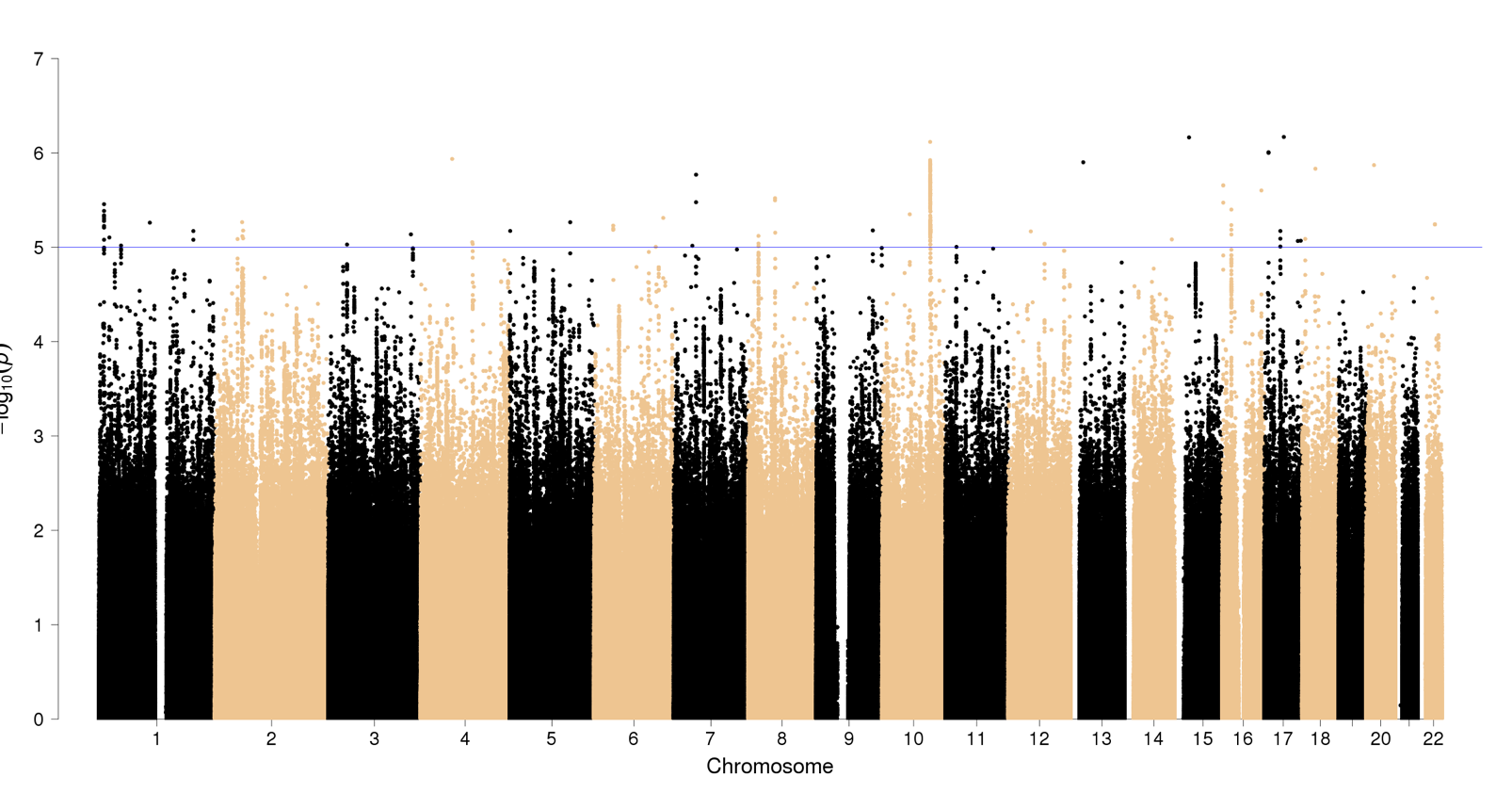

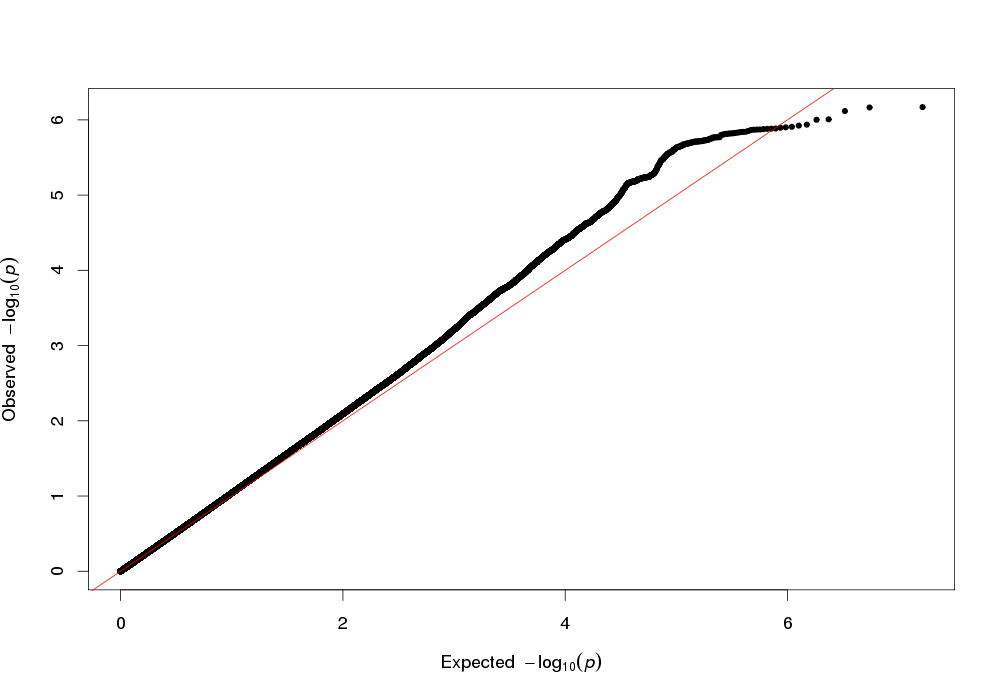
*

*CA4 uncorrected for total hippocampal volume*

*
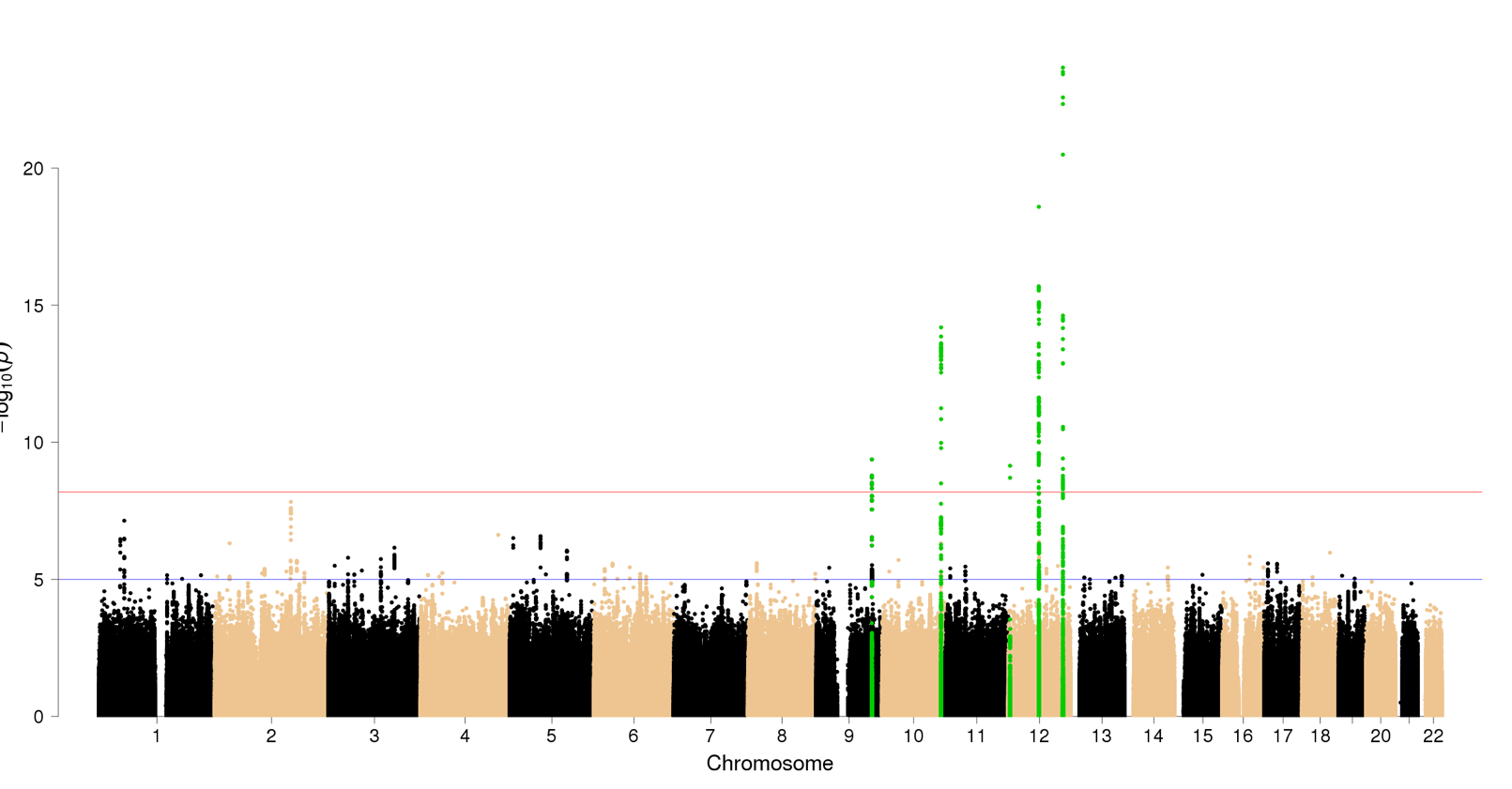

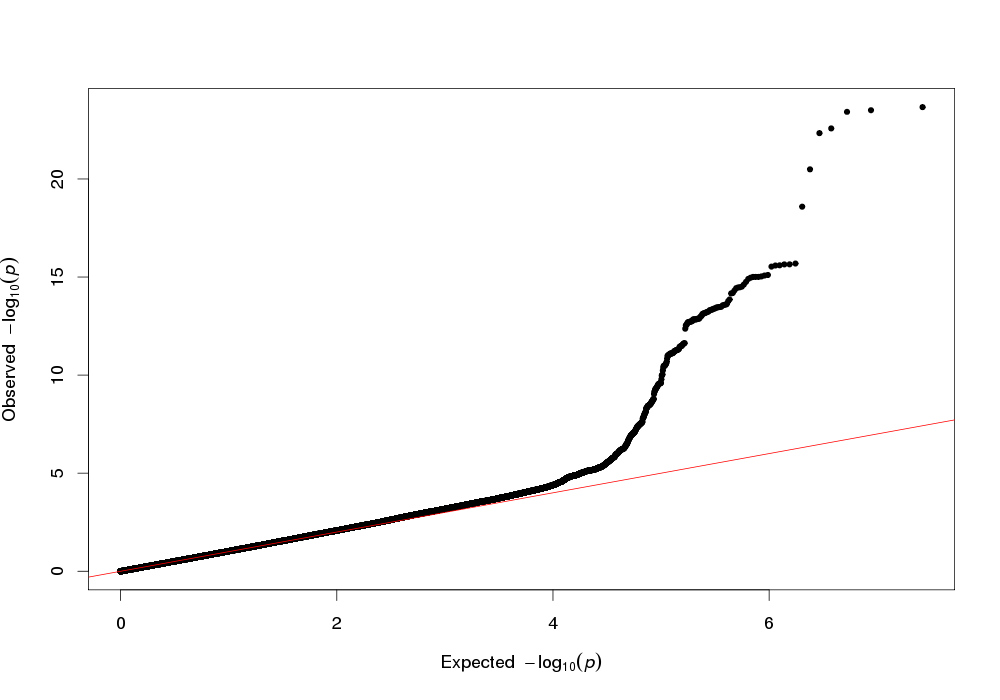
*

*Fimbria corrected for total hippocampal volume*

*
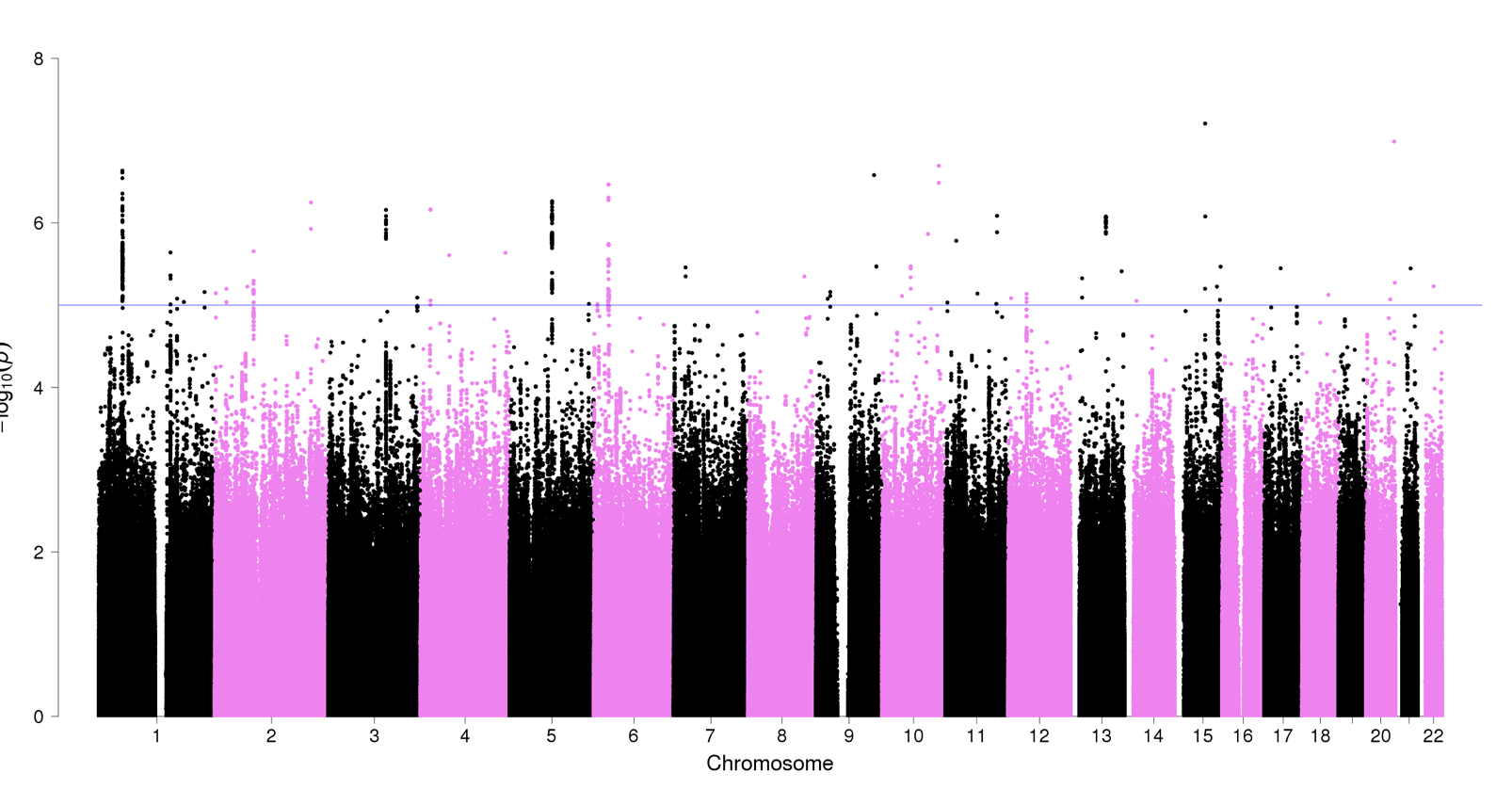

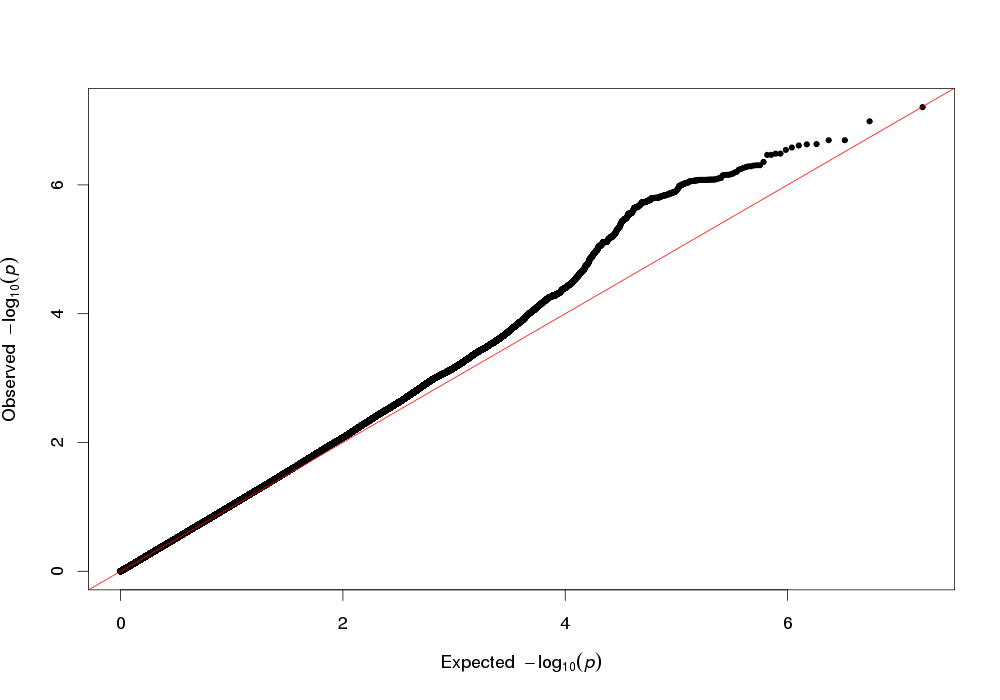
*

*Fimbria uncorrected for total hippocampal volume*

*
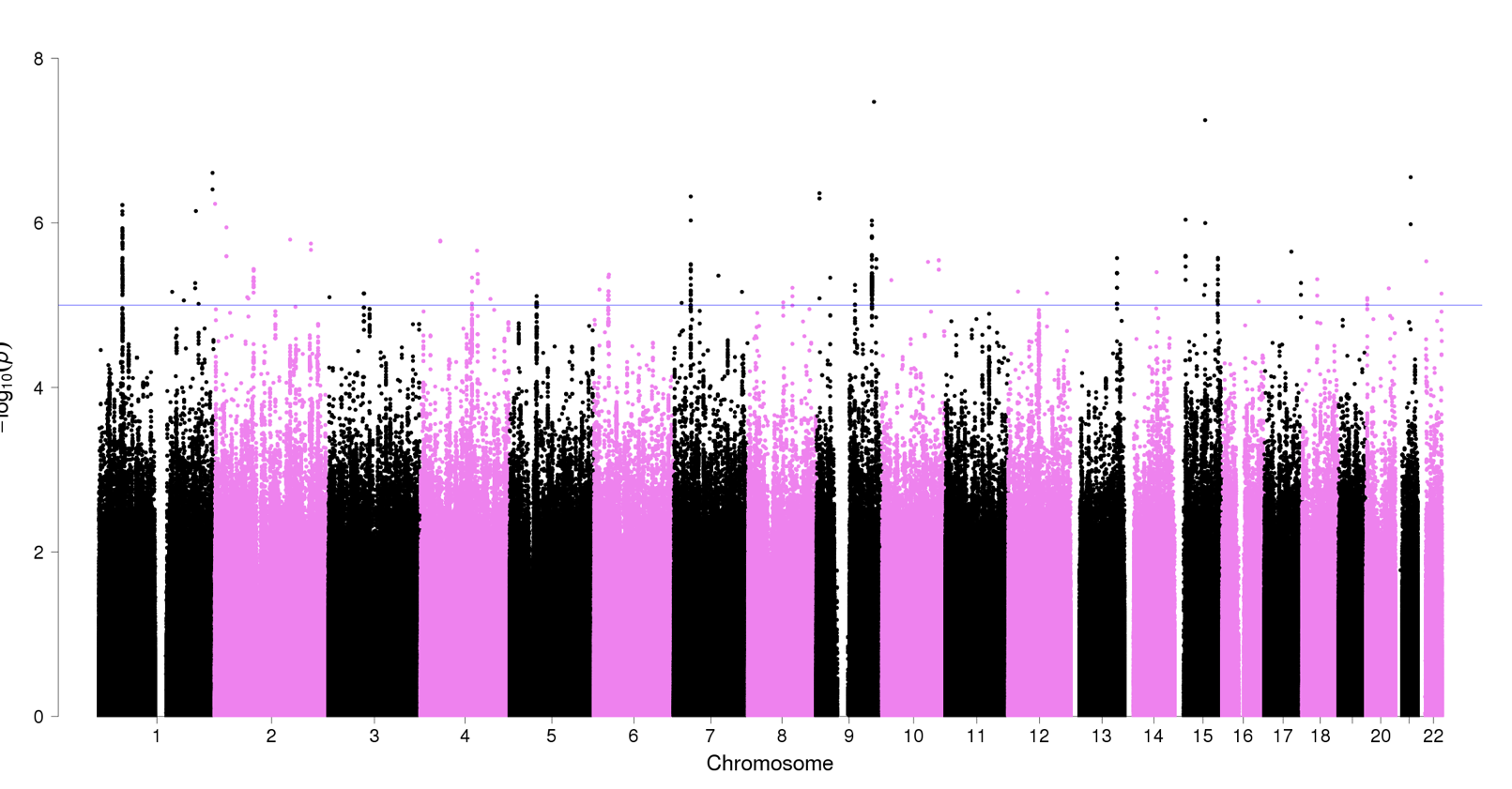

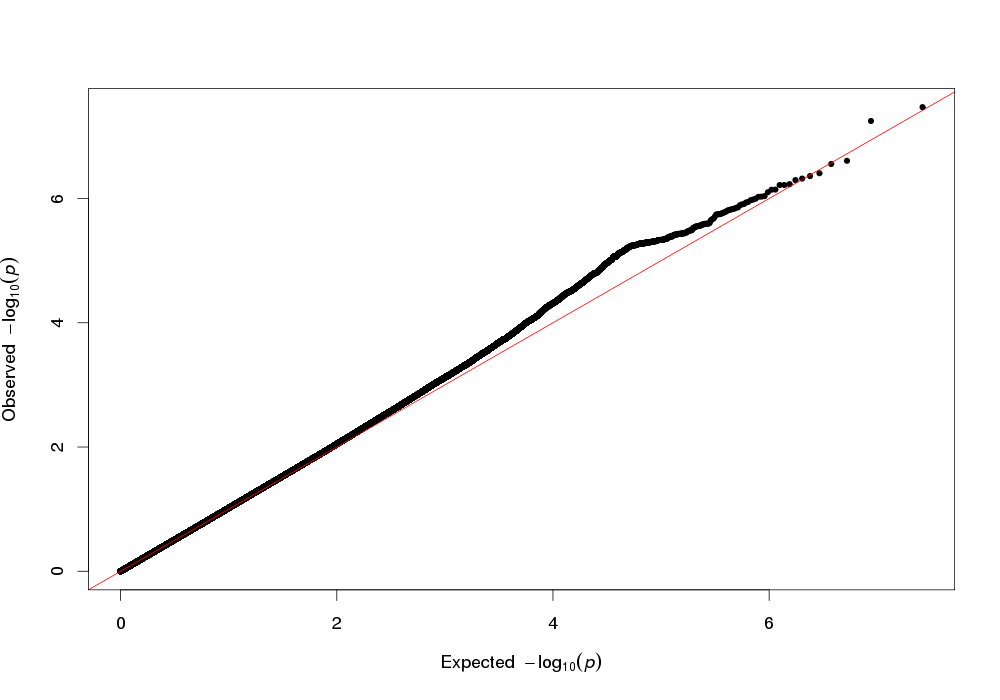
*

*Granule Cell Layer Dentate Gyrus corrected for total hippocampal volume*

*
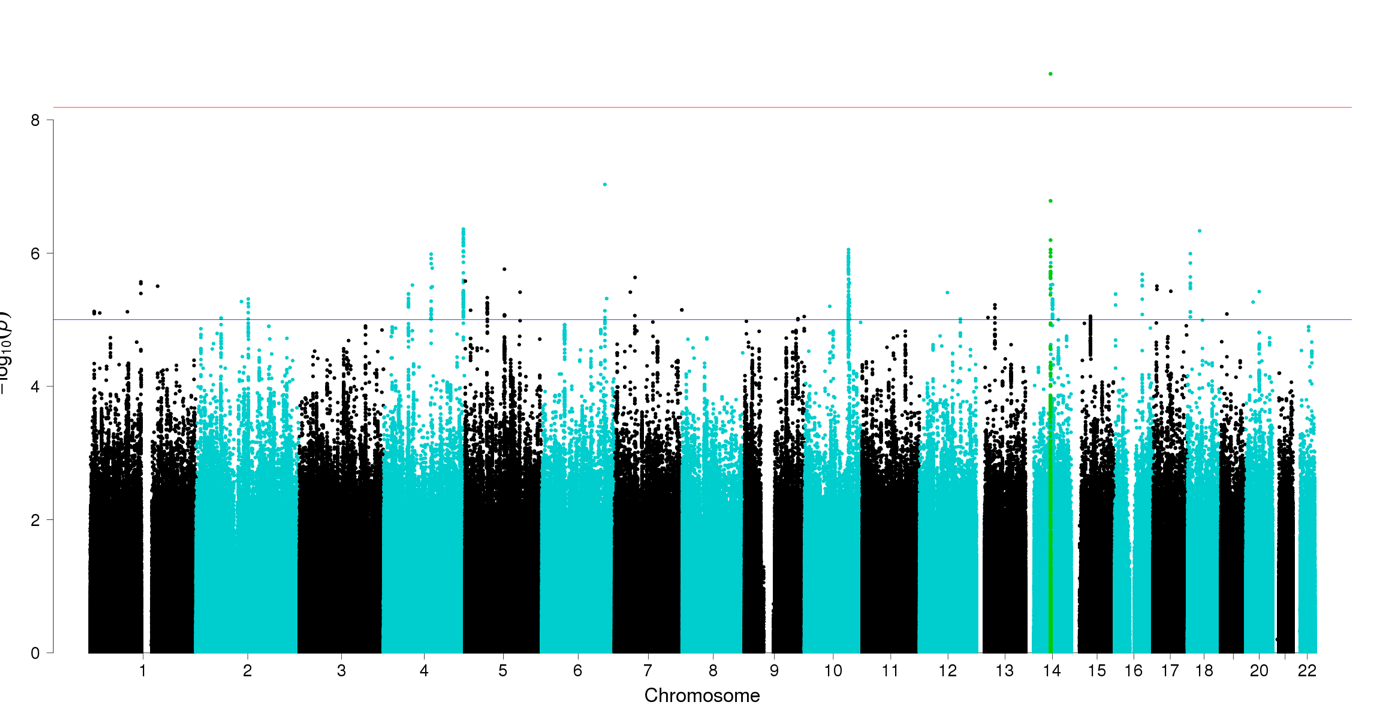

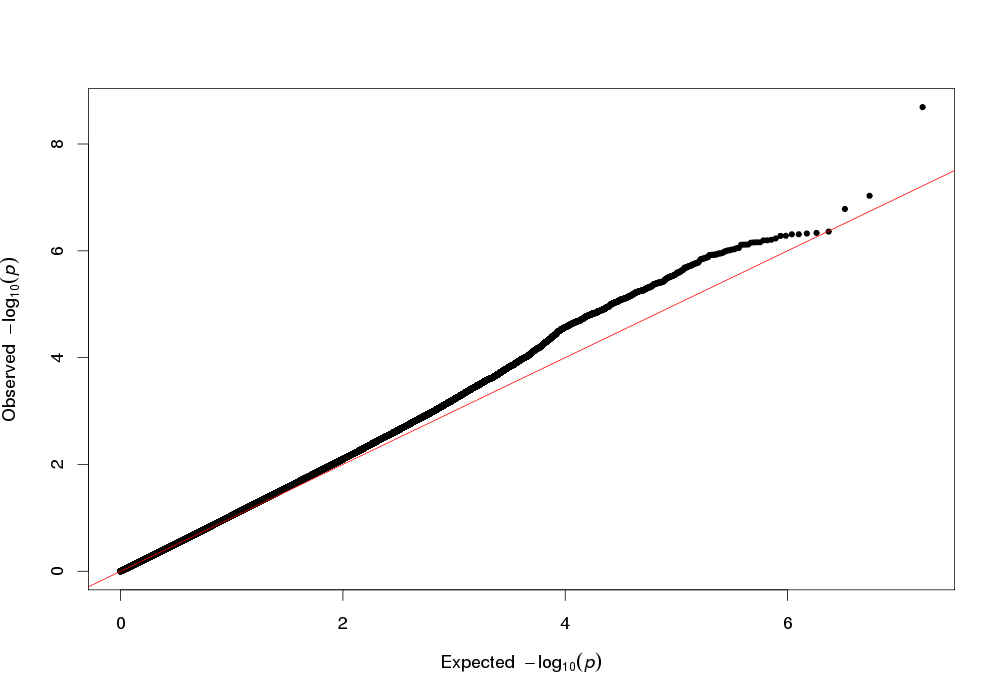
*

*Granule Cell Layer Dentate Gyrus uncorrected for total hippocampal volume*

*
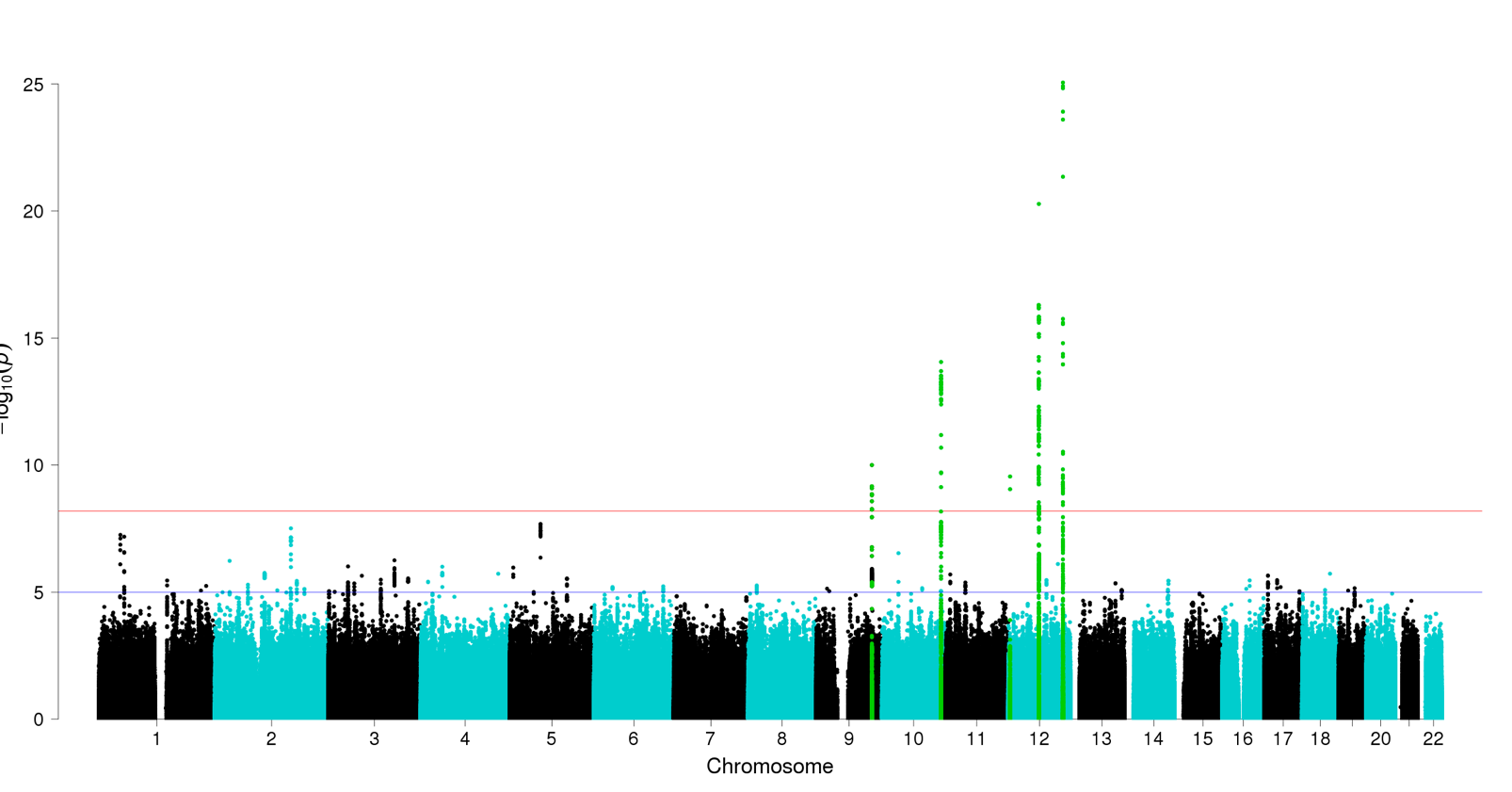

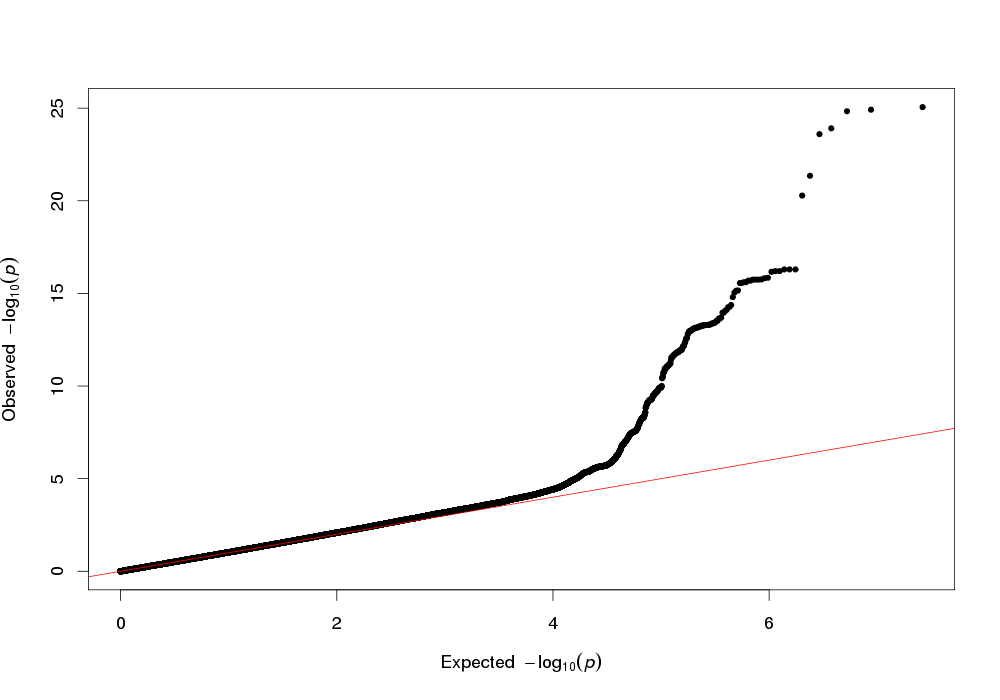
*

*HATA corrected for total hippocampal volume*

*
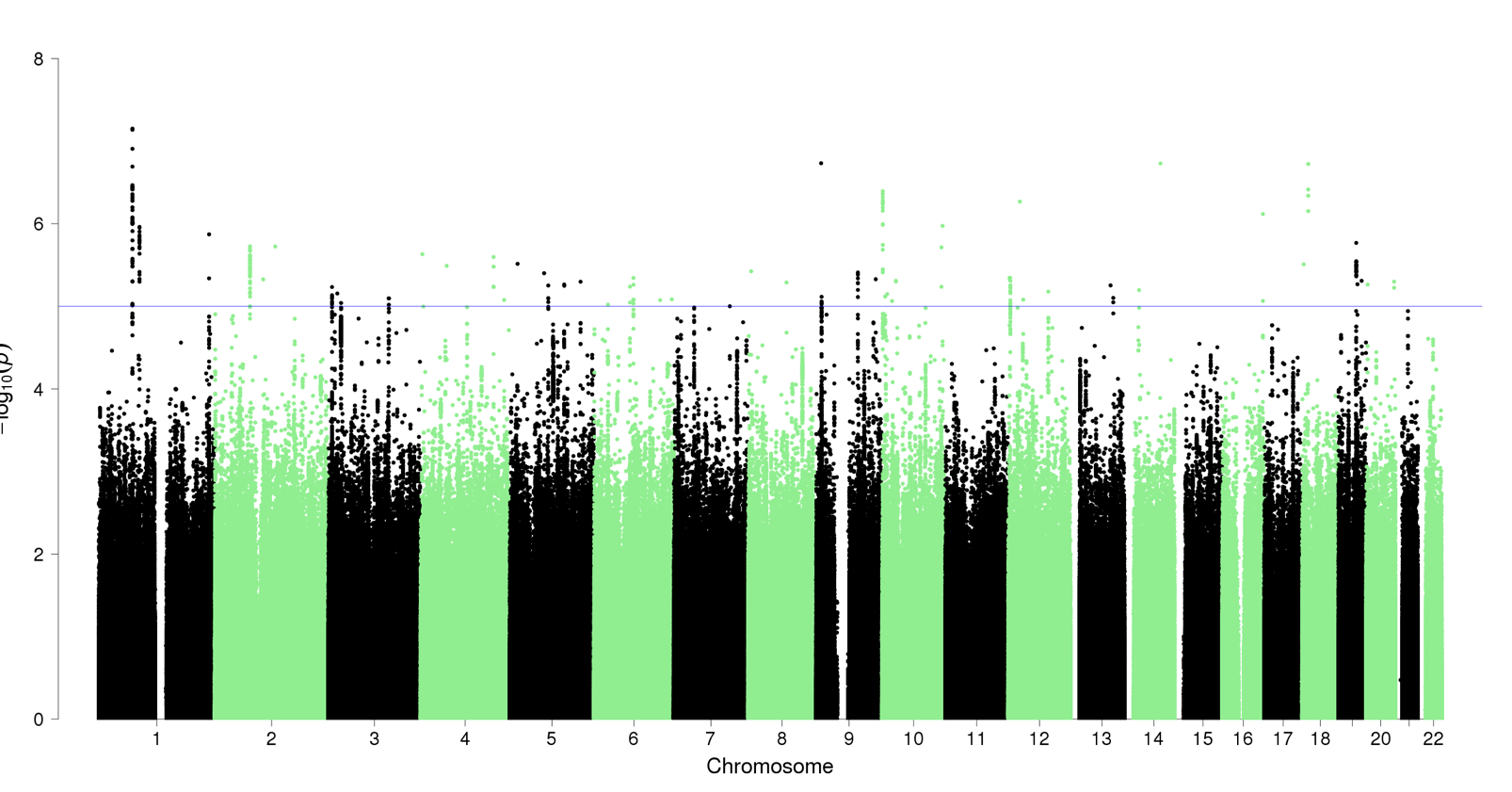

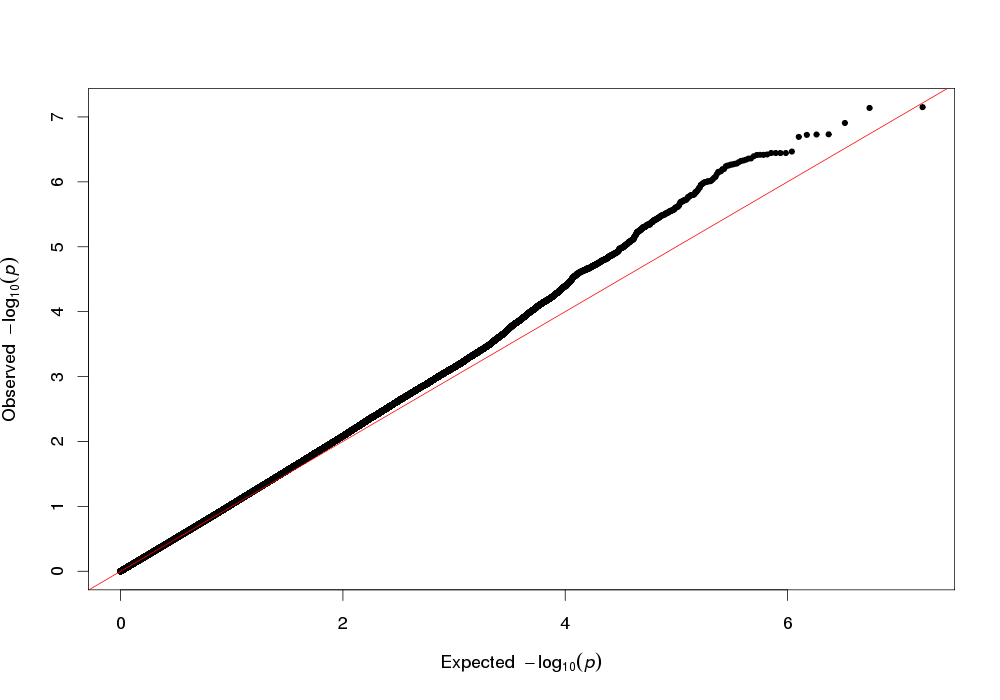
*

*HATA uncorrected for total hippocampal volume*

*
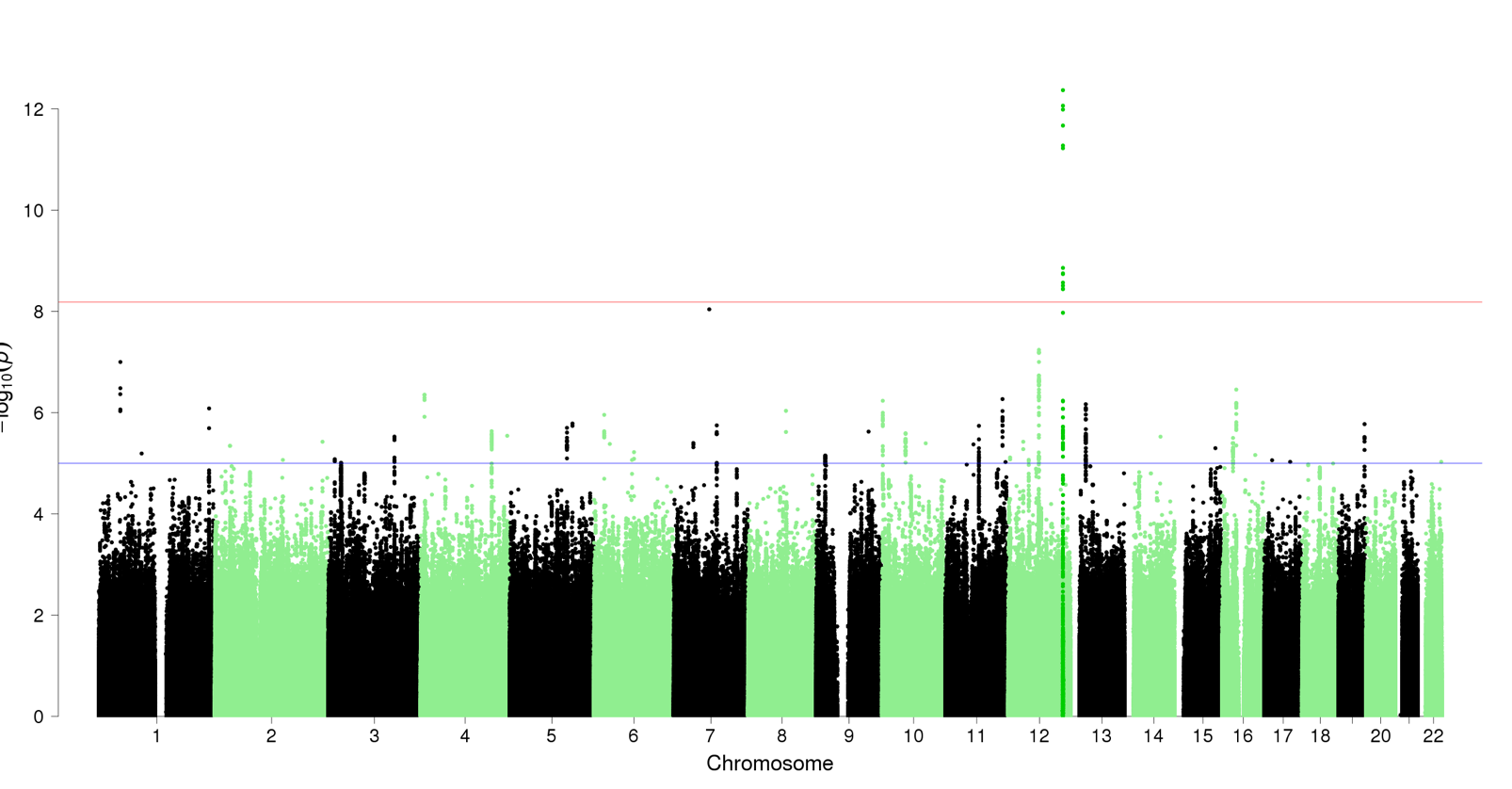

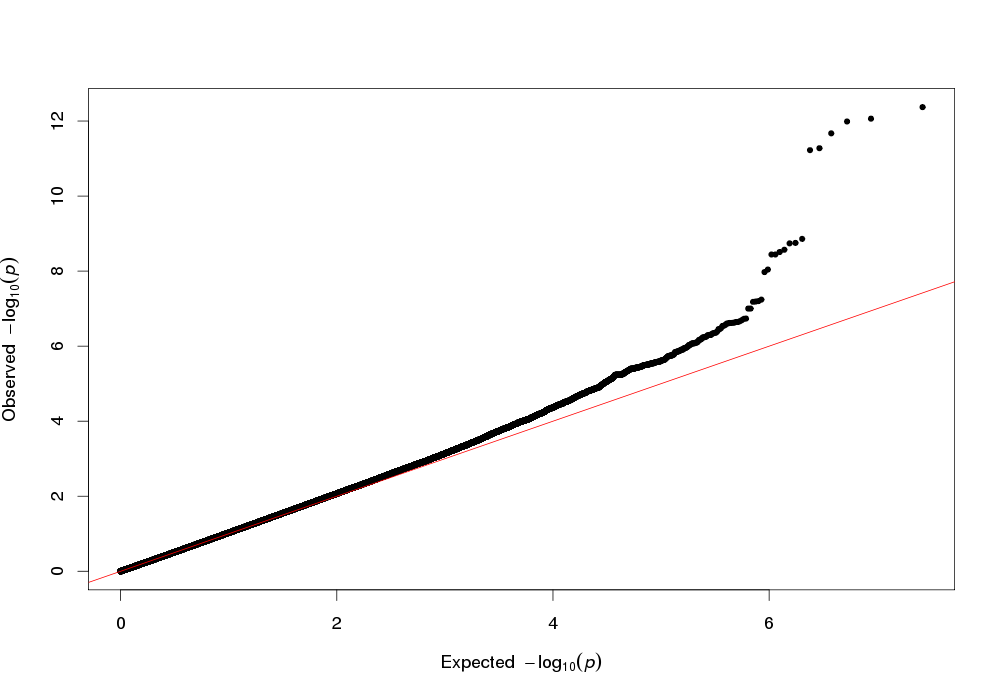
*

*Tail corrected for total hippocampal volume*

*
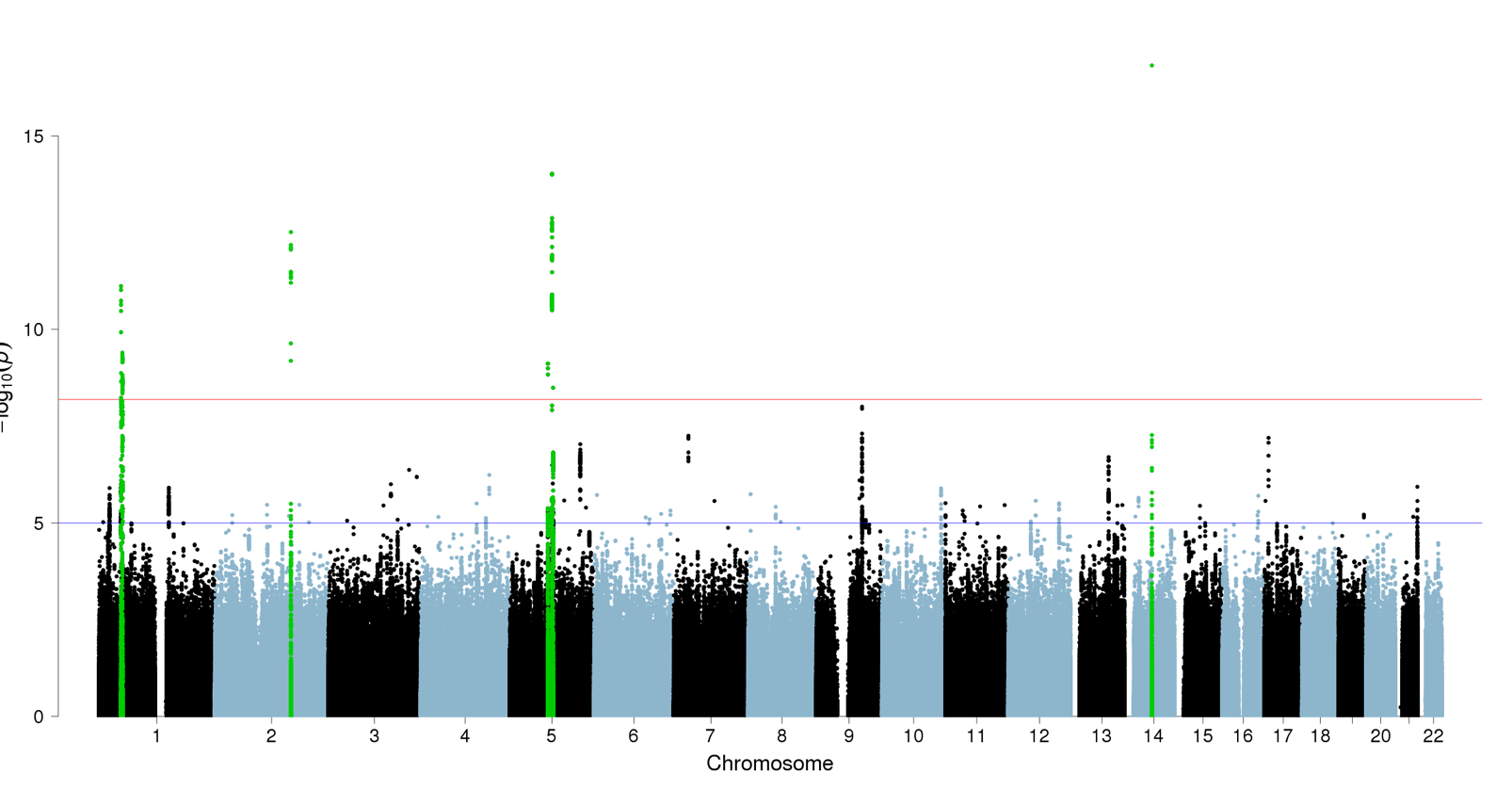

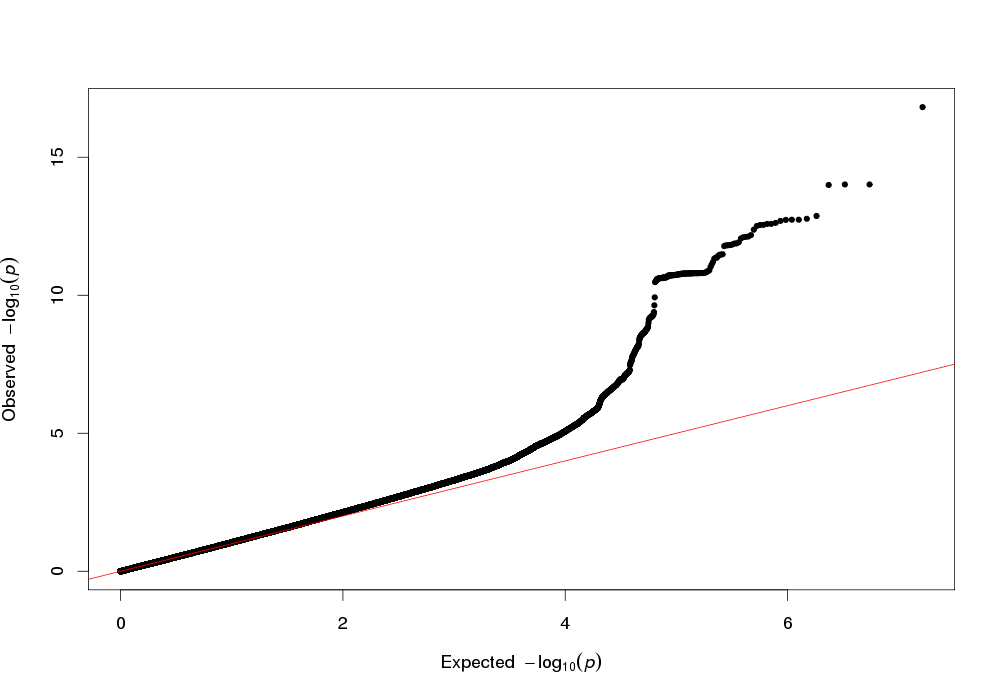
*

*Tail uncorrected for total hippocampal volume*

*
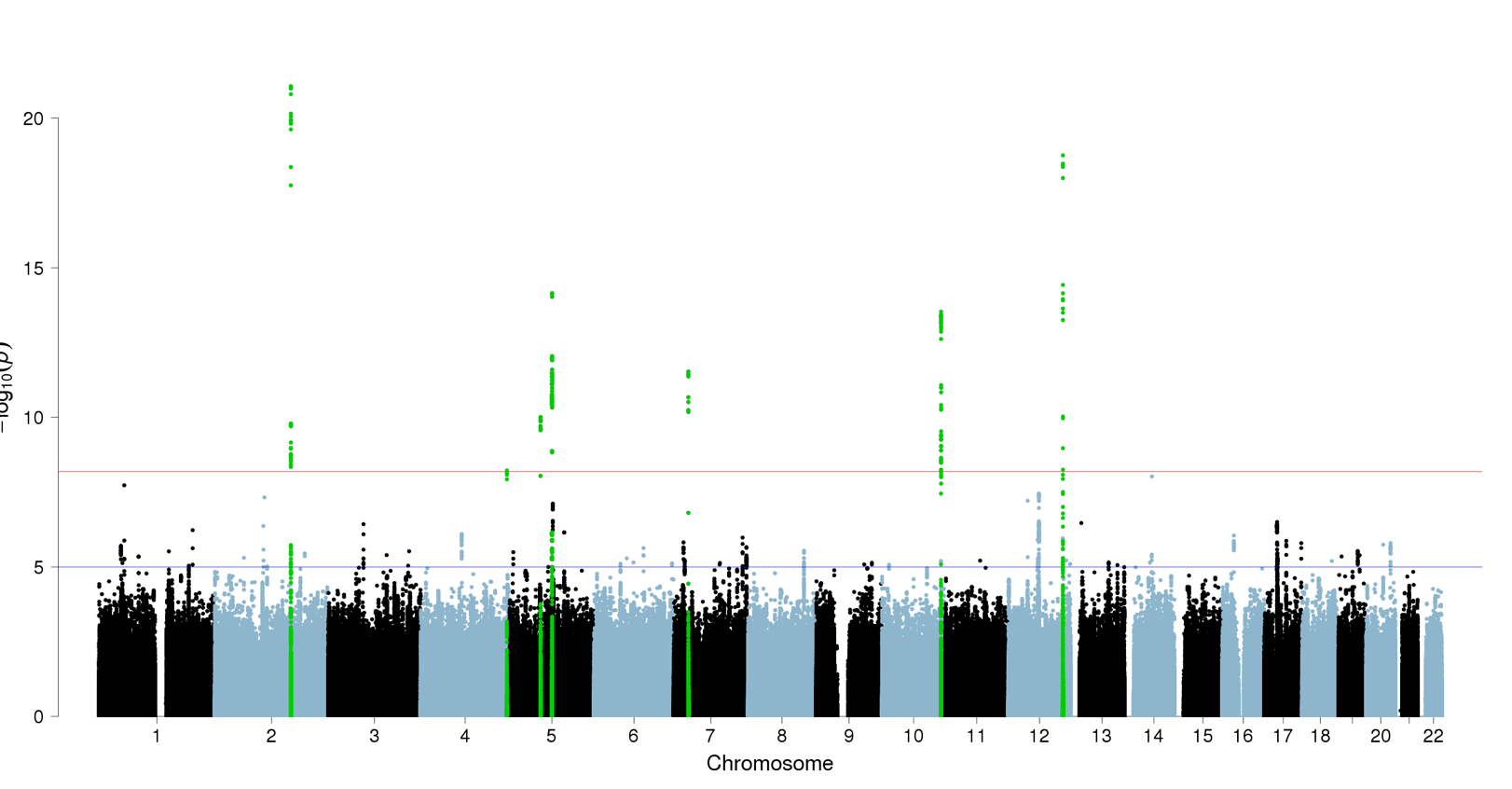

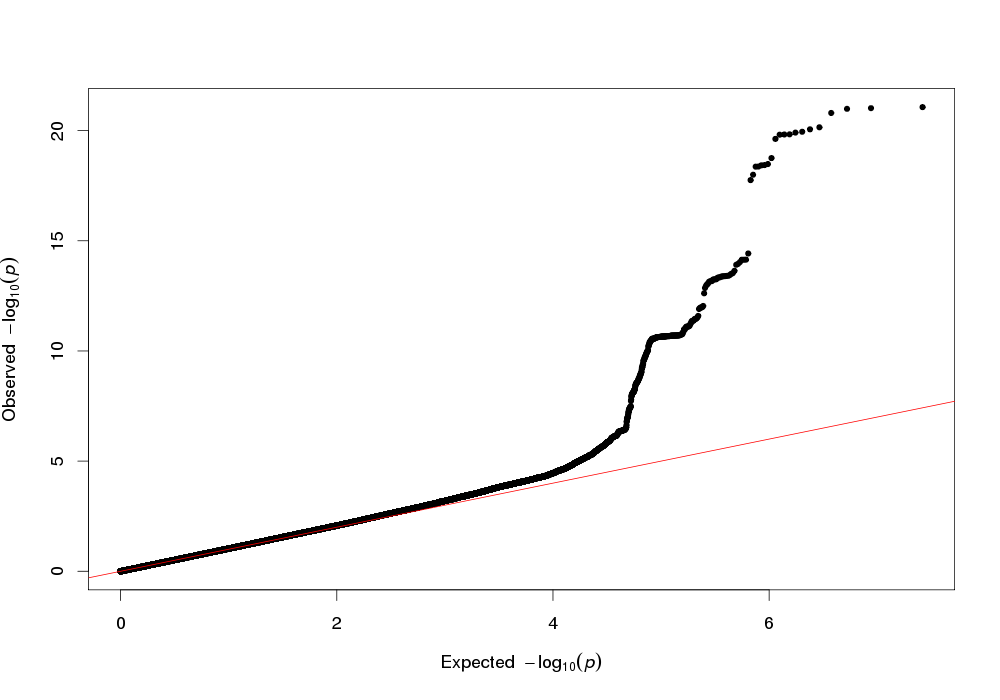
*

*Fissure corrected for total hippocampal volume*

*
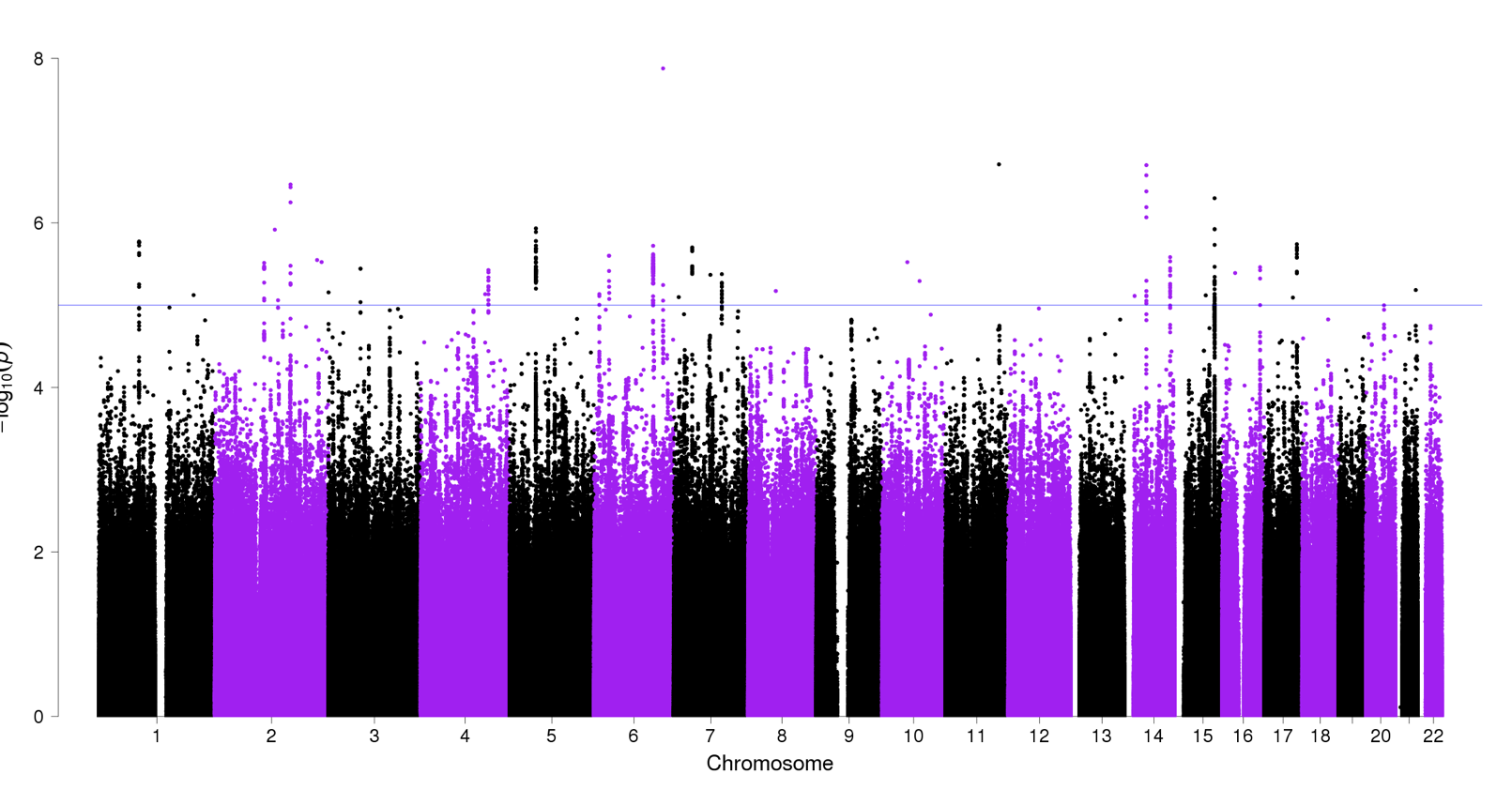

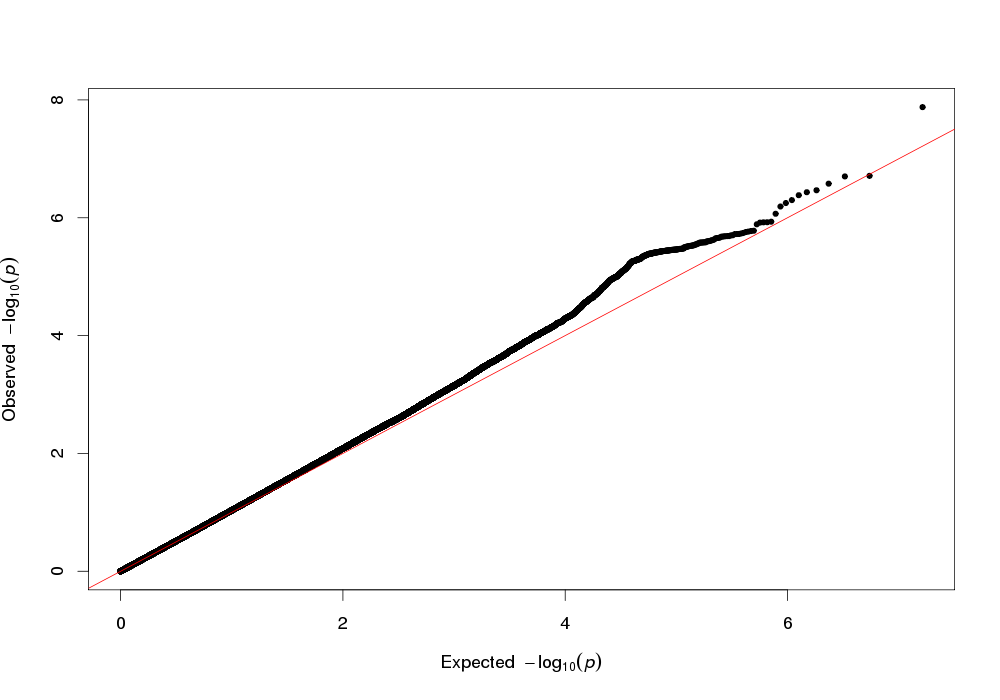
*

*Fissure uncorrected for total hippocampal volume*


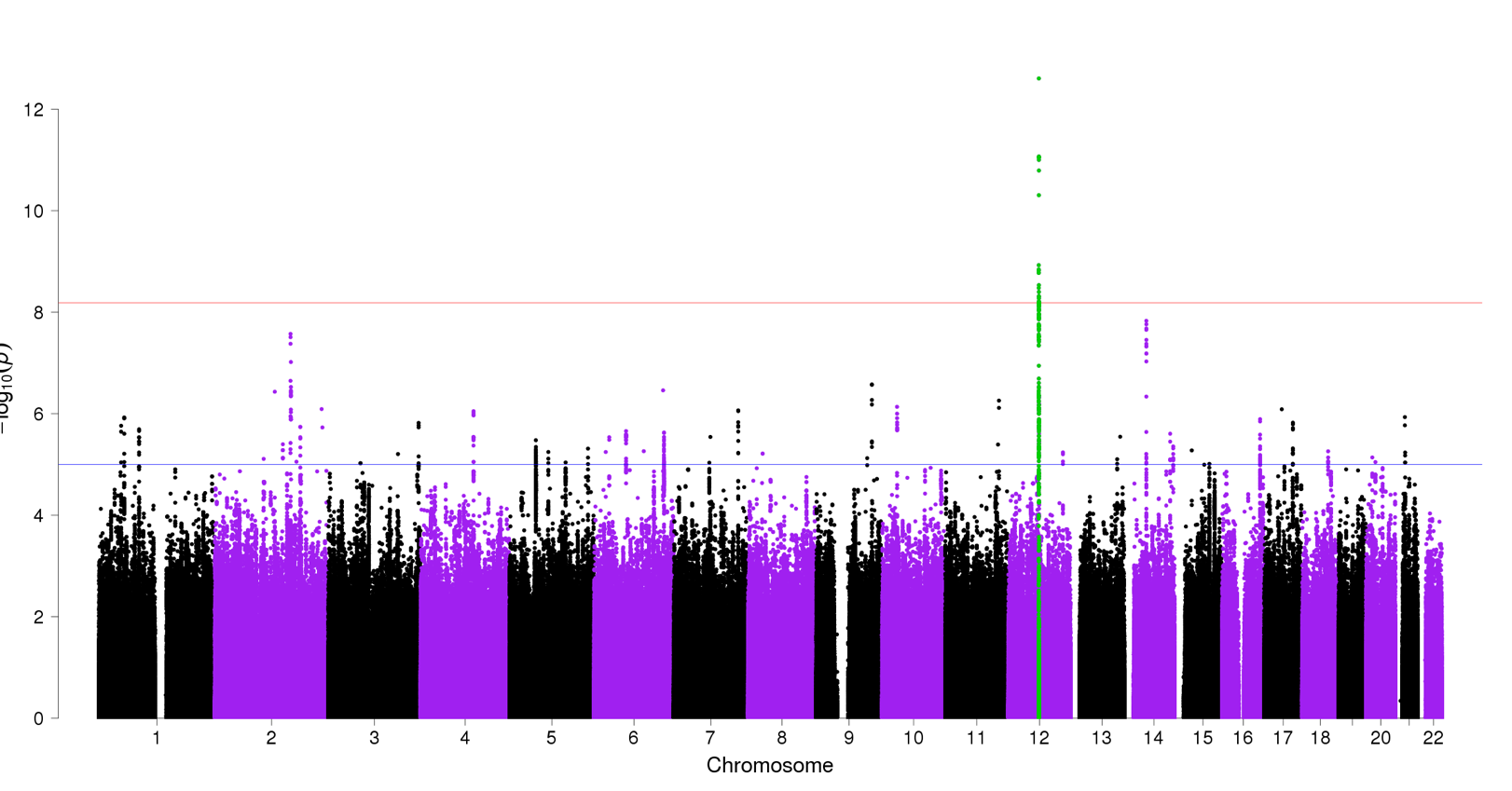

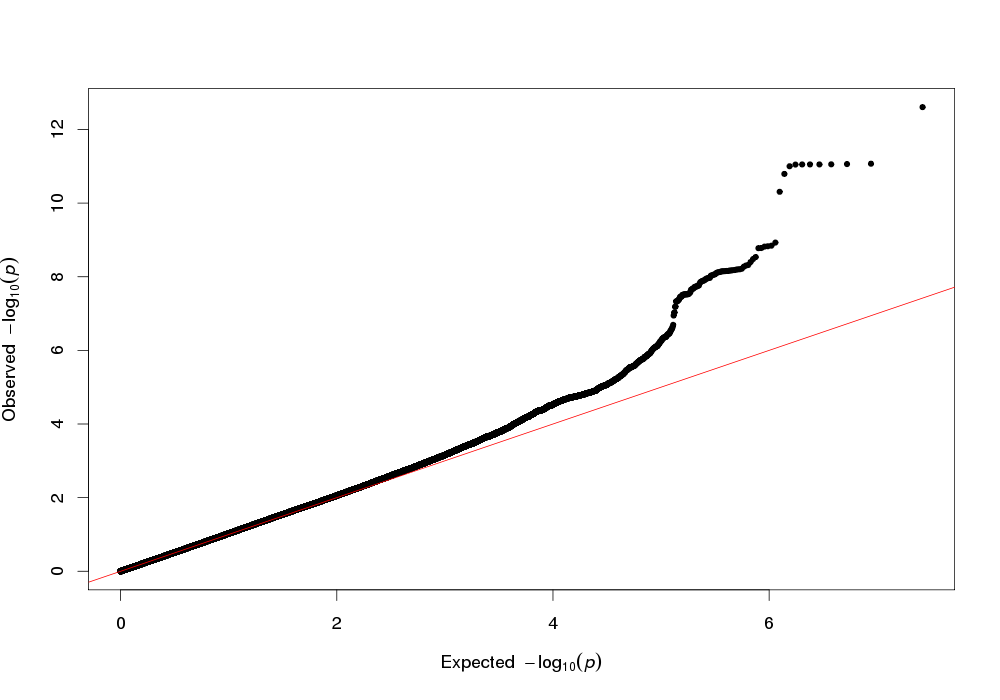


*Molecular Layer corrected for total hippocampal volume*

*
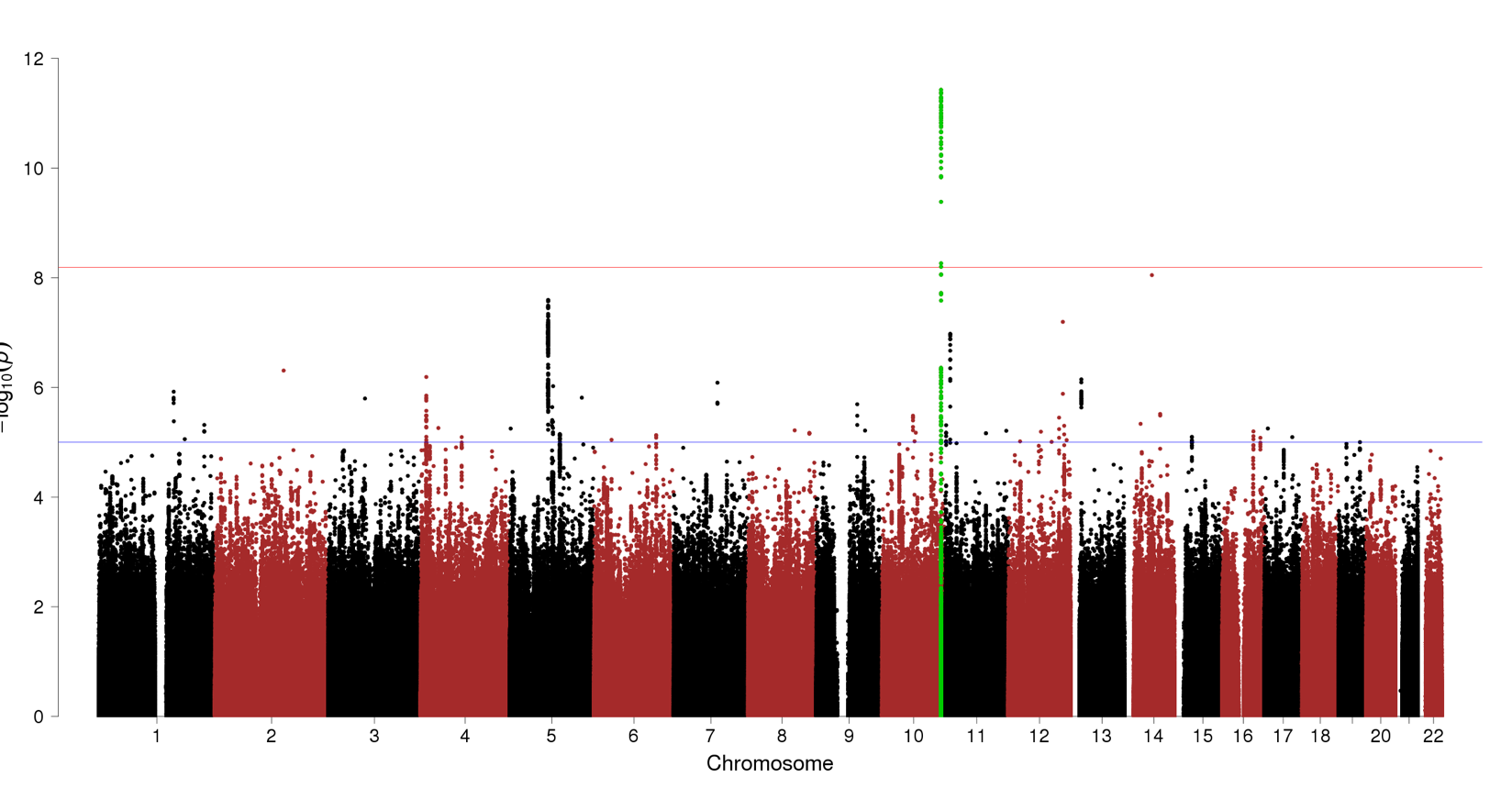

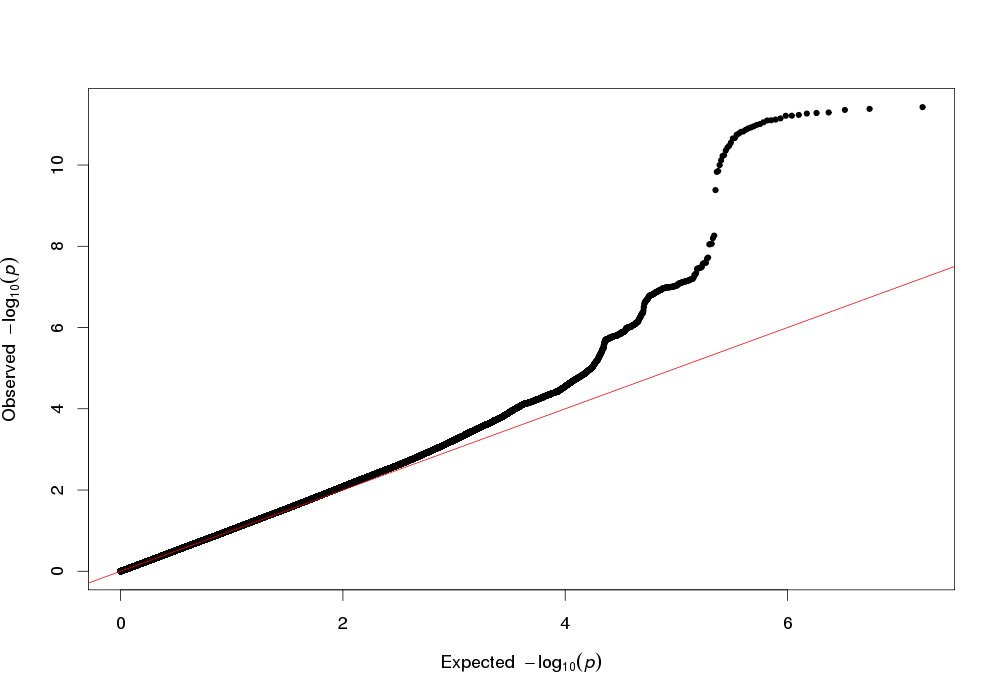
*

*Molecular Layer uncorrected for total hippocampal volume*


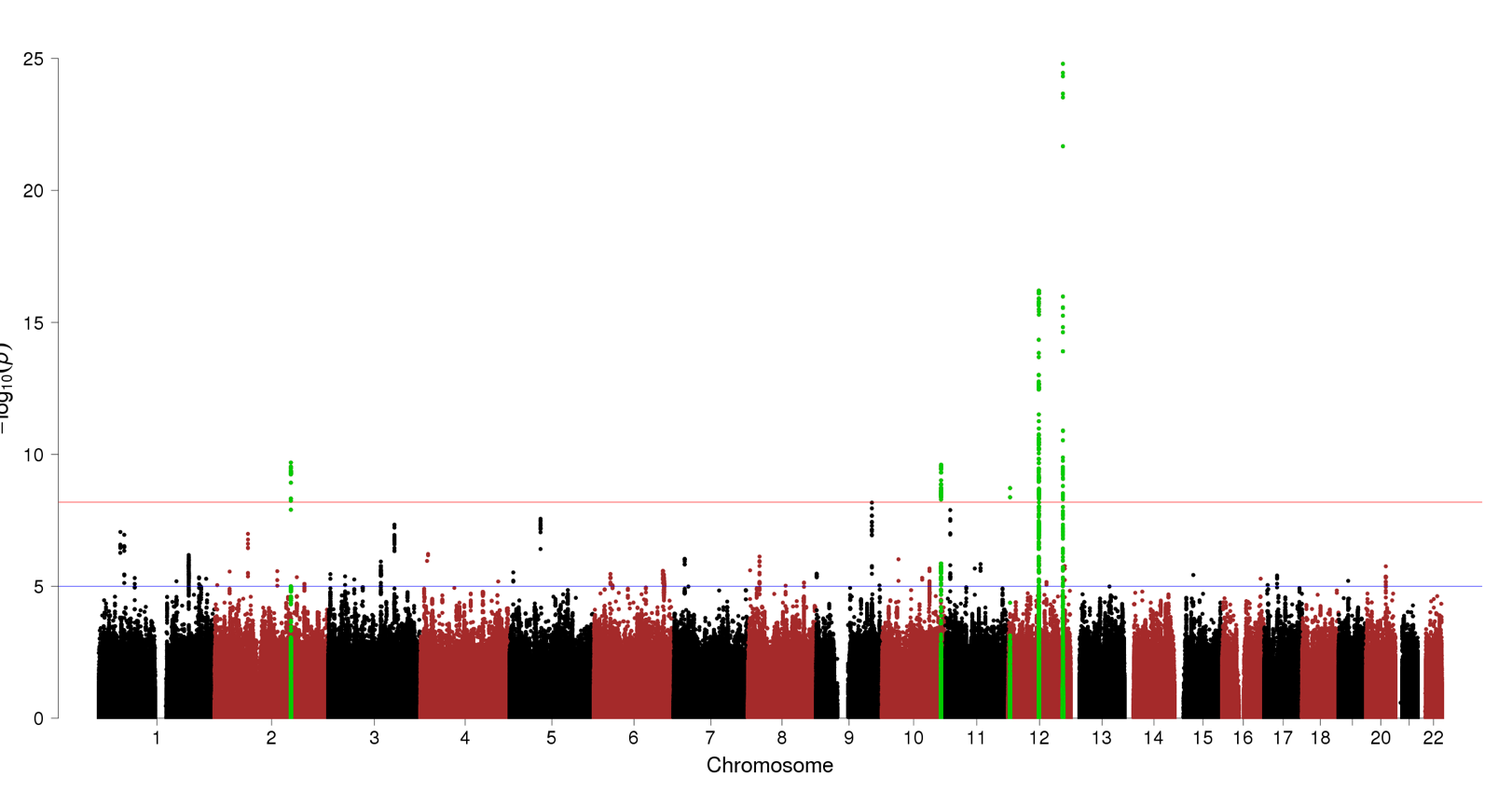

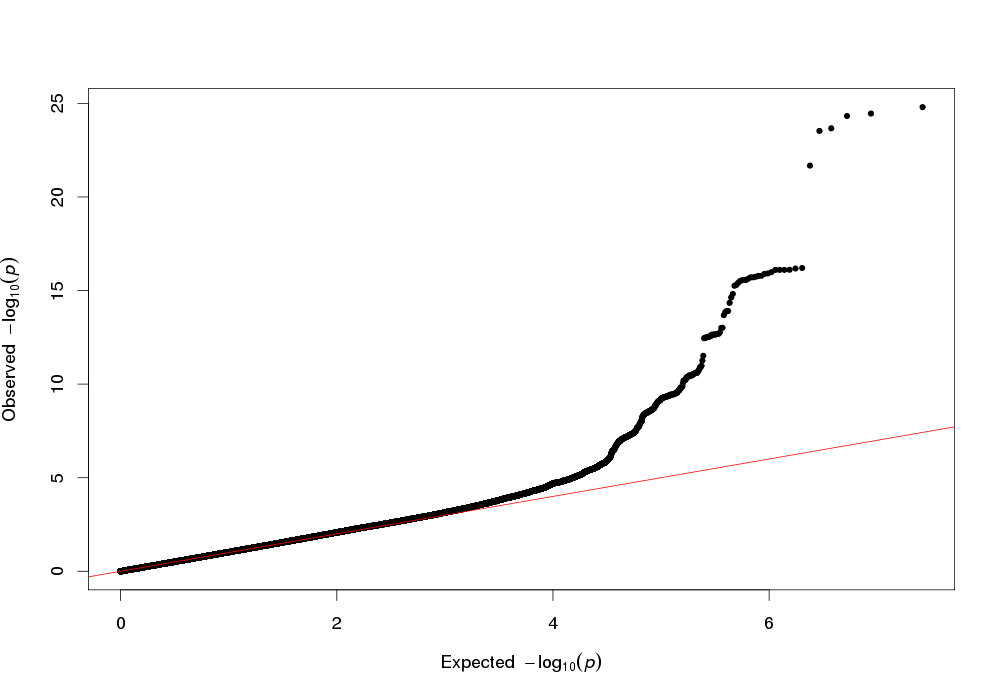


*Parasubiculum corrected for total hippocampal volume*

*
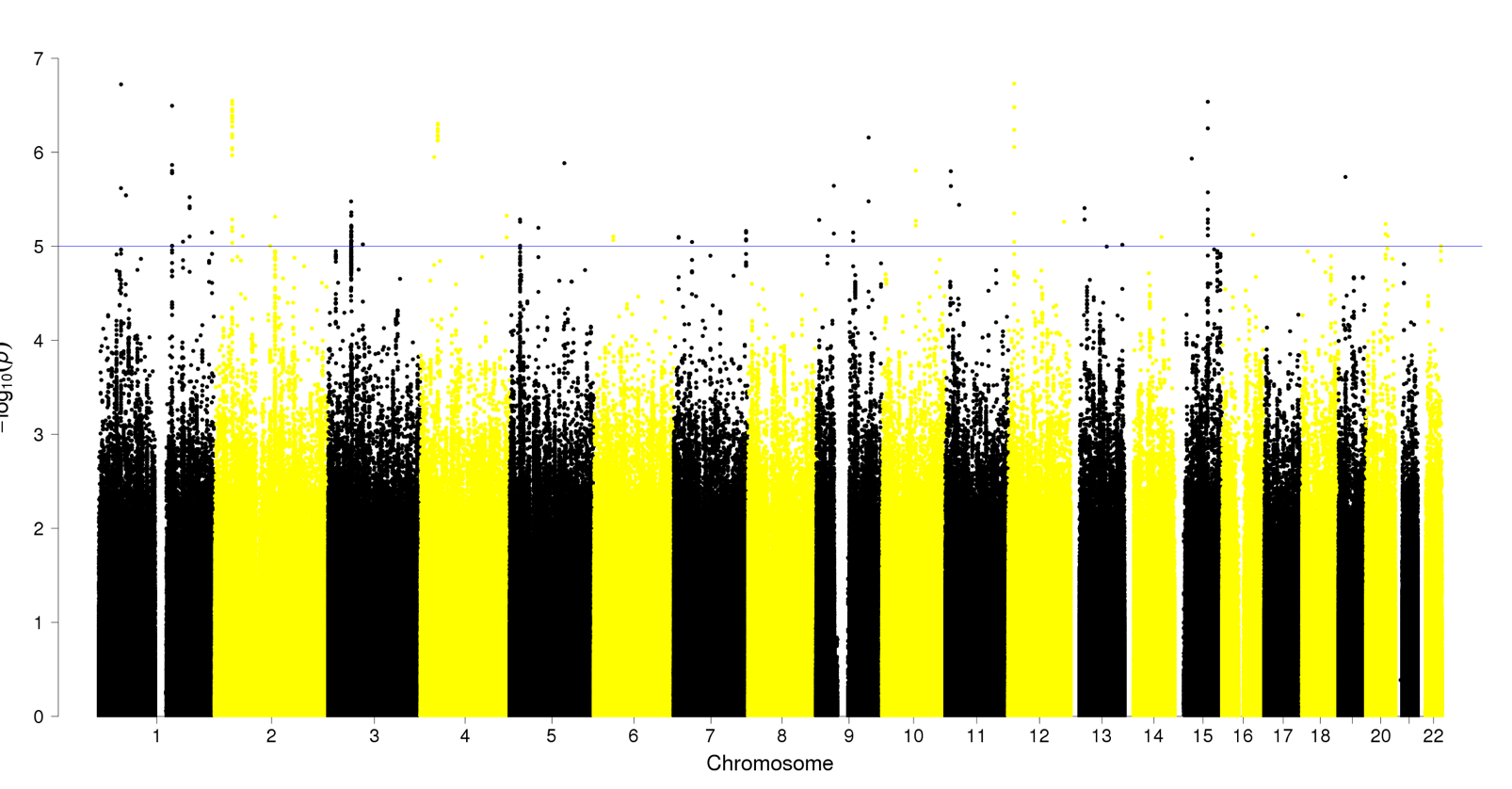

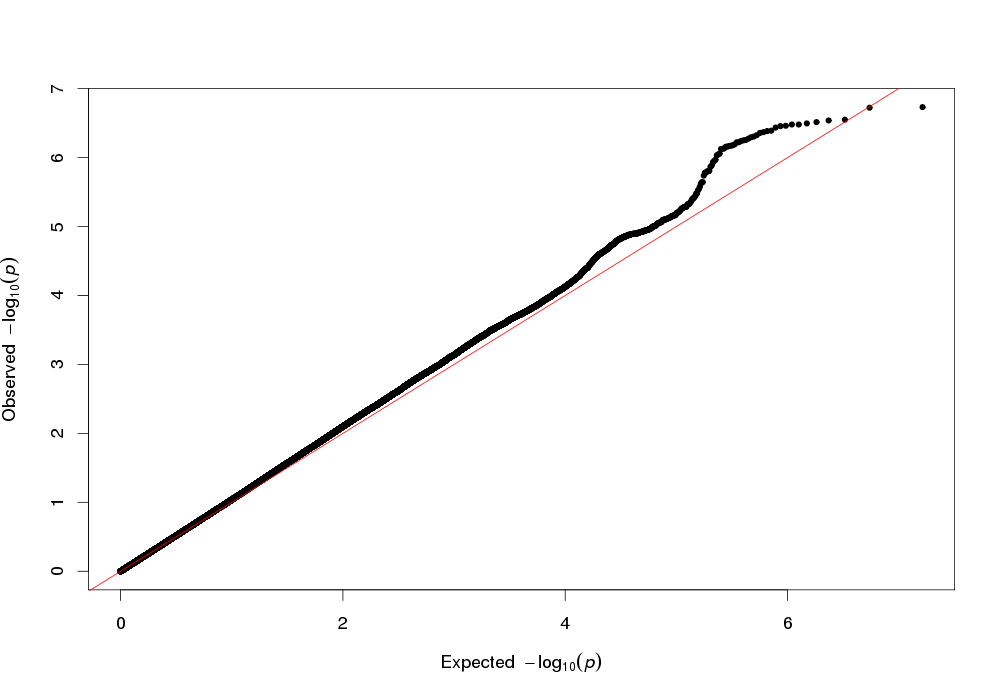
*

*Parasubiculum uncorrected for total hippocampal volume*


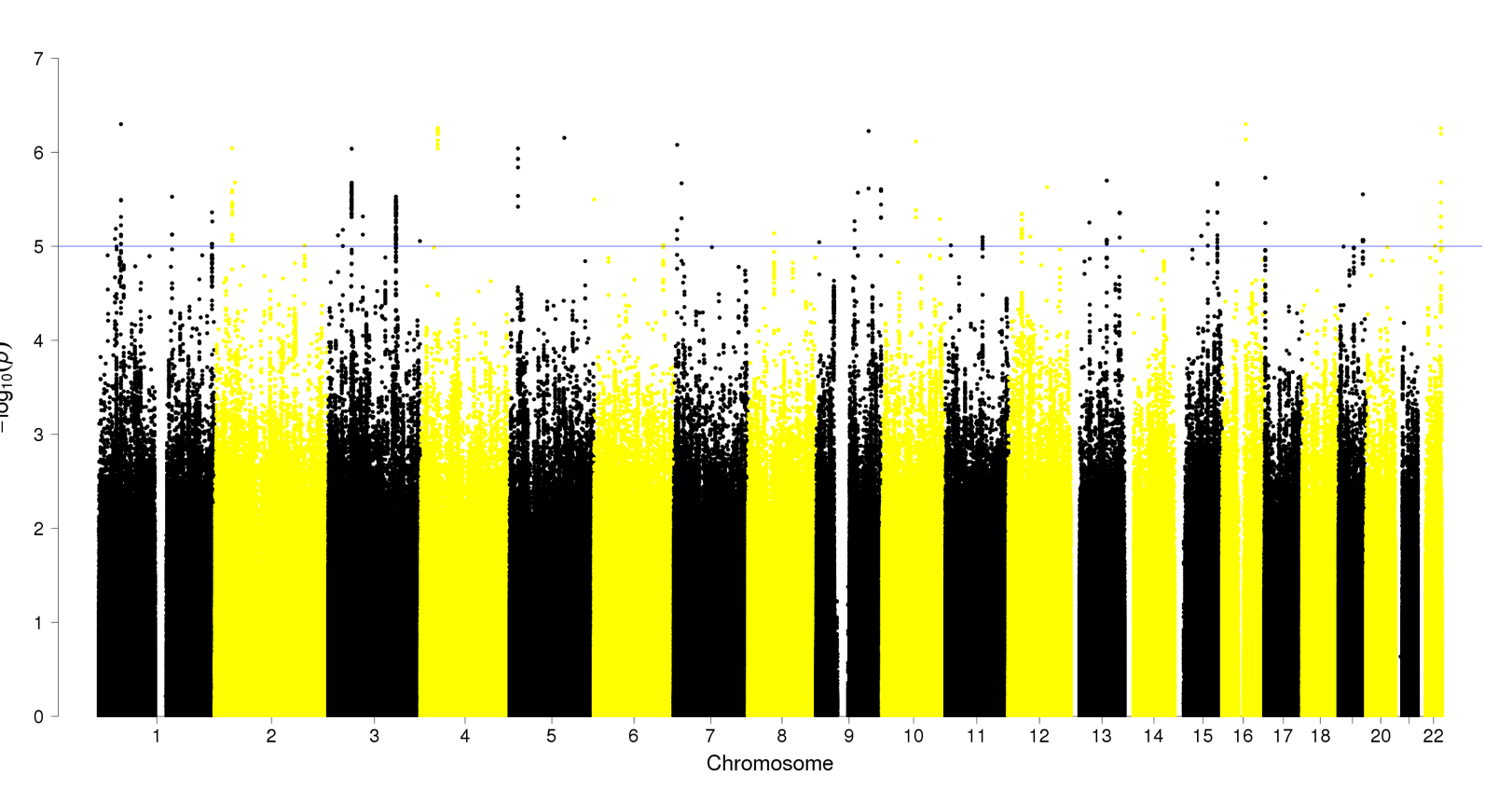

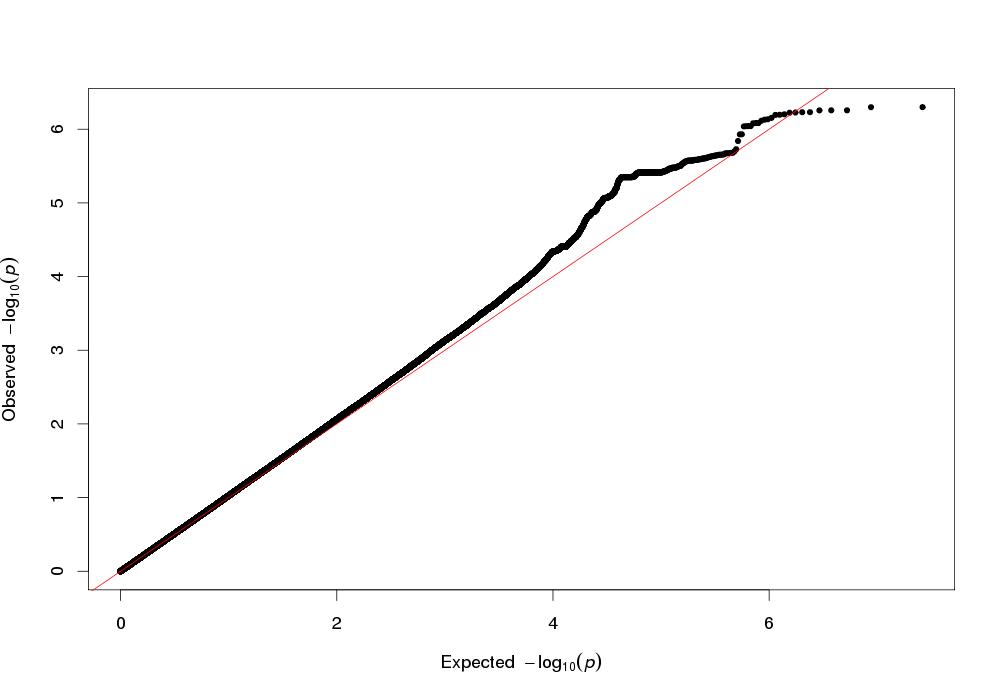


*Presubiculum corrected for total hippocampal volume*

*
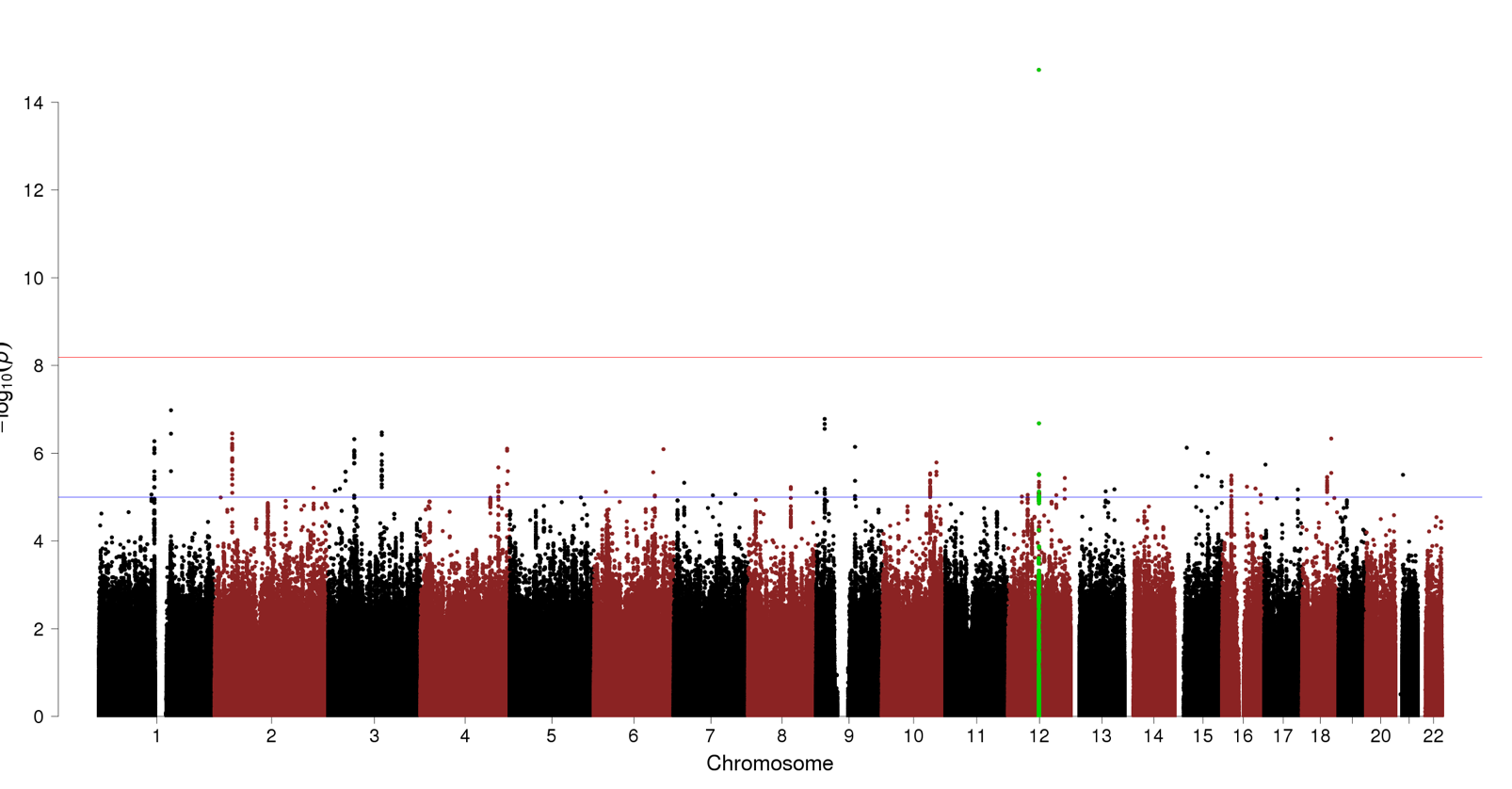

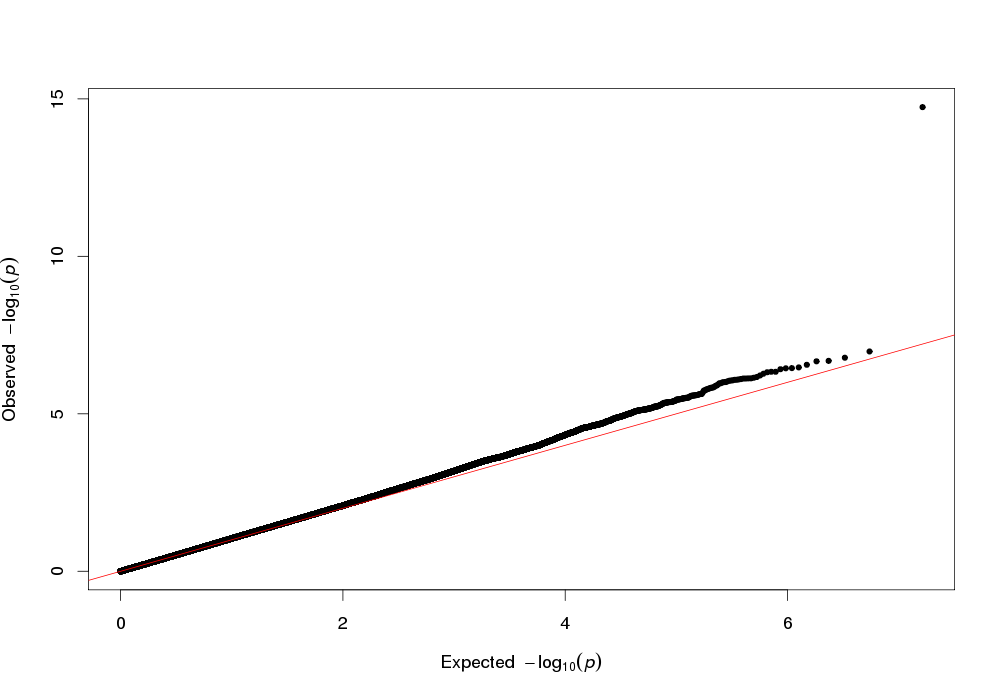
*

*Presubiculum uncorrected for total hippocampal volume*


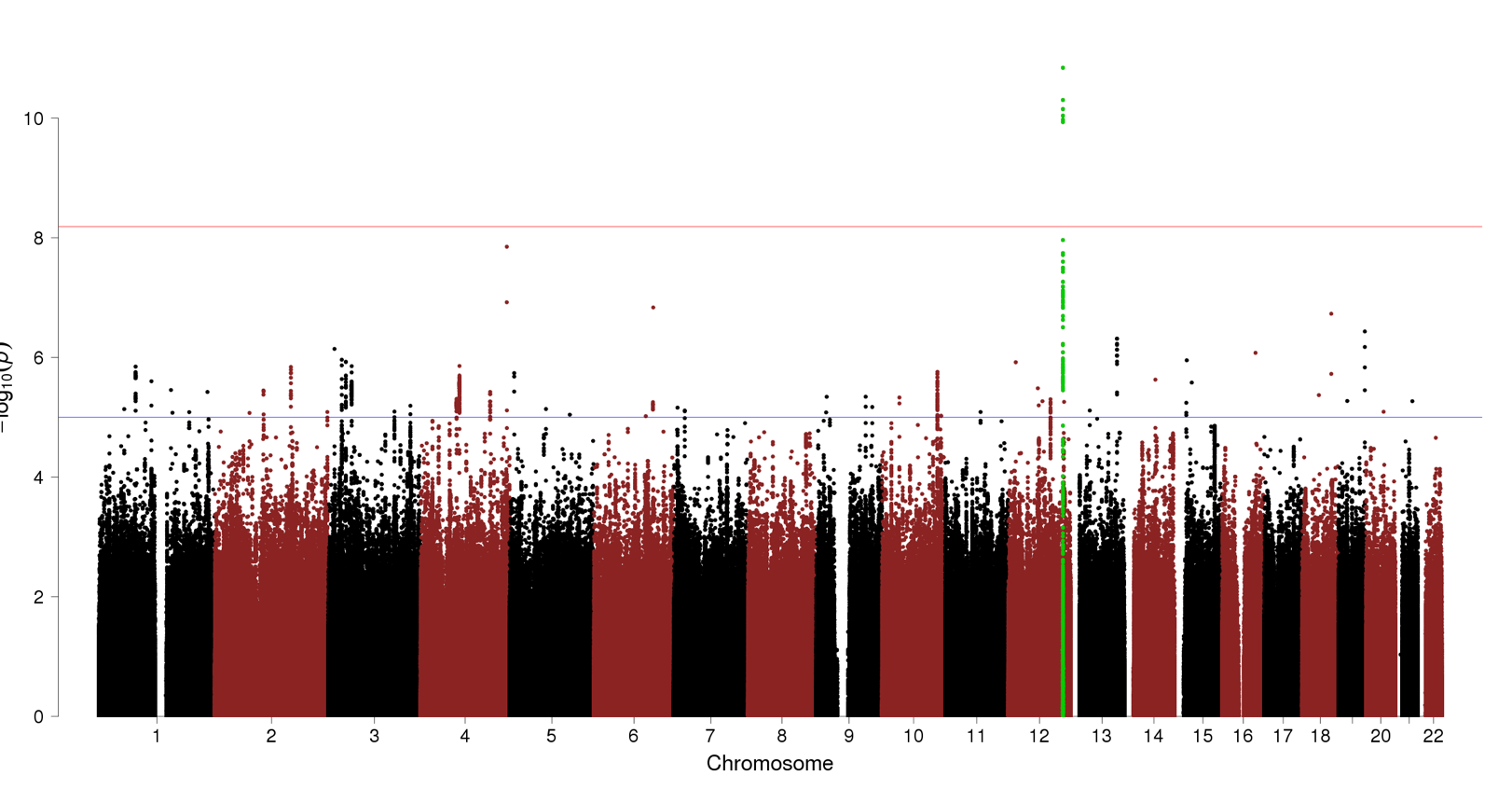

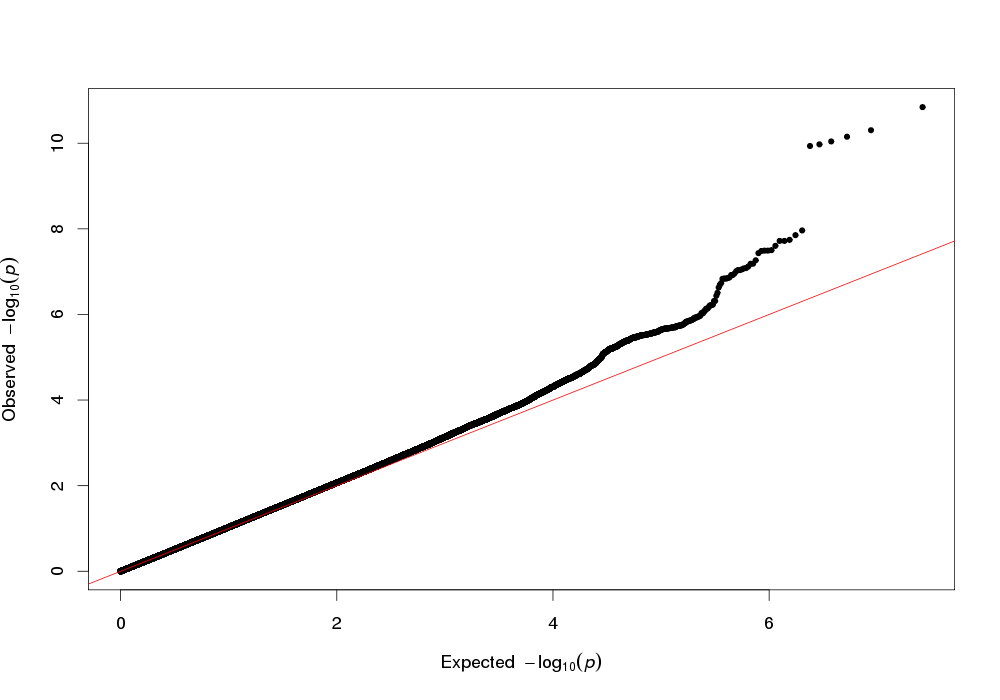


*Subiculum corrected for total hippocampal volume*

*
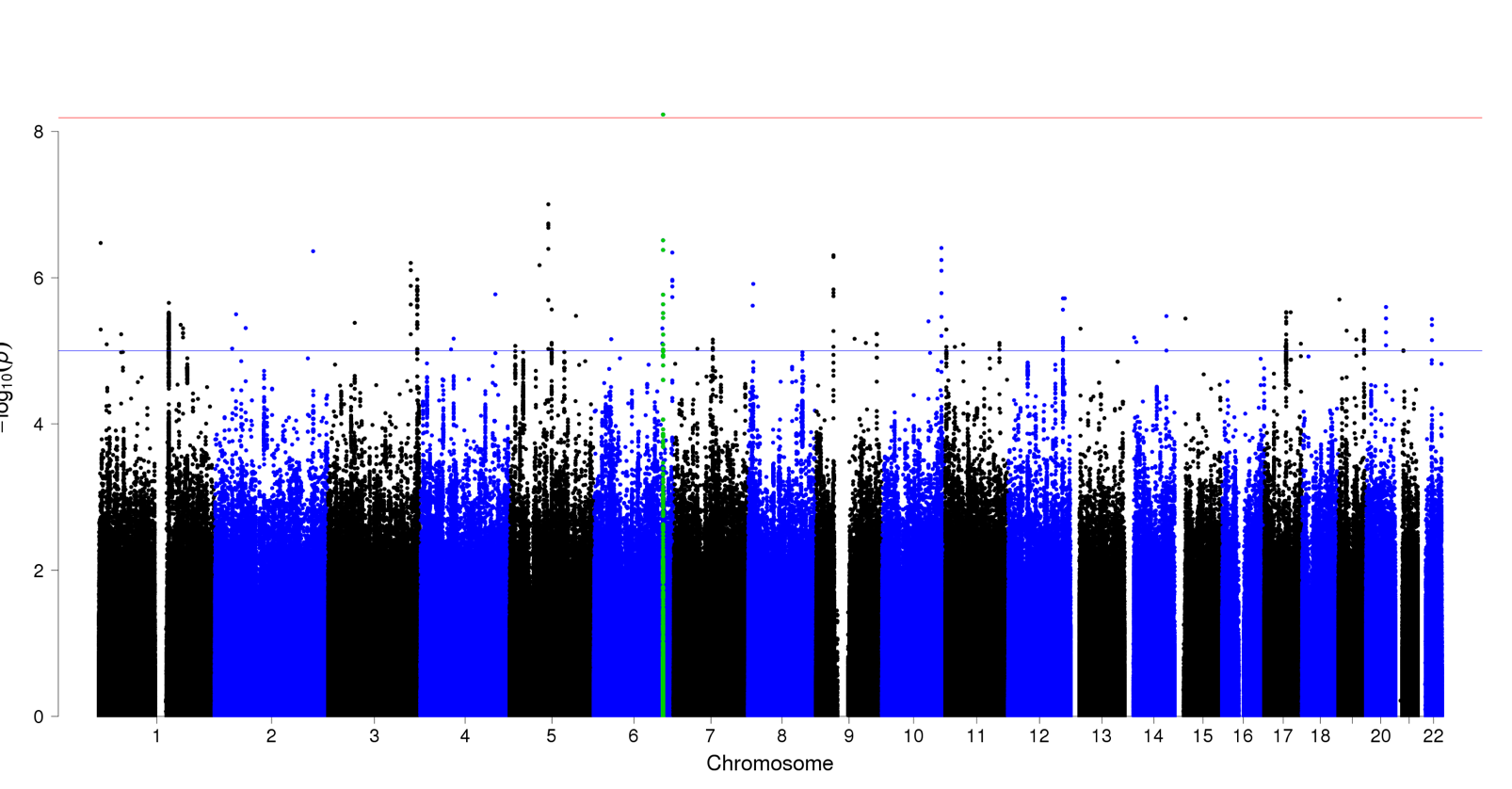

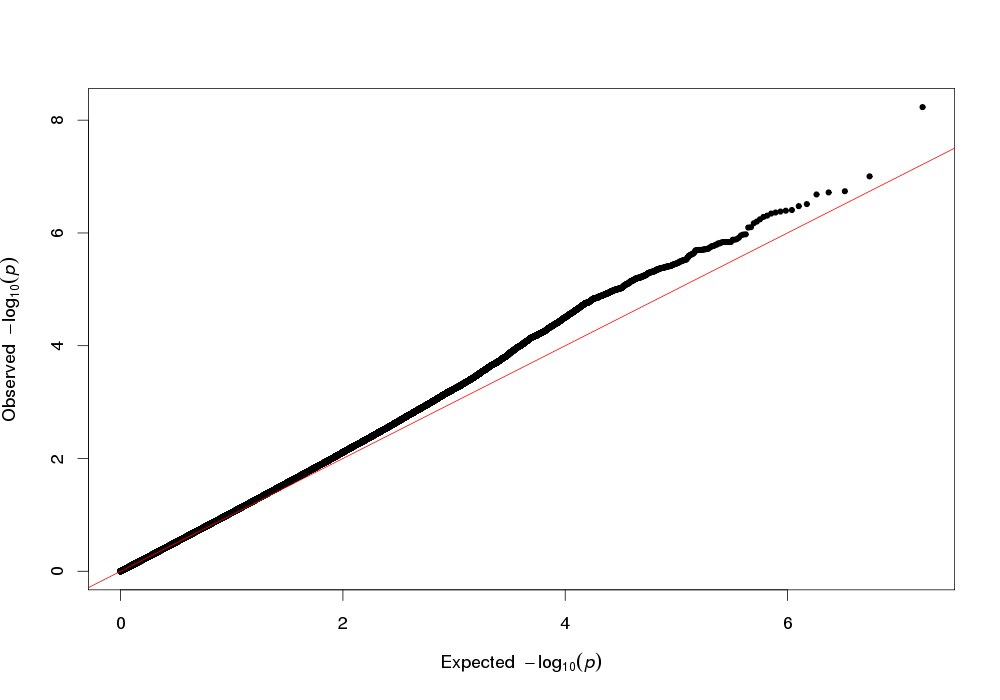
*

*Subiculum uncorrected for total hippocampal volume*


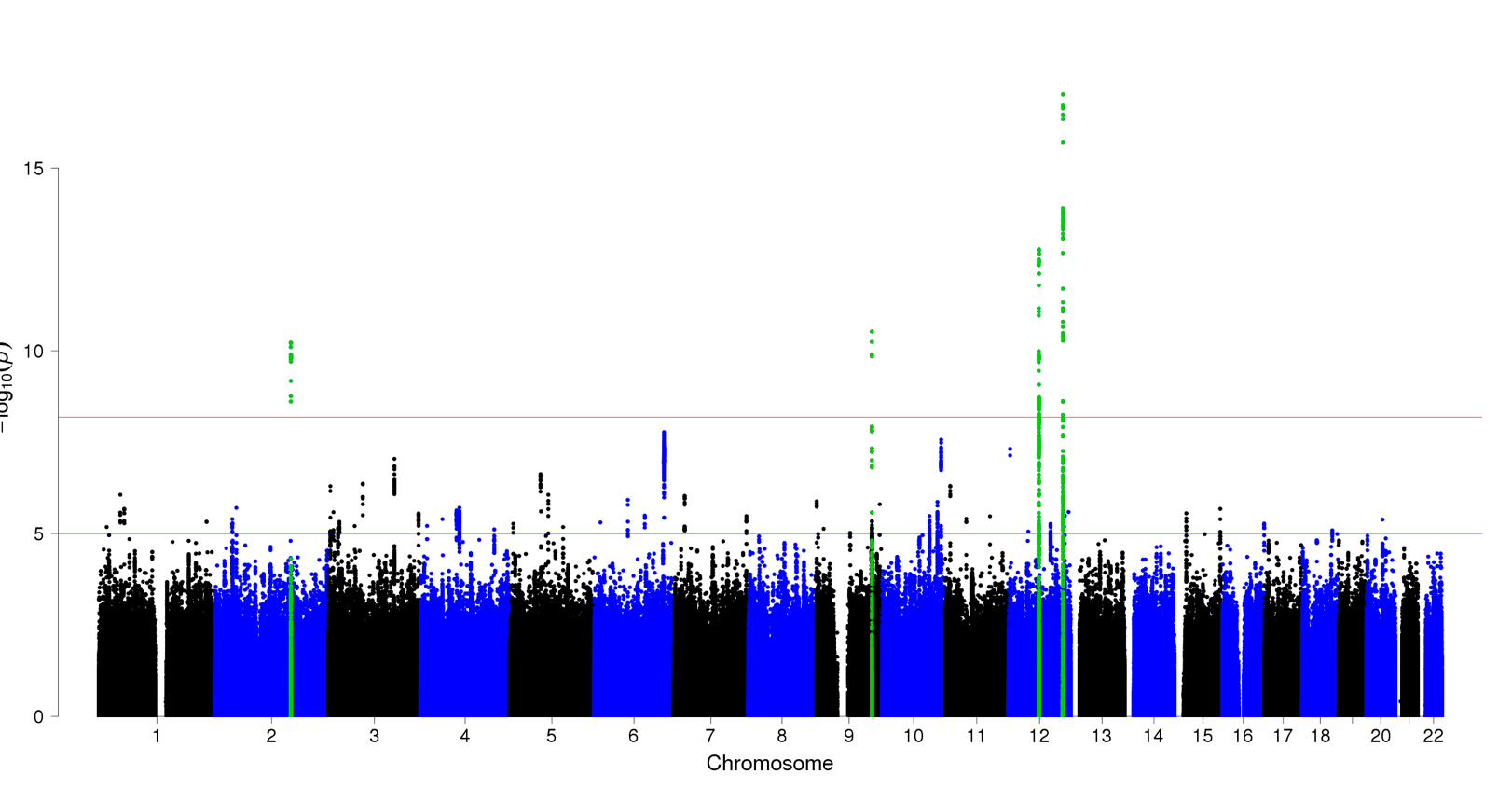

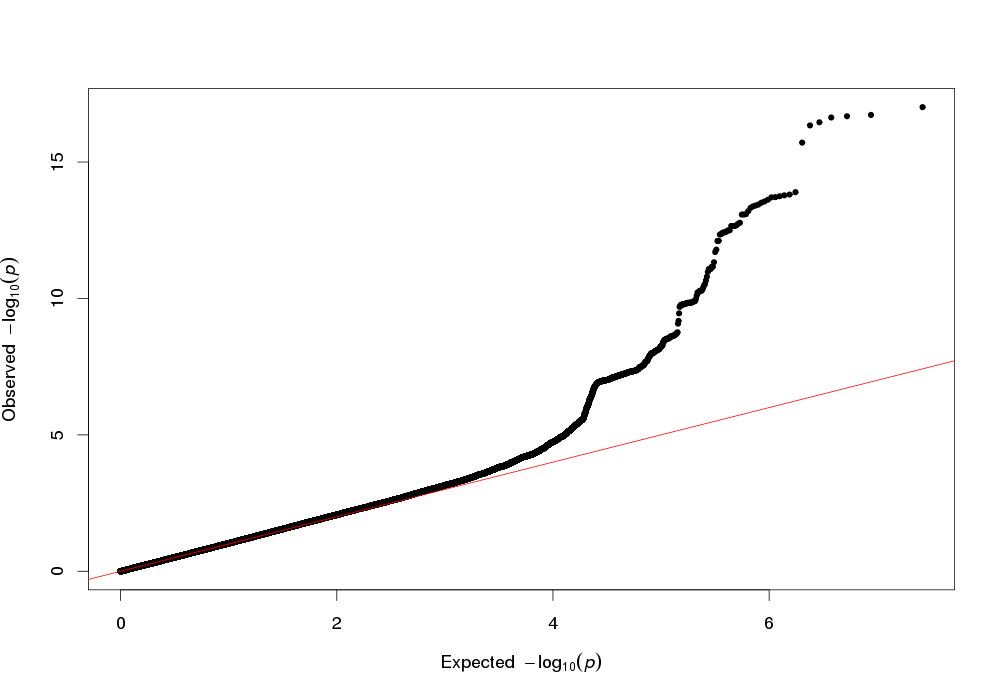


*Figure S4* *Forest plot per lead SNP of the whole-genome significant loci across all the subfields.*


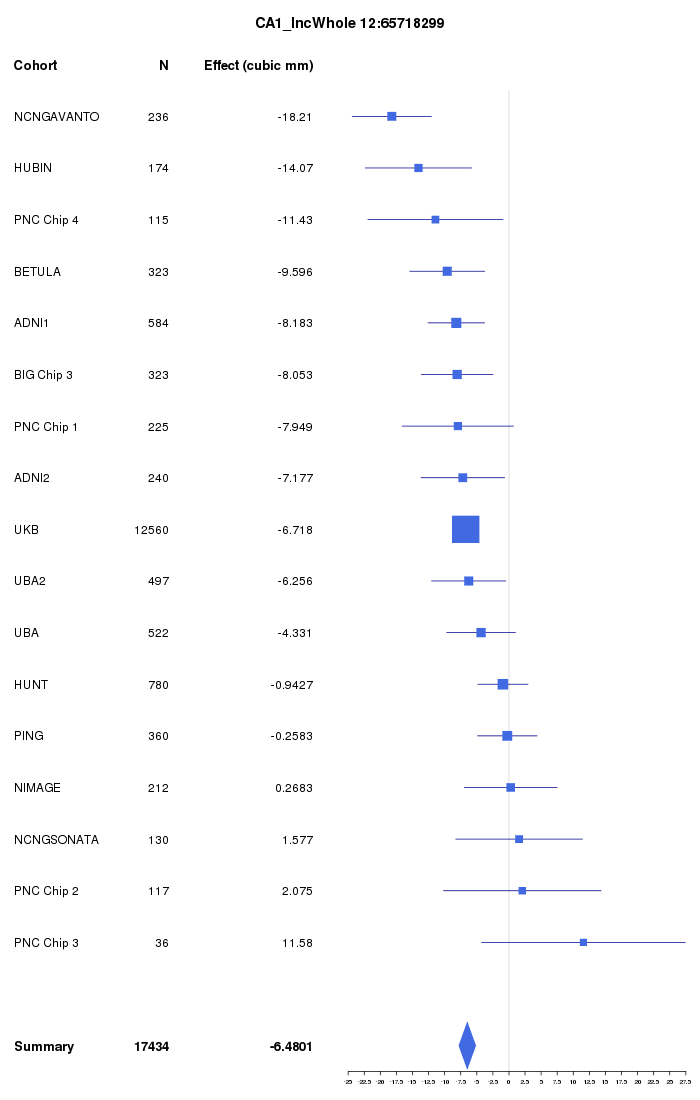


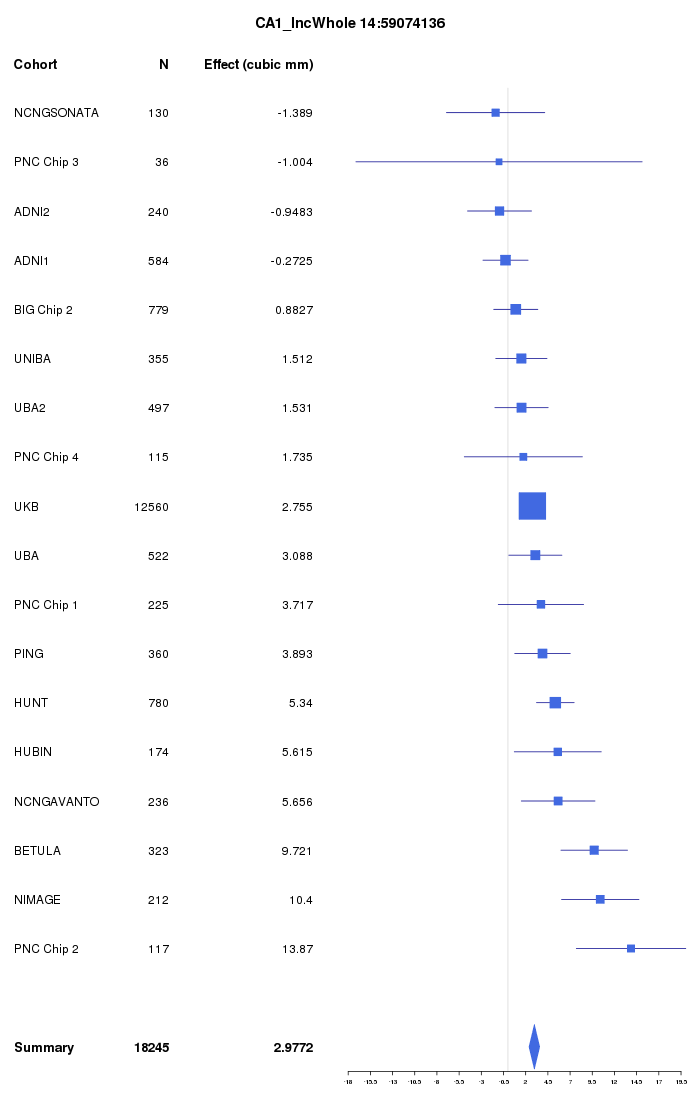

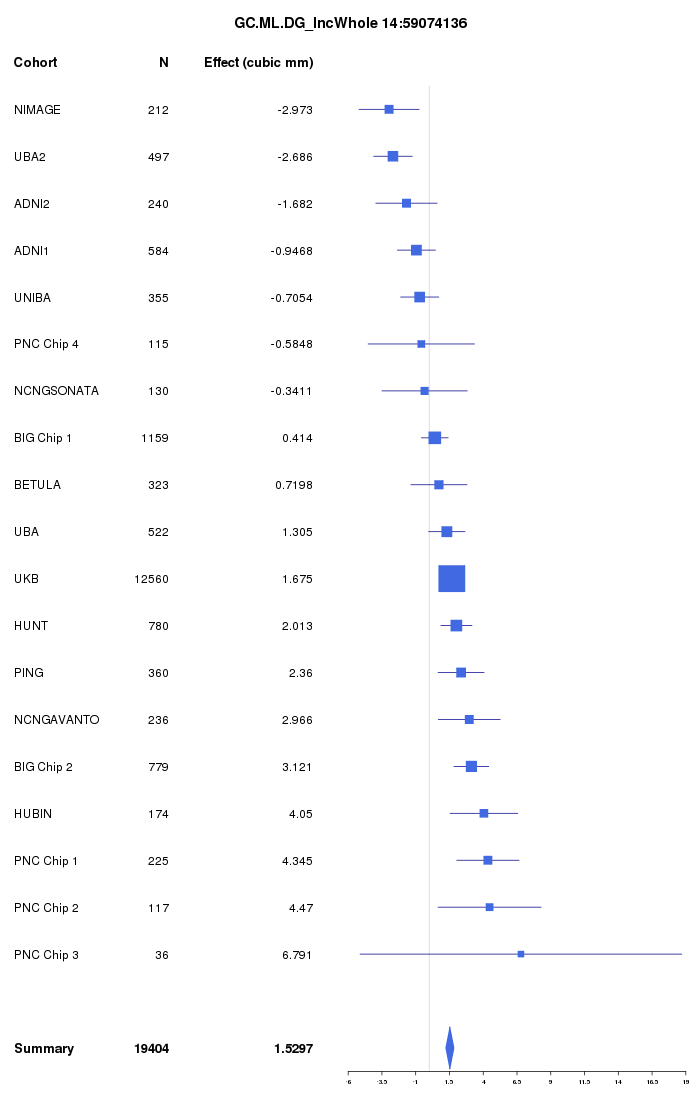

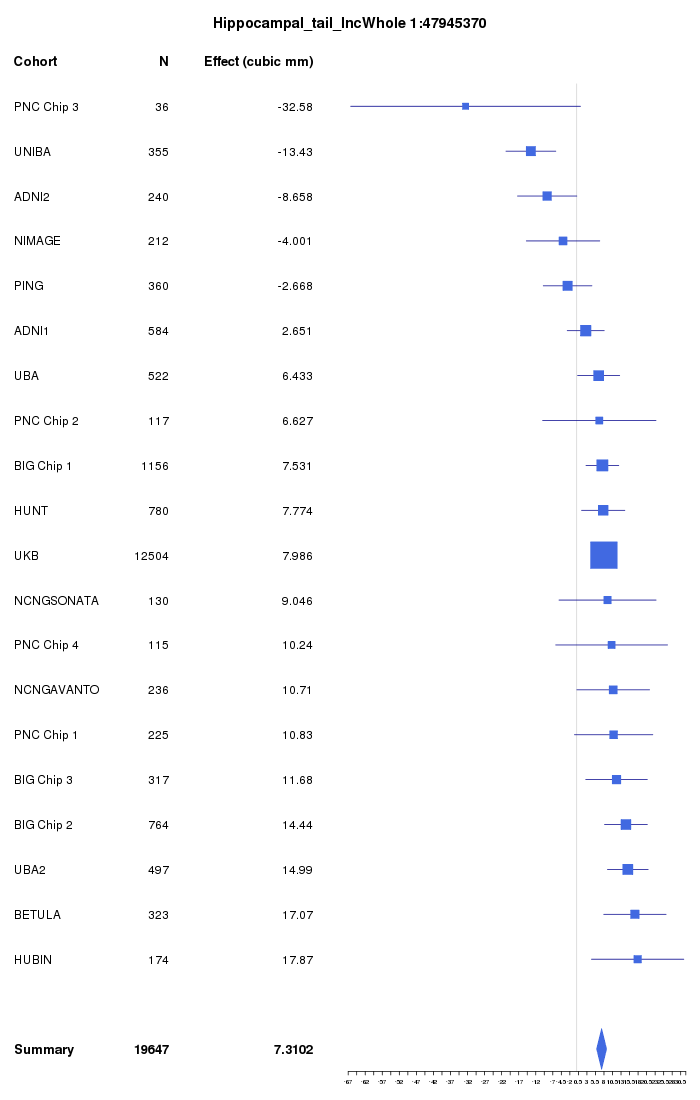

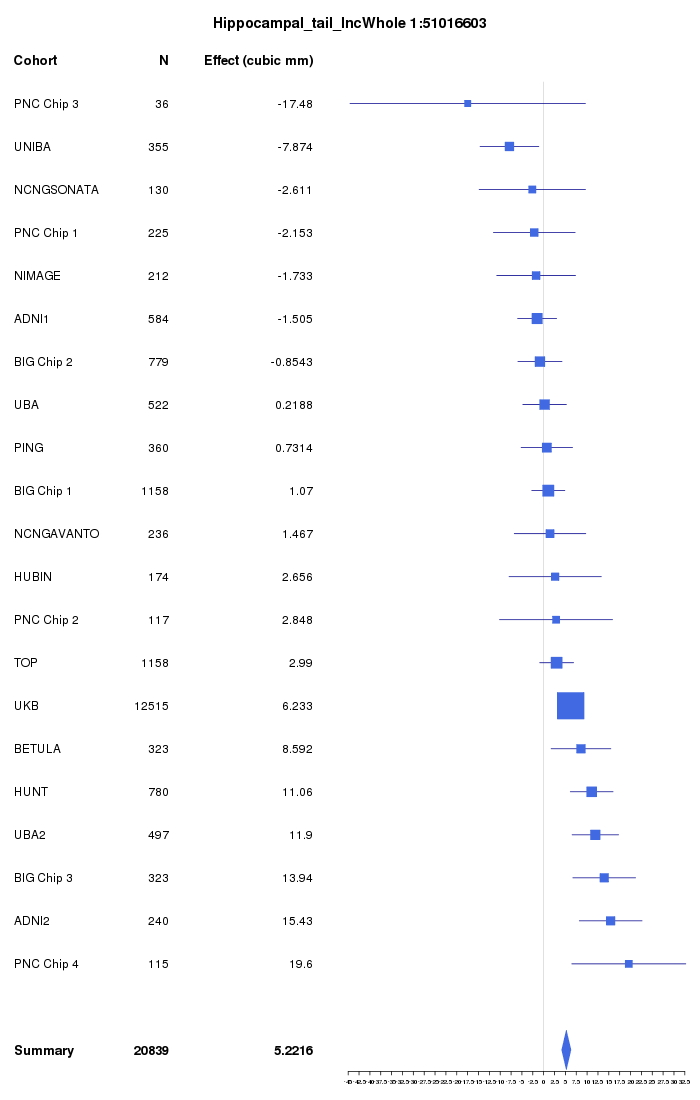

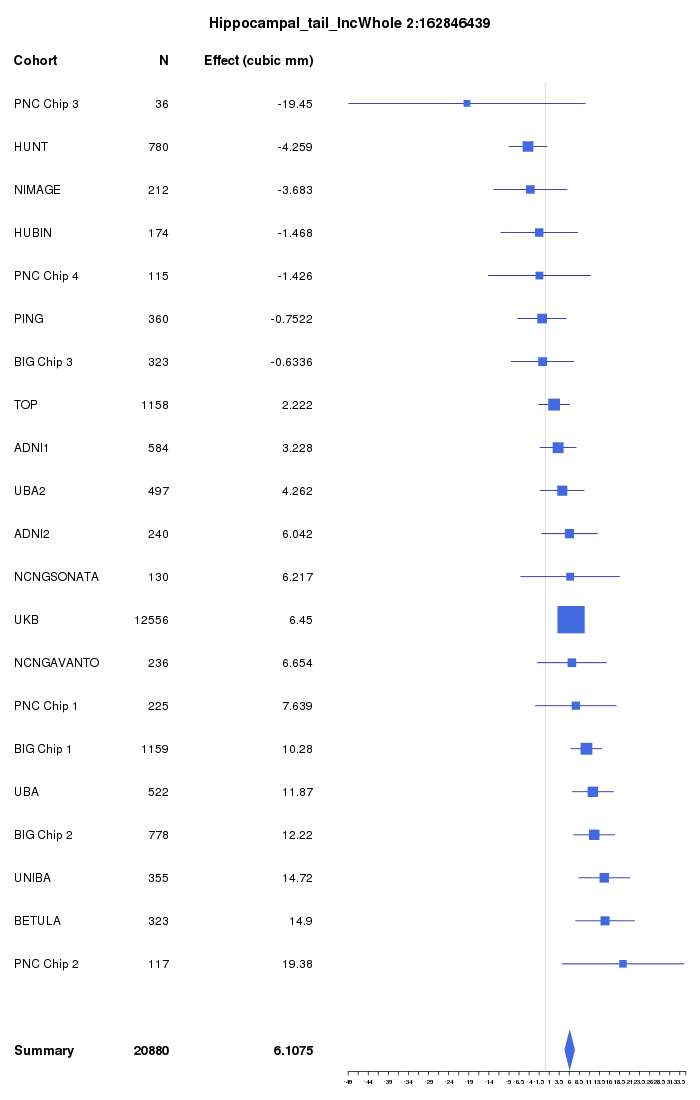

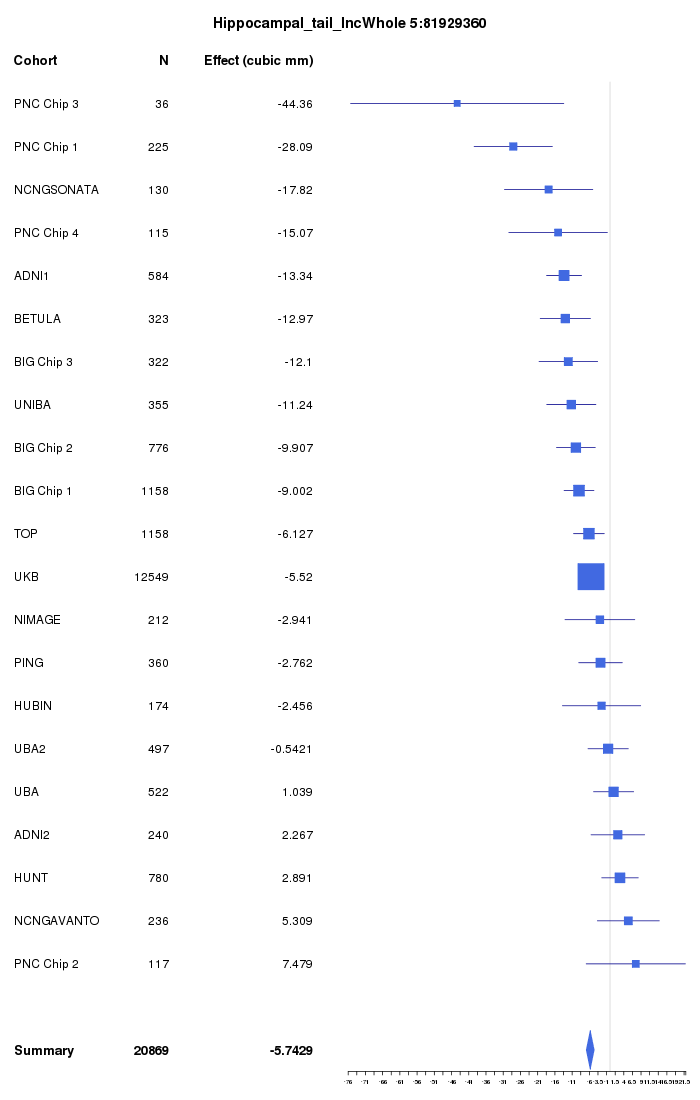

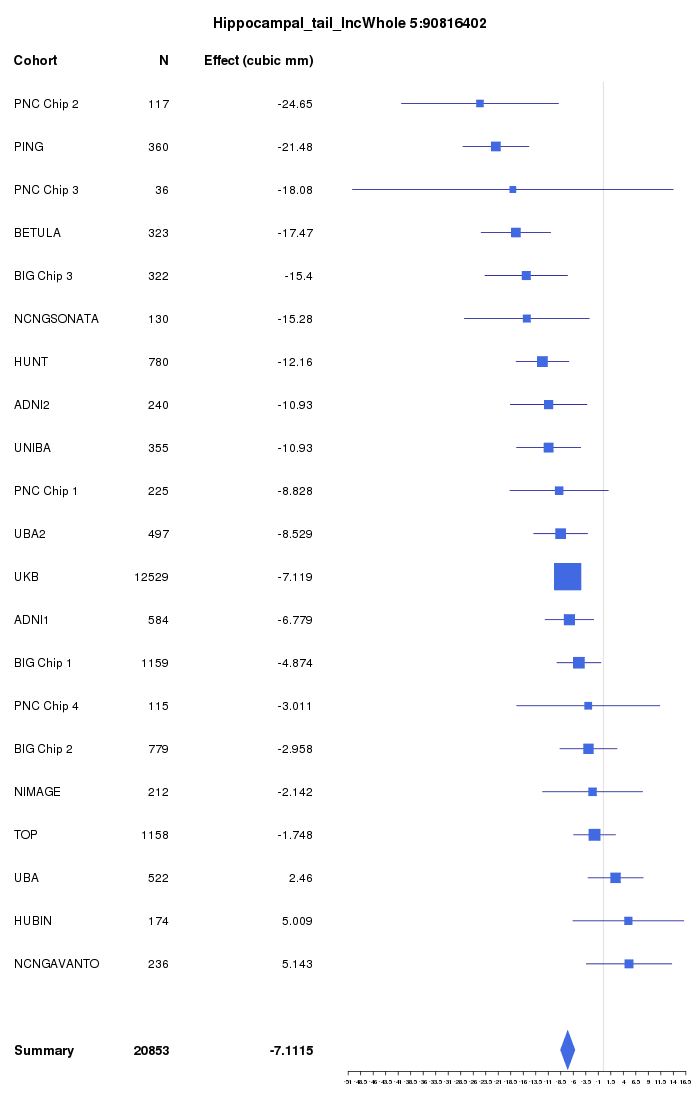

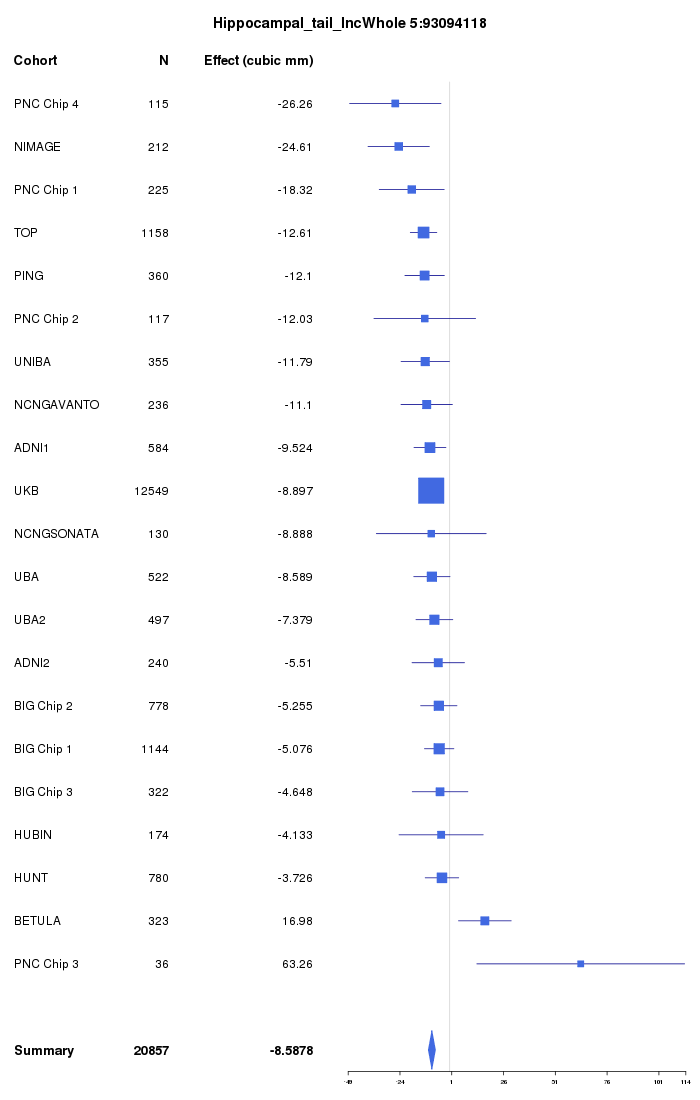

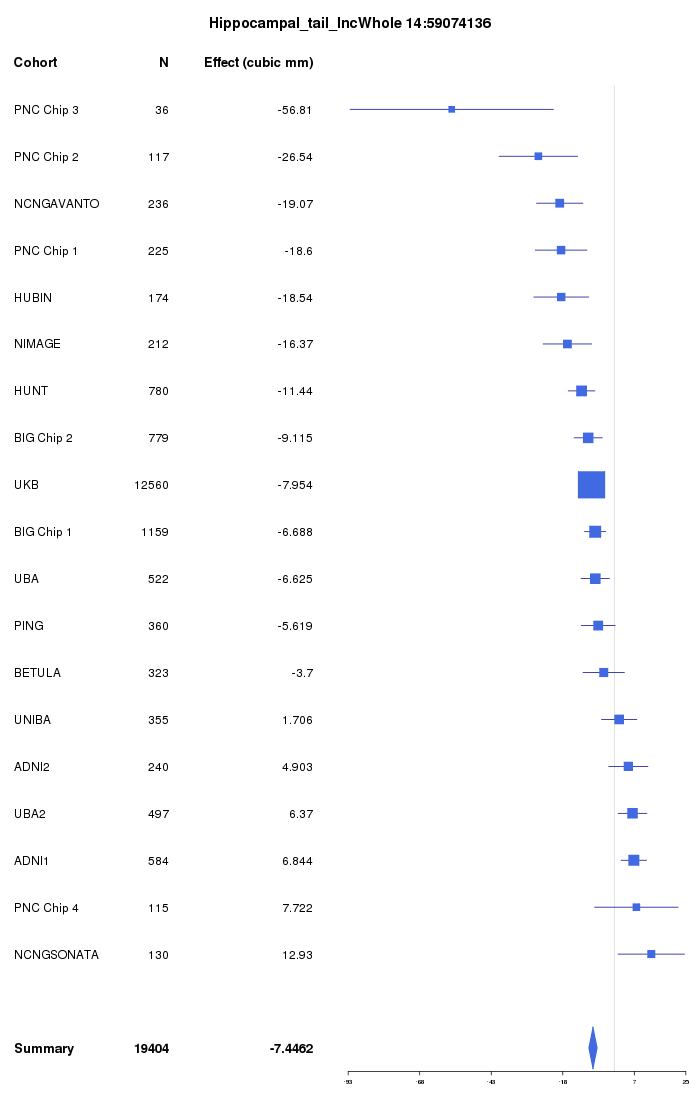

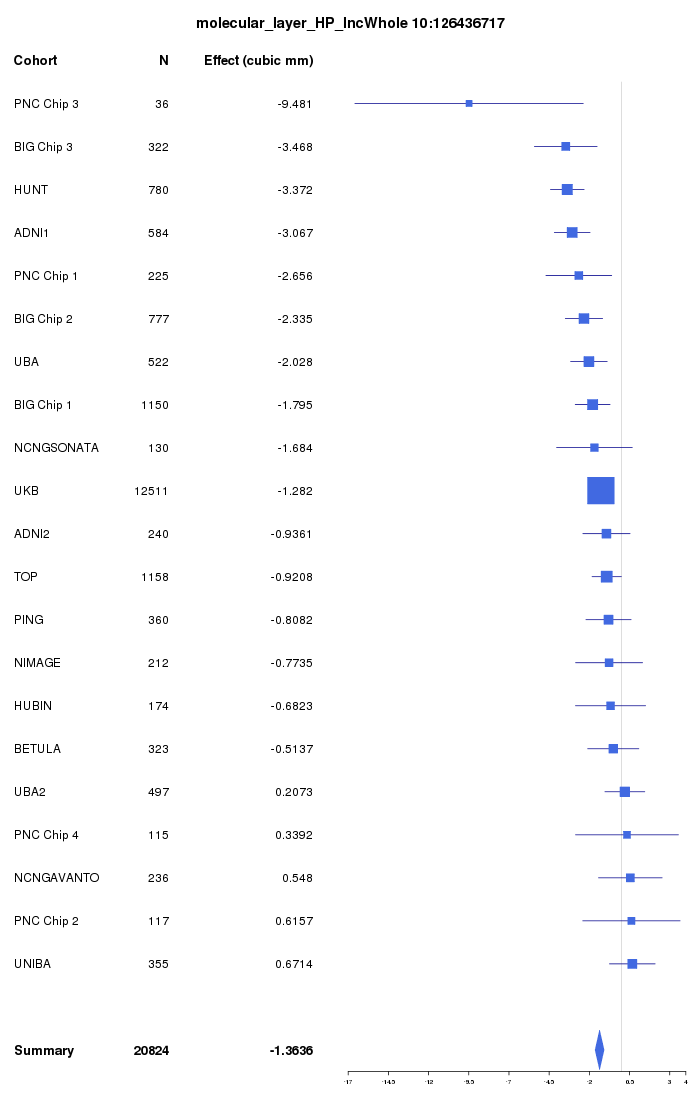

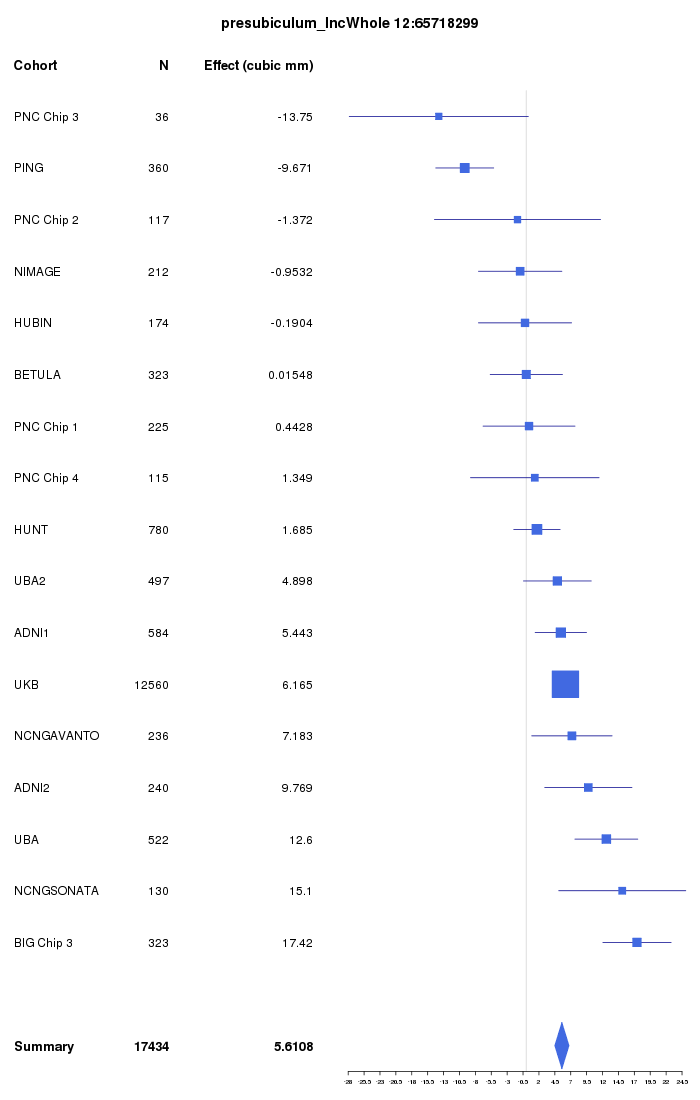

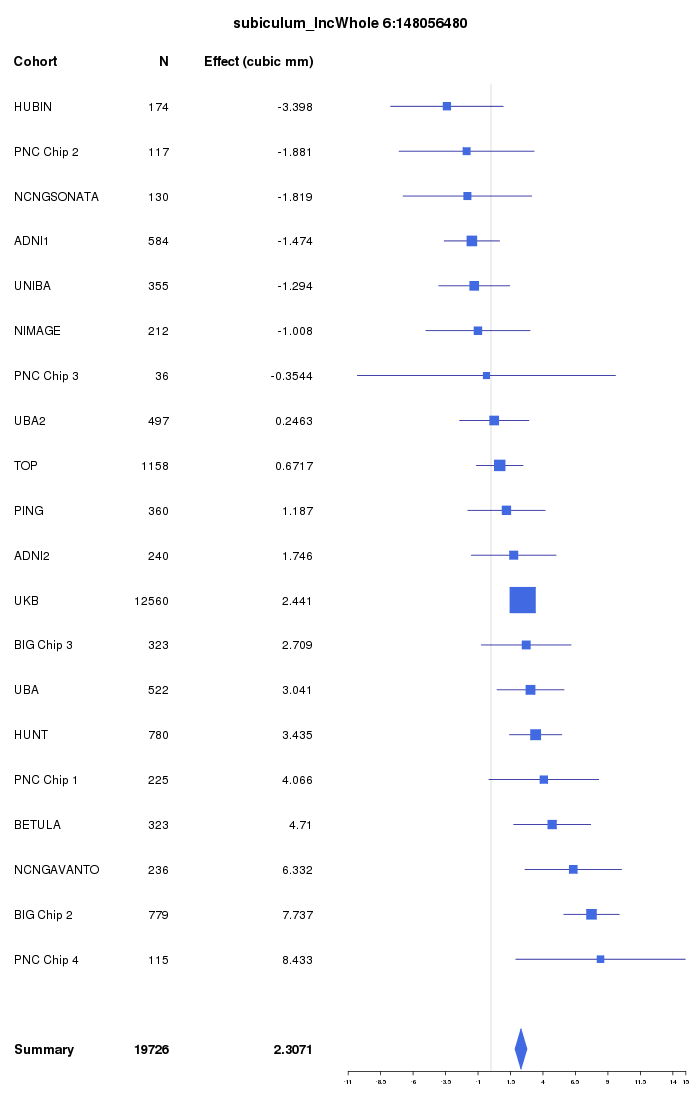

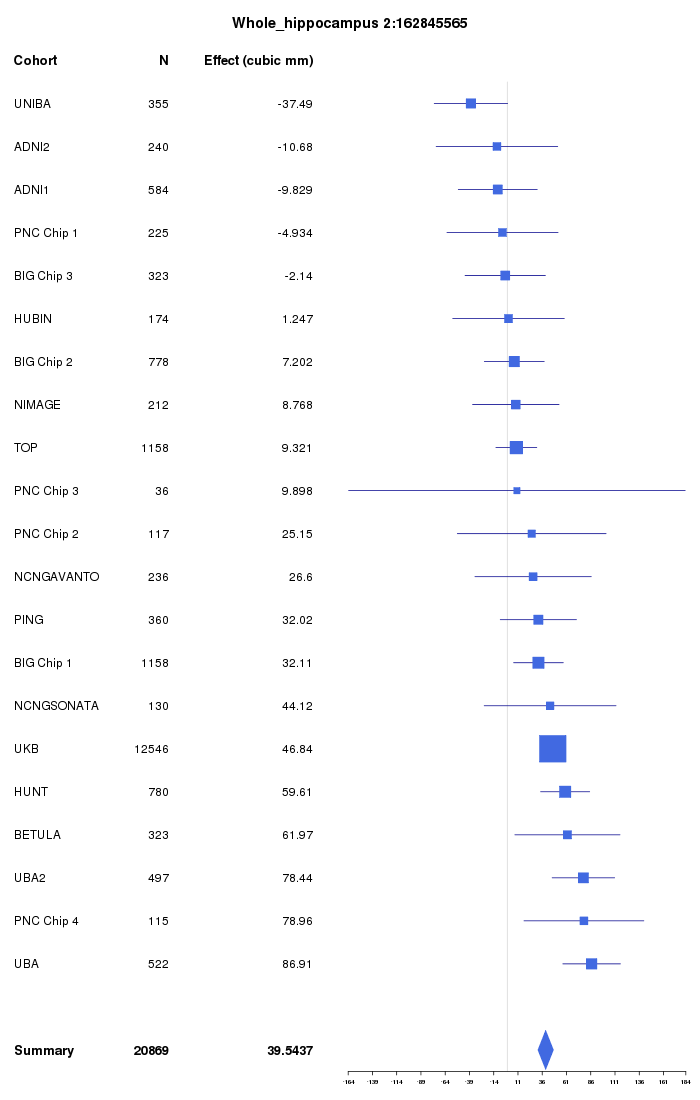

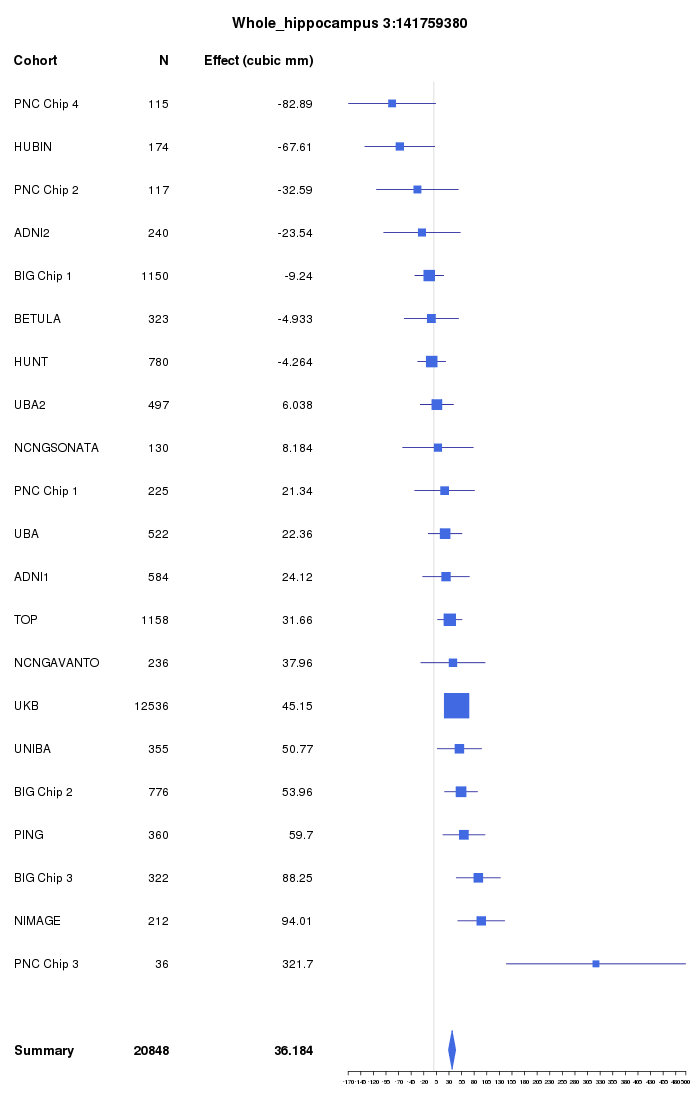

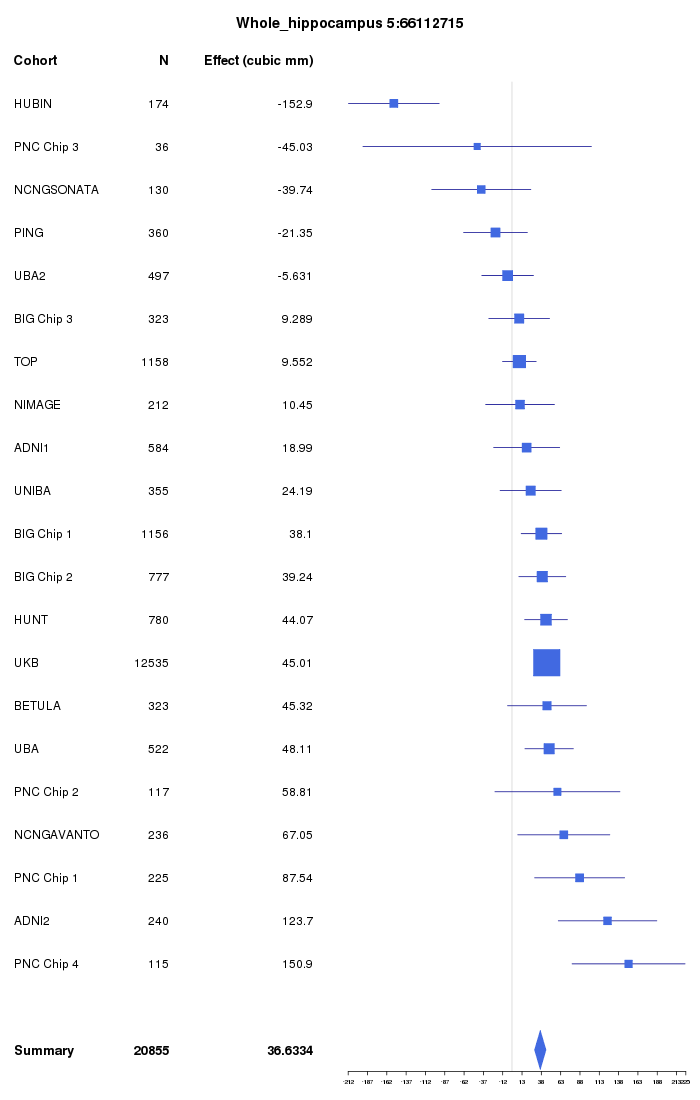

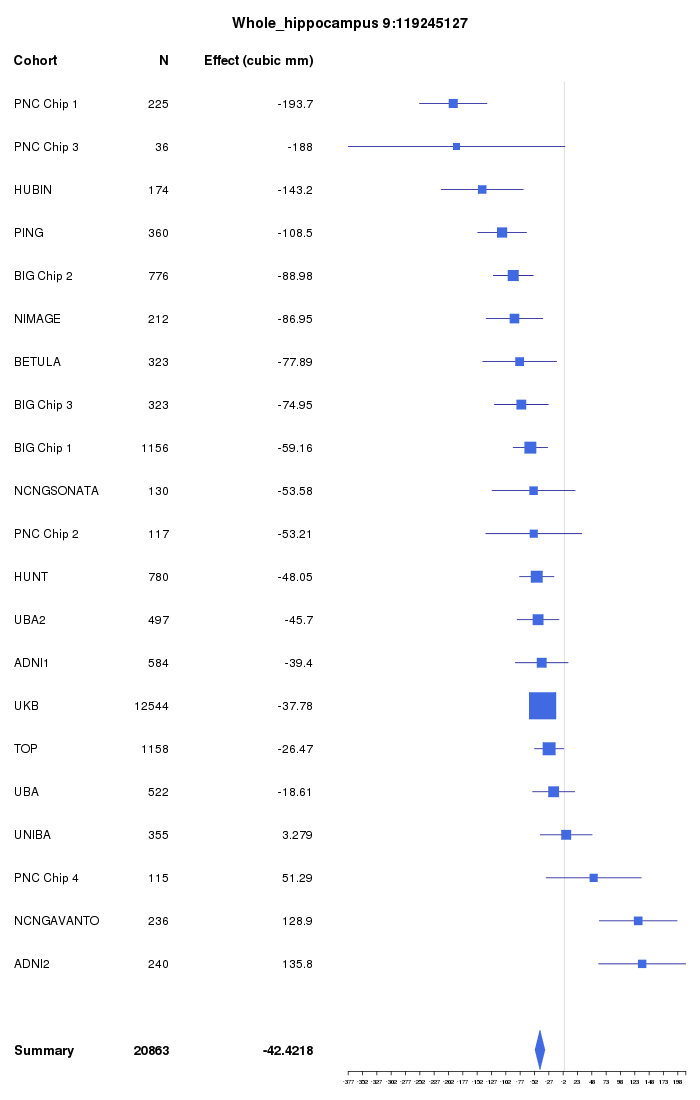

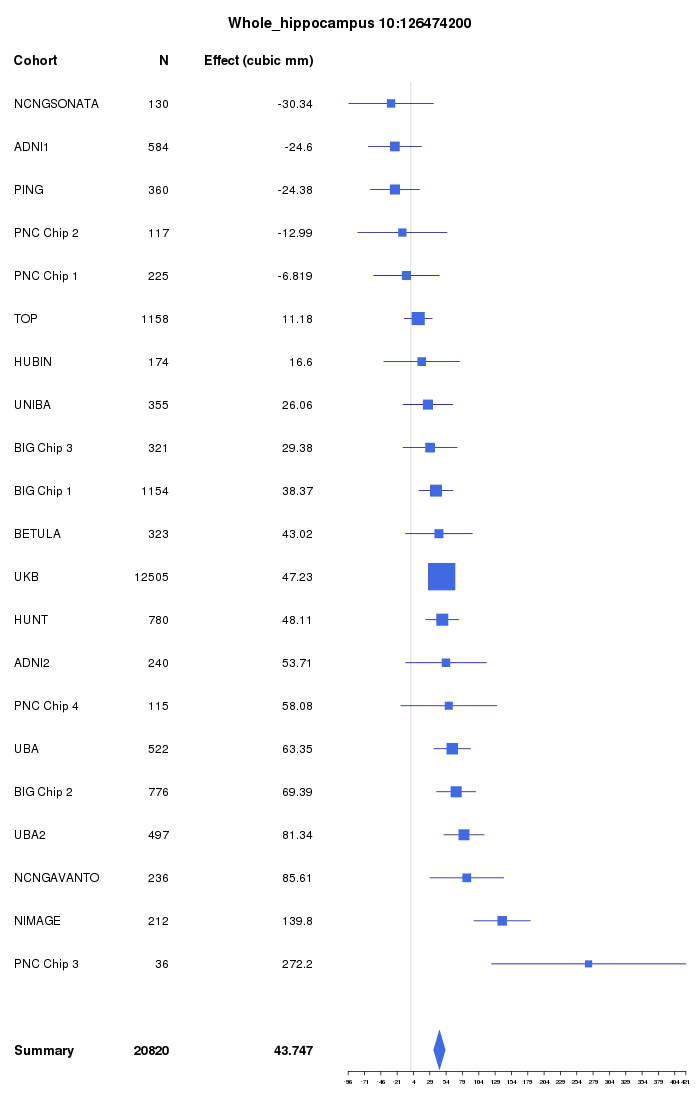

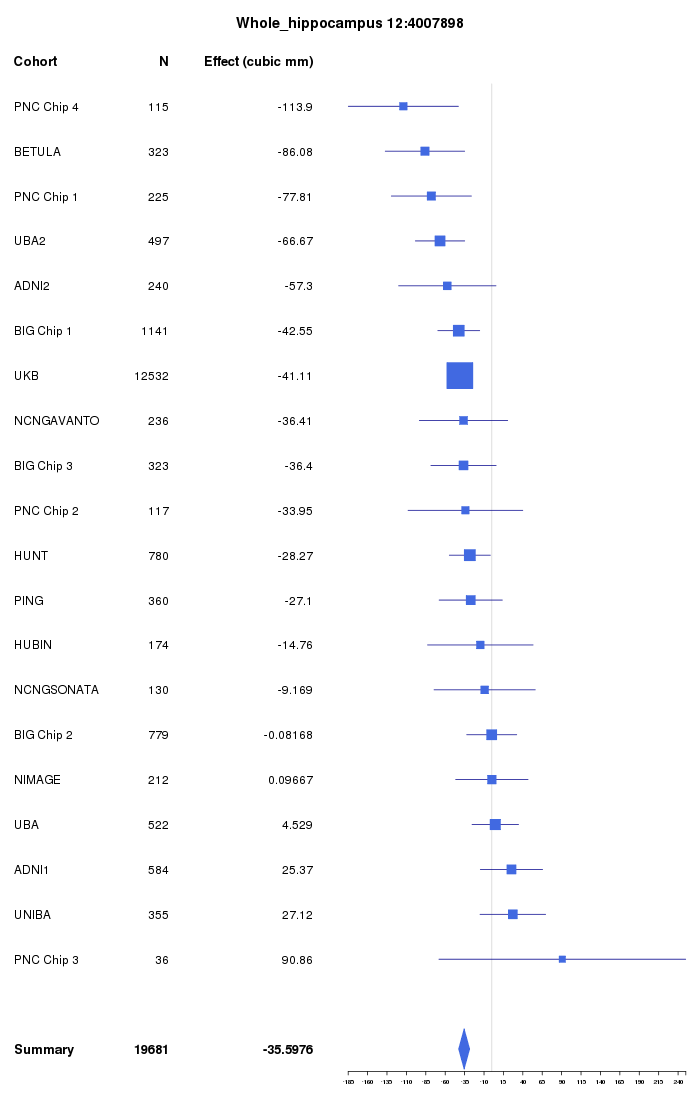

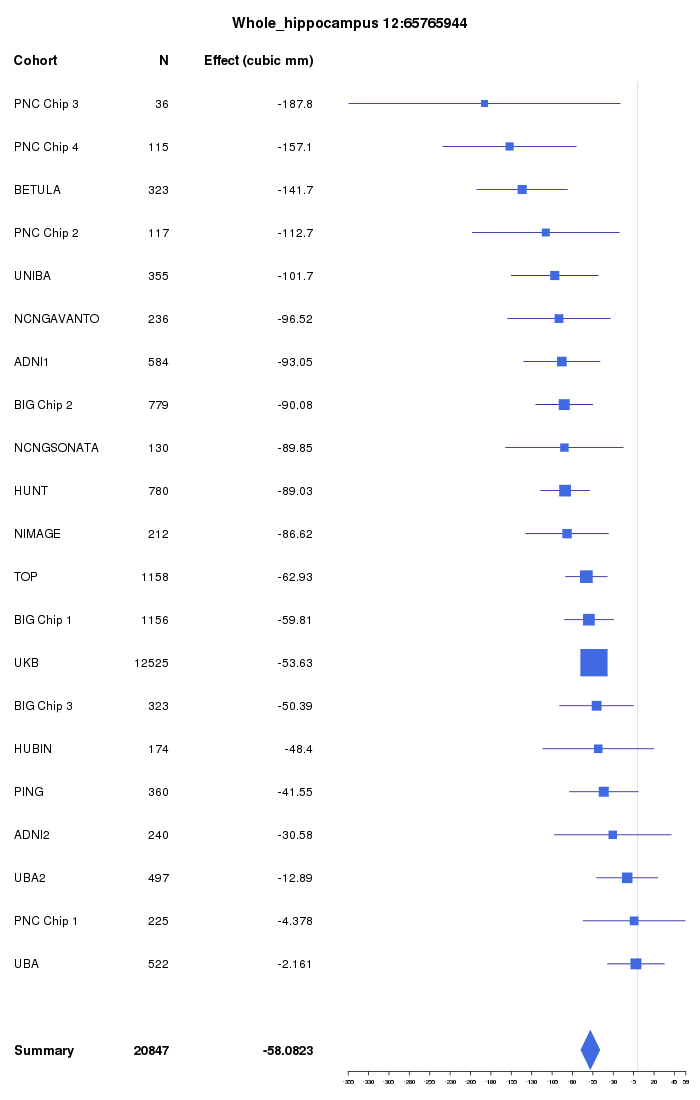

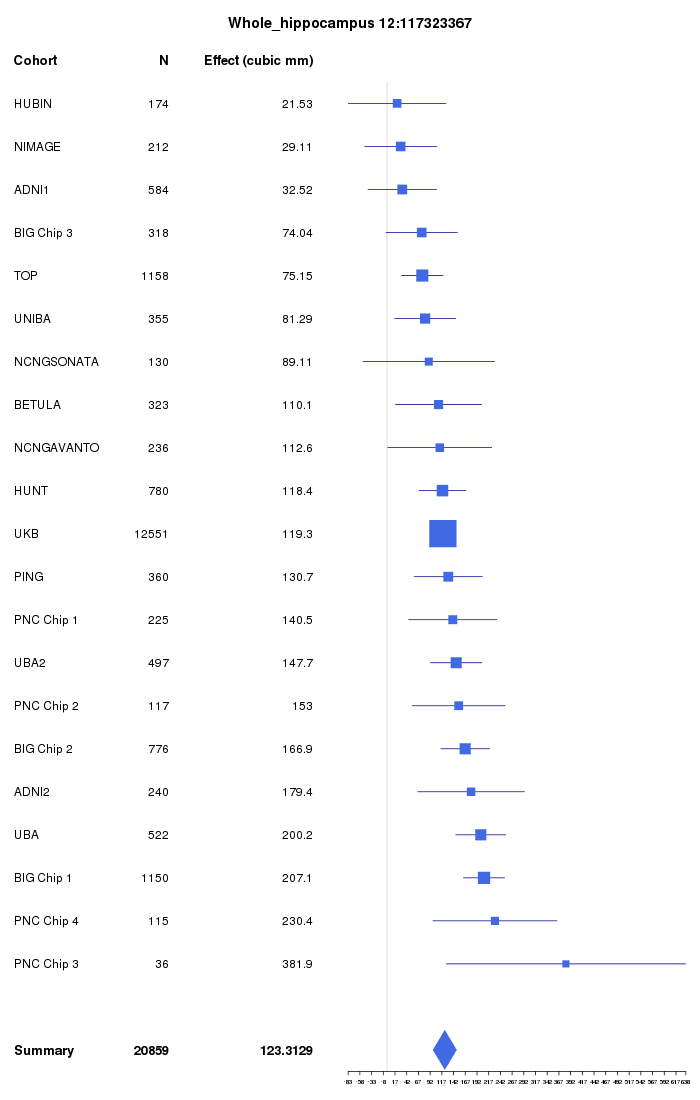


*Linkage disequilibrium score regression (LDSR)*

LDSR utilizes the fact that the effect size of any given SNP relates to its LD structure with causal variants, whereas inflation of test statistics by cryptic relatedness or population stratification will be independent of LD. As such, it can isolate true polygenic signals, expressed in an ‘LD Score’. Cross-trait LDSR builds on this by taking the product of Z-scores from two traits to estimate their genetic overlap. Note that this technique is robust against sample overlap, which is accounted for by the intercept. For more information, please see Bulik-Sullivan *et al.* (22).

*Conditional/conjunctional false discovery rate (FDR)*

Conditional FDR re-ranks test statistics of SNPs for one trait (here, hippocampal subfield volumes) based on their strength of association with a second trait (here, AD and schizophrenia). Genetic overlap can be visualized through conditional QQ-plots, plotting the observed distribution of test statistics from the first trait thresholded at increasing levels of association with the second trait (here we used p<.1, p<.01, and p<.001). Pleiotropic enrichment will show up as increasing deflections from the null distribution, see Figure 3 in the main text and the figures below. Conjunctional FDR can identify specific shared variants, by selecting those SNPs that have a conditional FDR value below .05 on both traits. The strength of these techniques, compared to LDSR, lies in the fact that overlap between the traits is detected regardless of the direction of allelic effects, which could be mixed. For more information, please see Andreassen *et al.* (23,24).

*Figure S5. Conditional QQ plots, conditioning the subfields genome-wide significance statistics on those for schizophrenia and Alzheimer’s disease.*


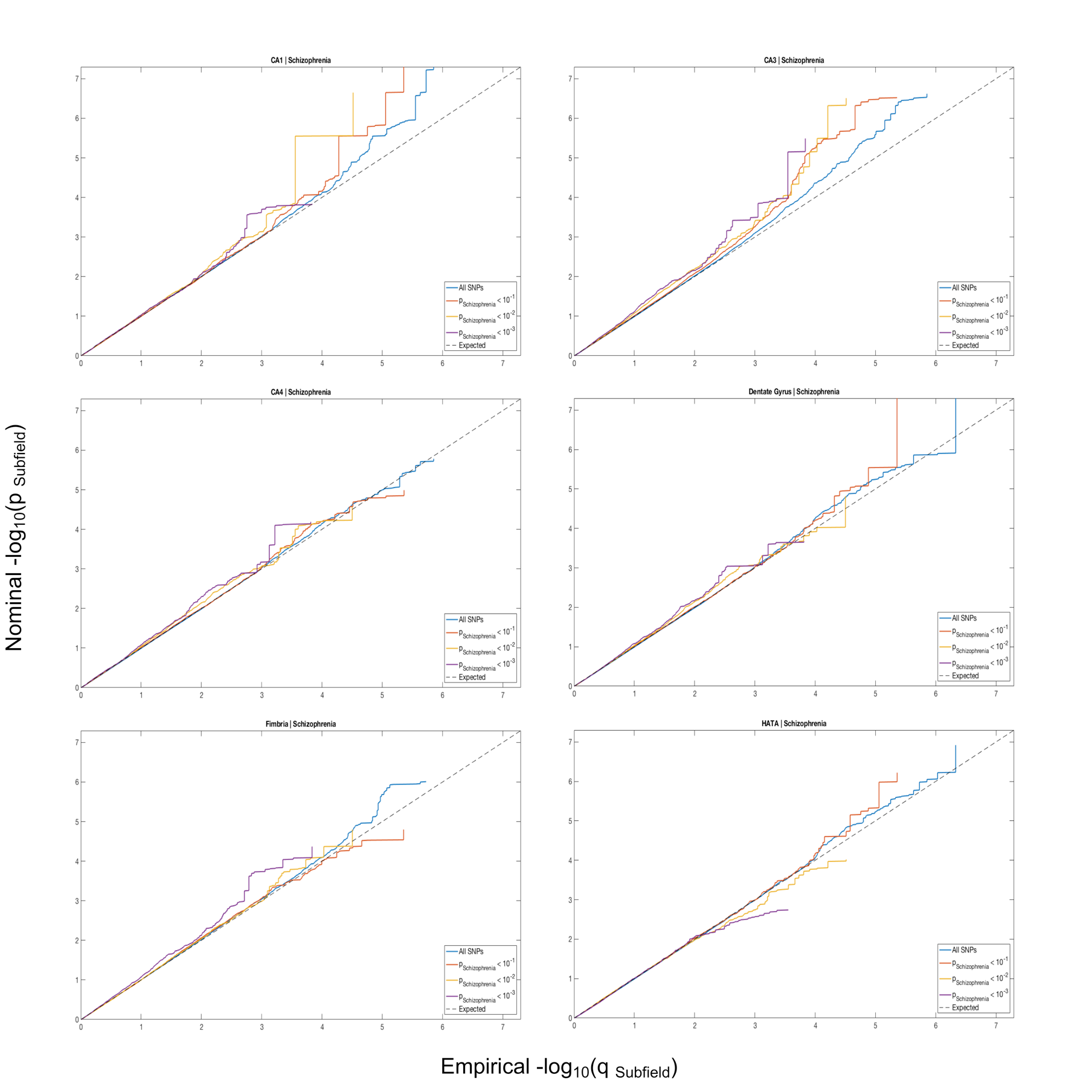


*
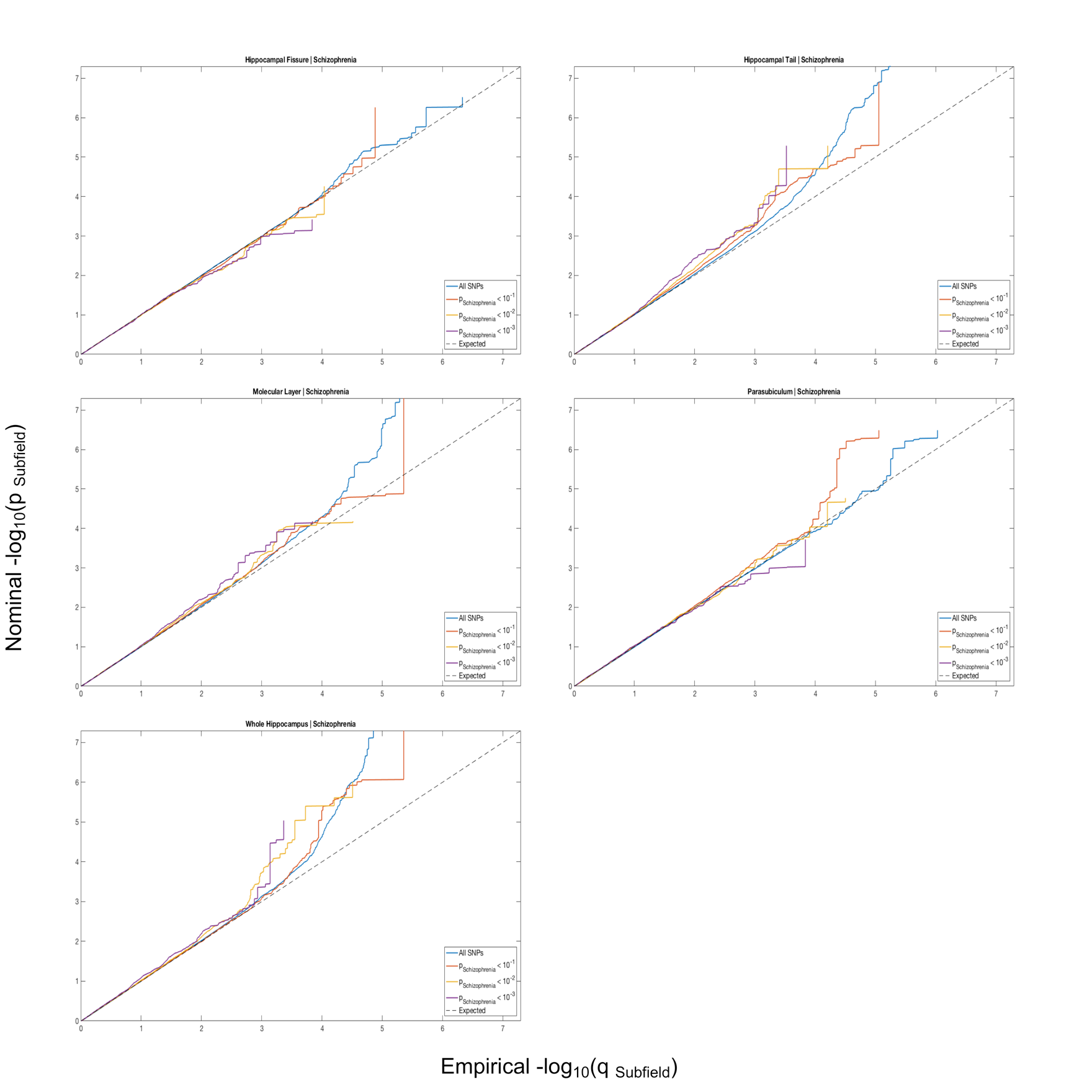
*


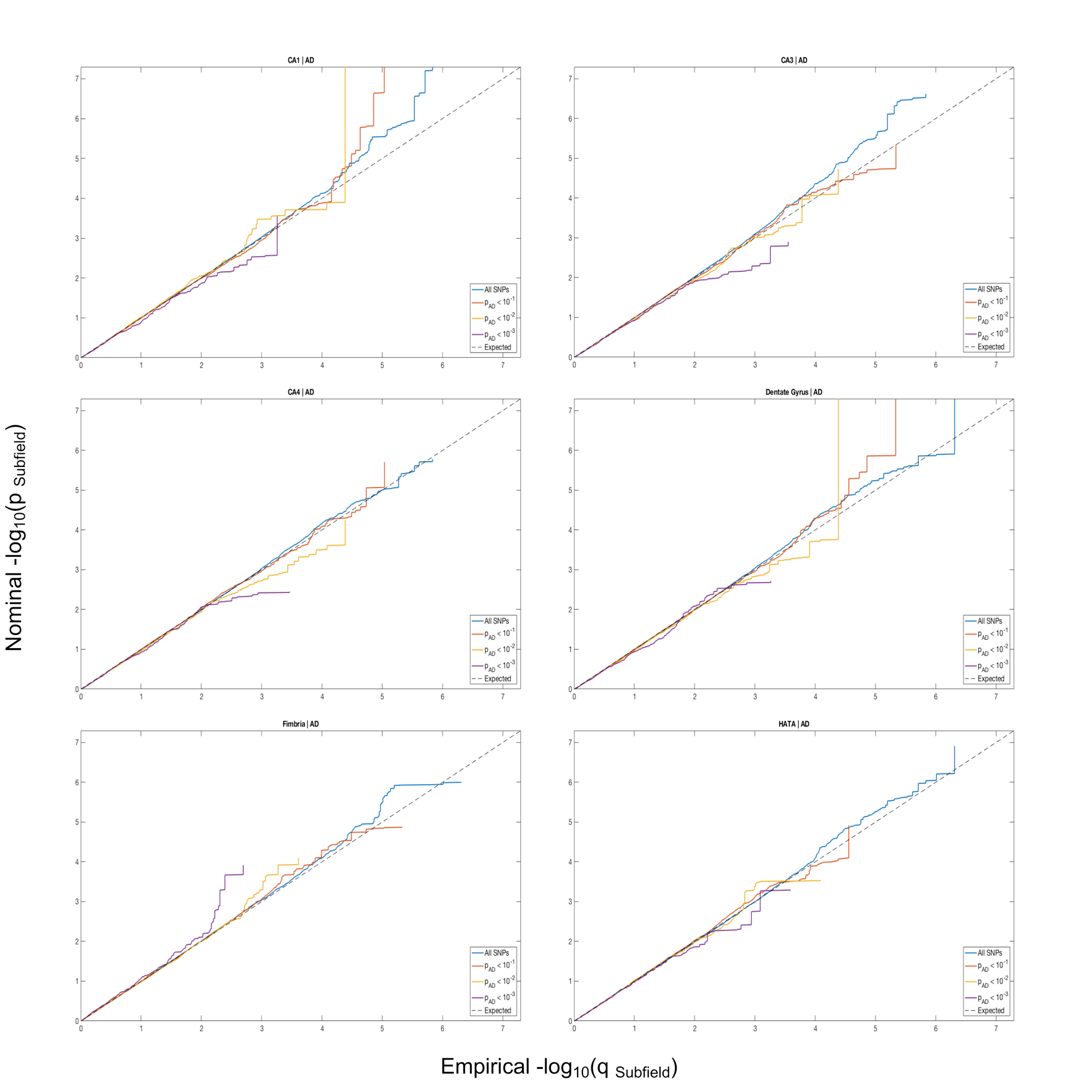


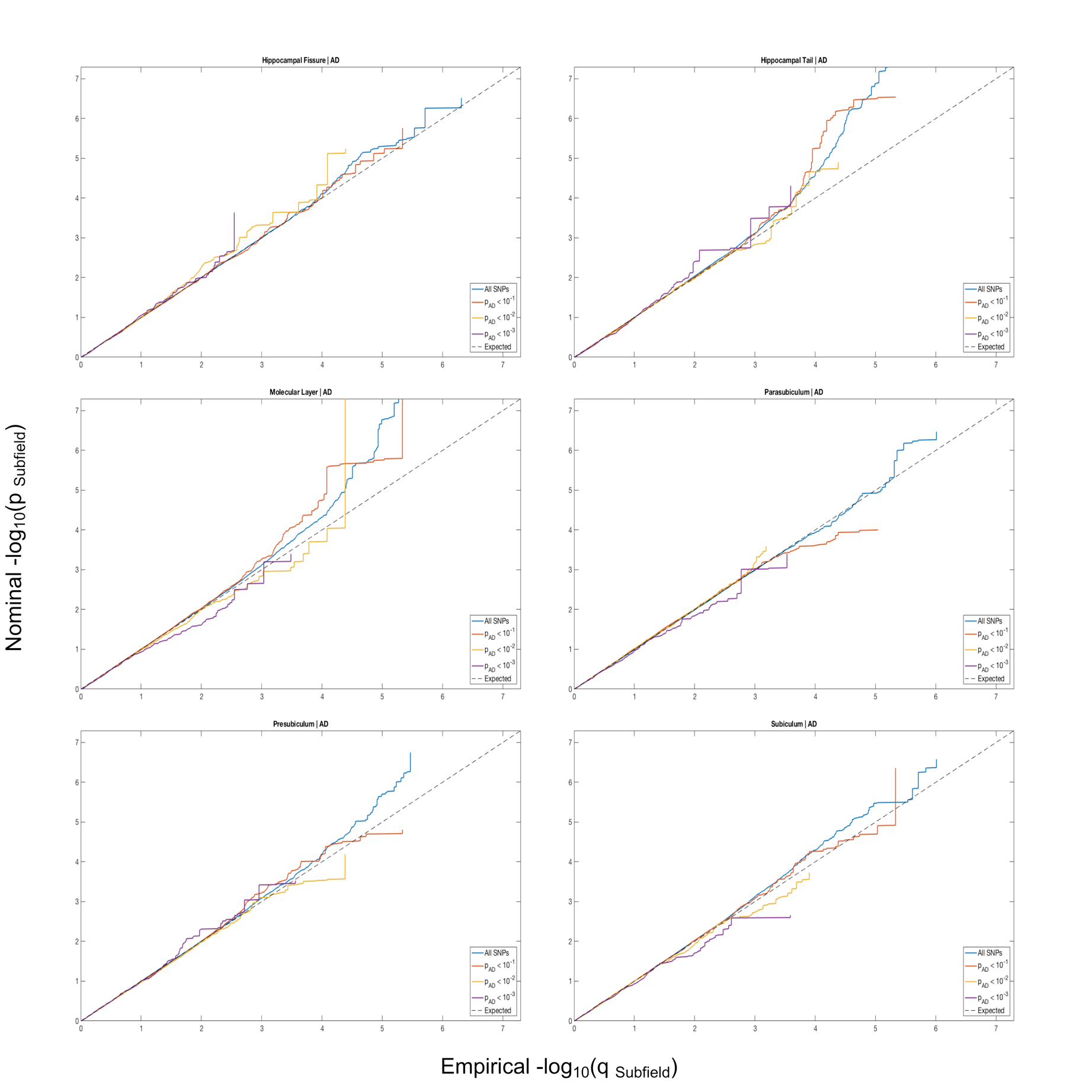


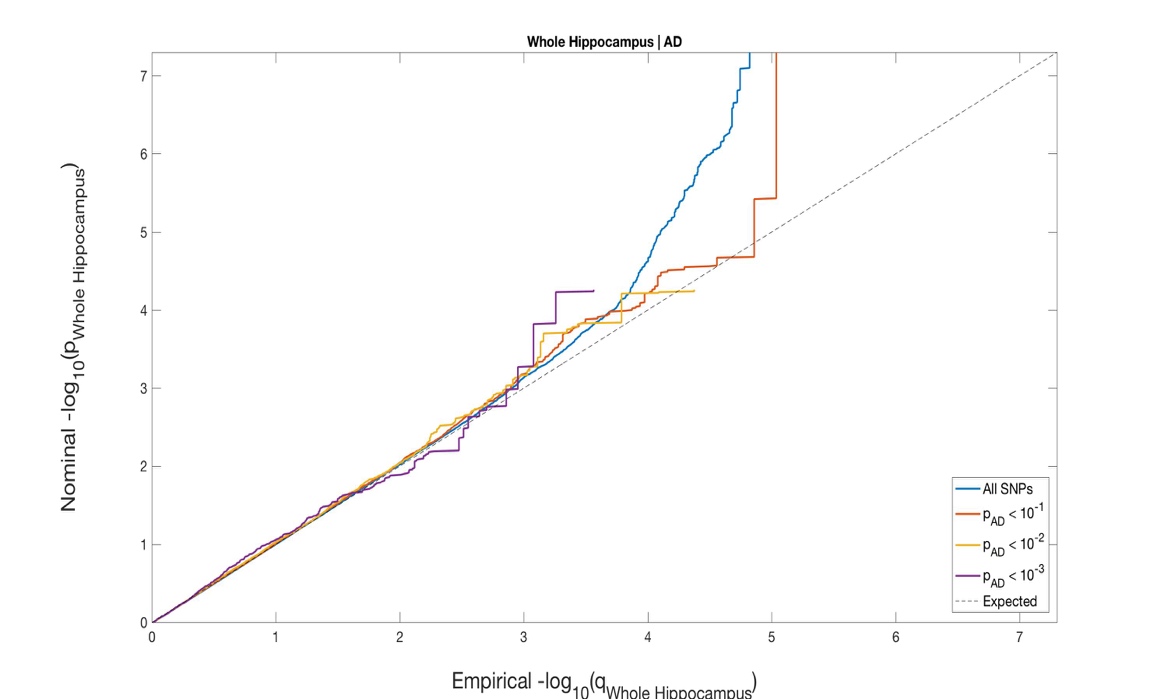


1. Weiner MW, Aisen PS, Jack CR, Jagust WJ, Trojanowski JQ, Shaw L, et al. The Alzheimer’s disease neuroimaging initiative: progress report and future plans. Alzheimer’s Dement J Alzheimer’s Assoc. 2010;6(3):202–11.

2. Wyman BT, Harvey DJ, Crawford K, Bernstein MA, Carmichael O, Cole PE, et al. Standardization of analysis sets for reporting results from ADNI MRI data. Alzheimer’s Dement J Alzheimer’s Assoc. 2013;9(3):332–7.

3. Nilsson L-G, Adolfsson R, Bäckman L, de Frias CM, Molander B, Nyberg L. Betula: A prospective cohort study on memory, health and aging. Aging Neuropsychol Cogn. 2004;11(2–3):134–48.

4. Guadalupe T, Zwiers MP, Teumer A, Wittfeld K, Vasquez AA, Hoogman M, et al. Measurement and genetics of human subcortical and hippocampal asymmetries in large datasets. Hum Brain Mapp. 2014 Jul;35(7):3277–89.

5. Doan NT, Kaufmann T, Bettella F, Jørgensen KN, Brandt CL, Moberget T, et al. Distinct multivariate brain morphological patterns and their added predictive value with cognitive and polygenic risk scores in mental disorders. NeuroImage Clin. 2017;

6. Haukvik UK, Schaer M, Nesvåg R, McNeil T, Hartberg CB, Jönsson EG, et al. Cortical folding in Broca’s area relates to obstetric complications in schizophrenia patients and healthy controls. Psychol Med. 2012;42(6):1329–37.

7. Håberg AK, Hammer TA, Kvistad KA, Rydland J, Müller TB, Eikenes L, et al. Incidental intracranial findings and their clinical impact; The HUNT MRI Study in a general population of 1006 participants between 50-66 years. PLoS One. 2016;11(3):e0151080.

8. Holmen TL, Bratberg G, Krokstad S, Langhammer A, Hveem K, Midthjell K, et al. Cohort profile of the Young-HUNT Study, Norway: a population-based study of adolescents. Int J Epidemiol. 2013;43(2):536–44.

9. Espeseth T, Christoforou A, Lundervold AJ, Steen VM, Le Hellard S, Reinvang I. Imaging and cognitive genetics: the Norwegian Cognitive NeuroGenetics sample. Twin Res Hum Genet. 2012;15(3):442–52.

10. von Rhein D, Mennes M, van Ewijk H, Groenman AP, Zwiers MP, Oosterlaan J, et al. The NeuroIMAGE study: a prospective phenotypic, cognitive, genetic and MRI study in children with attention-deficit/hyperactivity disorder. Design and descriptives. Eur Child Adolesc Psychiatry [Internet]. 2014; Available from: http://download.springer.com/static/pdf/658/art%253A10.1007%252Fs00787-014-0573-4.pdf?originUrl=http%3A%2F%2Flink.springer.com%2Farticle%2F10.1007%2Fs00787-014-0573-4&token2=exp=1447244083~acl=%2Fstatic%2Fpdf%2F658%2Fart%25253A10.1007%25252Fs00787-014-057

11. Jernigan TL, Brown TT, Hagler DJ, Akshoomoff N, Bartsch H, Newman E, et al. The Pediatric Imaging, Neurocognition, and Genetics (PING) Data Repository. Neuroimage [Internet]. 2016 Jan [cited 2017 Jul 3];124:1149–54. Available from: http://linkinghub.elsevier.com/retrieve/pii/S1053811915003572

12. Satterthwaite TD, Connolly JJ, Ruparel K, Calkins ME, Jackson C, Elliott MA, et al. The Philadelphia Neurodevelopmental Cohort: a publicly available resource for the study of normal and abnormal brain development in youth. Neuroimage. 2016;124:1115–9.

13. Satterthwaite TD, Elliott MA, Ruparel K, Loughead J, Prabhakaran K, Calkins ME, et al. Neuroimaging of the Philadelphia neurodevelopmental cohort. Neuroimage. 2014;86:544–53.

14. Dørum ES, Alnæs D, Kaufmann T, Richard G, Lund MJ, Tønnesen S, et al. Age‐related differences in brain network activation and co‐activation during multiple object tracking. Brain Behav. 2016;6(11).

15. Kaufmann T, Alnæs D, Doan NT, Brandt CL, Andreassen OA, Westlye LT. Delayed stabilization and individualization in connectome development are related to psychiatric disorders. Nat Neurosci. 2017;20(4):513–5.

16. Brandt CL, Kaufmann T, Agartz I, Hugdahl K, Jensen J, Ueland T, et al. Cognitive effort and schizophrenia modulate large-scale functional brain connectivity. Schizophr Bull. 2015;41(6):1360–9.

17. Skåtun KC, Kaufmann T, Tønnesen S, Biele G, Melle I, Agartz I, et al. Global brain connectivity alterations in patients with schizophrenia and bipolar spectrum disorders. J psychiatry Neurosci JPN. 2016;41(5):331.

18. Kaufmann T, Skatun KC, Alnaes D, Doan NT, Duff EP, Tonnesen S, et al. Disintegration of Sensorimotor Brain Networks in Schizophrenia. Schizophr Bull. 2015 Nov;41(6):1326–35.

19. Heck A, Fastenrath M, Ackermann S, Auschra B, Bickel H, Coynel D, et al. Converging genetic and functional brain imaging evidence links neuronal excitability to working memory, psychiatric disease, and brain activity. Neuron [Internet]. 2014 Mar 5;81(5):1203–13. Available from: http://www.ncbi.nlm.nih.gov/pmc/articles/PMC4205276/

20. Alfaro-Almagro F, Jenkinson M, Bangerter NK, Andersson JLR, Griffanti L, Douaud G, et al. Image processing and Quality Control for the first 10,000 brain imaging datasets from UK Biobank. Neuroimage. 2018;166:400–24.

21. Pergola G, Trizio S, Di Carlo P, Taurisano P, Mancini M, Amoroso N, et al. Grey matter volume patterns in thalamic nuclei are associated with familial risk for schizophrenia. Schizophr Res. 2017;180:13–20.

22. Bulik-Sullivan B, Finucane HK, Anttila V, Gusev A, Day FR, Loh P-R, et al. An atlas of genetic correlations across human diseases and traits. Nat Genet. 2015;47(11):1236.

23. Andreassen OA, Thompson WK, Dale AM. Boosting the power of schizophrenia genetics by leveraging new statistical tools. Schizophr Bull. 2013;40(1):13–7.

24. Andreassen OA, Thompson WK, Schork AJ, Ripke S, Mattingsdal M, Kelsoe JR, et al. Improved detection of common variants associated with schizophrenia and bipolar disorder using pleiotropy-informed conditional false discovery rate. PLoS Genet. 2013;9(4):e1003455.
